# *ycf1*, the most promising plastid DNA barcode of land plants

**DOI:** 10.1038/srep08348

**Published:** 2015-02-12

**Authors:** Wenpan Dong, Chao Xu, Changhao Li, Jiahui Sun, Yunjuan Zuo, Shuo Shi, Tao Cheng, Junjie Guo, Shiliang Zhou

**Affiliations:** 1 State Key Laboratory of Systematic and Evolutionary Botany, Institute of Botany, Chinese Academy of Sciences, Beijing 100093, China; 2 University of Chinese Academy of Sciences, Beijing 100049, China; 3 Research Institute of Tropical Forestry, the Chinese Academy of Forestry, Gongdong, Guangzhou 510520, China

## Abstract

A DNA barcode is a DNA fragment used to identify species. For land plants, DNA fragments of plastid genome could be the primary consideration. Unfortunately, most of the plastid candidate barcodes lack species-level resolution. The identification of DNA barcodes of high resolution at species level is critical to the success of DNA barcoding in plants. We searched the available plastid genomes for the most variable regions and tested the best candidates using both a large number of tree species and seven well-sampled plant groups. Two regions of the plastid gene *ycf1*, *ycf1*a and *ycf1*b, were the most variable loci that were better than existing plastid candidate barcodes and can serve as a barcode of land plants. Primers were designed for the amplification of these regions, and the PCR success of these primers ranged from 82.80% to 98.17%. Of 420 tree species, 357 species could be distinguished using *ycf1*b, which was slightly better than the combination of *matK* and *rbcL*. For the well-sampled representative plant groups, *ycf1*b generally performed better than any of the *matK*, *rbcL* and *trnH-psbA*. We concluded that *ycf1*a or *ycf1*b is the most variable plastid genome region and can serve as a core barcode of land plants.

DNA barcoding is a technique used to identify unknown materials of known species based on DNA sequences of standard genome regions (i.e. DNA barcodes)^1^. Before this technique can be fully utilised, a barcode that is variable enough to discriminate between species of interest and a reliable barcode reference library must be made available. The first of these requirements is more significant as it is relatively easy to build a reference library if DNA materials already exist. Ideally, a barcode should be variable enough to resolve closely related species and short enough for easy experimental manipulation and low cost. The sequences flanking the barcode should be conservative enough to facilitate the design of universal primers for high PCR and sequencing success. Presently, the candidate barcodes are selected from markers used in molecular systematics due to the limited knowledge of genome variations; we know little about mitochondrial genomes, much less nuclear genomes. Fortunately, by the end of 2013, 429 plastid genomes were sequenced, and perhaps they contain a useful plant barcode.

In the past decade, several plastid genome regions such as *atpF-H*, *matK*, *psbK-I*, *rbcL*, *ropC1*, *rpoB*, *trnH-psbA*, and *trnL-F* that are frequently used in plant molecular systematics have been extensively evaluated^2^,^3^,^4^, and the *rbcL* and *matK* genes were selected as core plant barcodes by the CBOL Plant Working Group^5^. Unfortunately, *rbcL* seems to be more suitable for barcoding lower plants than for seed plants^6^. One of the most highly variable regions of the plastid genome, *trnH-psbA*, undergoes chromosomal rearrangements (e.g., inversions and microsatellite loci) and contains a limited number of informative sites due to its short length^7^,^8^. Recently, Dong *et al.* found that two regions of the plastid gene *ycf1* were very variable in flowering plants^9^.

As the second largest gene in the plastid genome, *ycf1* encodes a protein of approximately 1,800 amino acids. Recent experiments showed that *ycf1* is essential for plant viability and encodes Tic214, a vital component of the *Arabidopsis* TIC complex^10^. Within the plastid genome, *ycf1* spans the small single copy (SSC) and the inverted repeat (IR) regions. The section of *ycf1* in the IR region is short (less than one kilobase long) and conserved. In contrast, the section of *ycf1* in the SSC region has high sequence variability in seed plants. This region of the *ycf1* gene is more variable than *matK* in most taxa investigated thus far^11^,^12^ and has been used in molecular systematics at low taxonomic levels^13^,^14^,^15^,^16^,^17^. Two regions within *ycf1*, *ycf1a* and *ycf1b*, have been predicted to have the highest nucleotide diversity (π) at the species level within angiosperm plastid genomes^9^.

Because *ycf1* is too long (5709 bp in *Nicotiana tabacum*) and too variable to permit the design of universal primers^9^, it has received little attention for DNA barcoding or molecular systematic purposes at low taxonomic levels; however, the high variability of *ycf1* indicates its potential value in DNA barcoding of land plants. This paper reports the results of our evaluations of the *ycf1* gene for DNA barcoding purposes. We (1) generated primers for PCR amplification of *ycf1*a and *ycf1*b, and (2) tested the performance of *ycf1*a or *ycf1*b in discriminating between species compared with the plastid genes *rbcL*, *matK* and *trnH-psbA*.

## Results

### The *ycf1*a and *ycf1*b regions are the most variable regions of the *ycf1* gene

According to Dong et al.^9^, angiosperms have two highly variable regions in the *ycf1* gene, *ycf1*a and *ycf1*b. These results were verified using 136 genomes belonging to 27 genera (see the electronic supplementary material, Table S1). The exact positions of *ycf1*a vary slightly among plant groups, while the positions of *ycf1*b are quite consistent, especially in seed plants.

### Primer universality

The *ycf1* gene is too variable for the design of universal primers for all land plants; however, we were able to design universal primers for Bryophytes, Monilophytes, gymnosperms and angiosperms (Table 1). The ycf1mF/ycf1mR primer pair amplified the expected fragments in 32 of 34 (94.12%) Bryophyte families (see the electronic supplementary material, Table S2).

The *ycf1*b regions of Monilophytes are too divergent to contain satisfactory primer sites; in contrast, the sequences flanking *ycf1*a are relatively conserved and we designed three primers (one forward and two reverse primers, Table 1). Using these primers, *ycf1*a fragments were amplified from 82.80% of the samples belonging to 93 genera in 42 families (see the electronic supplementary material, Table S2).

The ycf1gF/ycf1gR primer pair was designed for conifers and cycads (see the electronic supplementary material, Table S2). The divergence of *ycf1* sequences in gymnosperms is remarkable, and it is difficult to identify a single pair of universal primers. Consequently, the species-rich conifers required special attention.

The ycf1bF/ycf1bR primer pair worked the best for angiosperms. The PCR success reached 98.17% samples from 219 genera in 217 families (see the electronic supplementary material, Table S2). Moreover, critical mutations at the 3′ end that would cause amplification failure were observed in some taxa. To minimise PCR failures, some substitutions to the universal primers listed in Table 1 were generated for 131 families (see the electronic supplementary material, Table S3) in the event that the universal primers failed.

### Performance of *ycf1* in identifying BBG woody plants

In total, we obtained 1352 sequences of *matK*, *rbcL* and *ycf1* from 420 woody plant species representing 179 genera in 76 families. The sequence recoveries for *rbcL*b, *matK*, and *ycf1*b were 99.18%, 91.43%, and 85.31%, respectively (see the electronic supplementary material, Table S4). Poor PCR amplification of *ycf1*b was encountered for *Lonicera* (13 samples) and *Berberis* (15 samples). When samples from these two genera were excluded, the *ycf1*b sequence recovery reached 90.48%. Approximately 79.80% (391) of all samples contained all three sequenced fragments. Therefore, two types of datasets were generated for accurate assessments. One comprised all sequences for each marker, and the other comprised the samples with all three markers. Three two-barcode combinations and one three-barcode combination were also tested for each dataset type.

**Figure 1:**
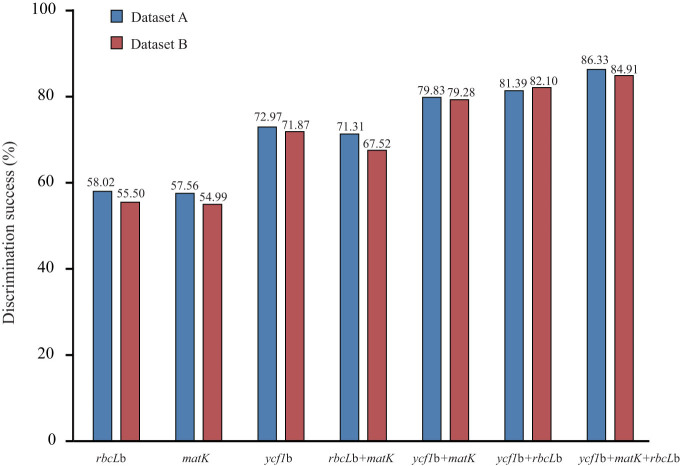
Performances of *matK*, *rbcL*b and *ycf1*b in resolving BBG tree species using BLAST method. Dataset A includes all 490 samples and dataset B includes 391 samples having all three barcode sequences.

For the datasets of all sequences, *ycf1*b showed the highest discriminatory power of the three barcodes, discriminating 73.97% of all the species. The barcodes *rbcL*b (58.02%) and *matK* (57.56%) had similar discriminatory power (Fig. 1), much lower than *ycf1*b. The performance of *ycf1*b was slightly better than the combination of *rbcL*b and *matK* (71.31%). Combining *ycf1*b with either *rbcL*b or *matK* increased the discriminatory power to 81.39% and 79.83%, respectively, and the use of all three candidates increased the discriminatory power to 86.33%.

To eliminate the possible effects of sample inequity on the different markers, we used datasets of 391 samples with all three markers to increase the reliability of direct comparisons of species resolution among the markers. Similar patterns were observed for these datasets. *ycf1*b had the highest species resolution among the three markers at 71.87%, with 54.99% resolution for *matK* and 55.50% resolution for *rbcL*b (Fig. 1).

### Performance of *ycf1* in identifying species within seven well-sampled plant groups

Using *matK*, *rbcL*b and *trnH-psbA* as controls, seven relatively well-sampled plant groups were chosen to test the discriminatory powers of *ycf1*b (see the electronic supplementary material, Table S5). The lengths of *matK*, *rbcL*b and *ycf1*b varied among the plant groups. The poly-A/T tracts longer than 9 bp were observed in the *trnH-psbA* sequences from six of the seven plant groups, and non-homologous inversions of 2 to 21 bp were observed in five groups. The nucleotide diversity (π) of *ycf1*b was the highest among the four markers in the five plant groups (see the electronic supplementary material, Table S6–S12).

**Figure 2:**
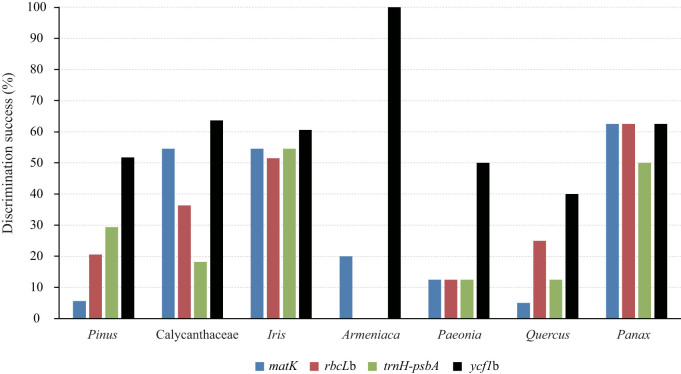
Performances of *matK*, *rbcL*b*, trnH-psbA* and *ycf1*b in resolving species in seven well-sampled plant groups representing gymnosperms, basal angiosperms, monocots, Saxifragales, rosids, and asterids.

Using the distance method, *ycf1*b exhibited the highest discriminatory power among the four markers in six of the seven plant groups (Fig. 2). Combinations of *matK*, *rbcL*b and *trnH-psbA* did not typically increase the discriminatory rates; in contrast, the combination of *ycf1*b with either *matK*, *rbcL*b or *trnH-psbA* increased the percentage of discrimination success by varying degrees for five of the seven plant groups (Fig. 2, and see the electronic supplementary material, Fig. S1, Table S6–S12). Without *ycf1*b, *matK* and *rbcL*b did not contribute for either *Armeniaca* or *Paeonia* (see the electronic supplementary material, Fig. S1).

## Discussion

### Which gene can serve as a barcode of plants?

The question of which gene can serve as a barcode for plants remains to be answered even though the combination of *matK* and *rbcL* has been suggested as the core barcode of land plants^5^. Marker selection is critically important as time and money can be saved by the quick identification of a suitable barcode. Kress and Erickson had three criteria^18^, Ford et al. put forward five considerations^19^, and the CBOL Plant Working Group placed higher importance on experimental considerations. The definition of DNA barcode^1^ implies that the first consideration should be species resolution. The issue of a barcoding gap is not an important issue in DNA barcoding; rather, it is an important question in systematics and taxonomy. Many DNA barcoders are also systematists and the two issues have been investigated simultaneously. If a species is correctly circumscribed, the optimal barcode will show the highest probability to distinguish it from other congeners regardless of the existence of a barcoding gap. Unfortunately, it is difficult to predict when such an ideal barcode might be found. We can now attempt to identify an improved barcode while also using the old ones for some time and then eventually substitute them.

### Imperfections of the existing candidates

Many candidate barcodes that cannot withstand tests of universality are only suitable for specific plant groups and are falling from common use. Only four continue to receive some use: the chloroplast genes *rbcL*, *matK* and *trnH-psbA*, and the nuclear internal transcribed spacer (ITS). The *rbcL* gene was suggested as a core barcode not as a result of its power in barcoding species, but rather its historical popularity^19^ and possibly experimental ease. The *rbcL* gene has been subject to considerable criticism as a barcode for seed plants^20^,^21^,^22^,^23^; however, it may be useful for lower plants^6^.

Although they are advocates of *matK*, Ford and his colleagues acknowledged that *matK* had only modest performance^19^. The *matK* gene was not among the top 10 most variable species-level markers^9^. Although *matK* is more useful than *rbcL*^6^, in most cases it is not the only useful species-level barcode^23^,^24^.

The use of *trnH-psbA* had been well evaluated^8^, and although *trnH-psbA* is more variable than either *matK* or *rbcL*, several problems limit its widespread adoption. The extensive prevalence of inversions and insertions within species, long polystructures that cause sequencing difficulties, and relatively short lengths prevent its use as a core barcode.

After a few years of disfavour, the nuclear ribosomal ITS first proposed by Kress et al.^25^ has again become accepted as a core barcode, as exemplified by studies using large data^20^,^26^,^27^,^28^. With the exception of *ycf1*, ITS has been shown to have unparalleled species resolution compared with the candidate barcodes proposed thus far; however, it suffers from incomplete concerted evolution in some cases and from experimental complexity for species of hybrid origin.

### Is *ycf1* good enough to be a barcode of land plants?

It is easy to identify the most variable regions in certain taxa at the species level; however, it is difficult, and even unlikely, to identify such regions in all taxa. With a few isolated exceptions, the *ycf1*a and *ycf1*b regions are perhaps the most variable regions in most taxa^9^. A barcode should be chosen because it shows the highest species resolution in most cases rather than in specific cases. The *ycf1* gene meets this criterion and can serve as a barcode of land plants.

Currently, there are two applications for DNA barcoding. One application is for flora, and the other is for specific taxa. The first application is exemplified by barcoding trees in large ecological plots^29^,^30^,^31^,^32^, and a similar example was given in this study. Our test example differs from the barcoding of local flora in that more species are from the same genera, which may show reduced discriminating power. The *ycf1*b performed satisfactorily when compared to the core barcode combination of *matK* and *rbcL*. The second application is becoming increasingly popular for barcoding of medicines, teas, and foods, etc^33^,^34^. As *ycf1* was not previously identified as a potential barcode, comparisons are not currently available. In this paper, seven examples were used to show the superior performance of *ycf1*b compared with other barcodes for distantly related plant groups. Consequently, *ycf1*b is expected to be suitable for an extensive group of plants.

The *ycf1* gene was slowly identified for its potential use as a barcode most likely due to its length and lack of universal primers; however, a few phylogenetic applications had been found for Pinaceae^14^,^35^, Orchidaceae^13^, Lamiaceae^15^,^36^ and *Prunus*^37^.

One major concern for the use of *ycf1* as a barcode is the absence of *ycf1* in some taxa. The *ycf1* gene is functional and is not commonly lost^38^. It was erroneously reported to have been lost from Acorales, Poales, and *Passiflora*; however, it is only absent from Poaceae^39^,^40^.

### Experimental considerations for *ycf1* use

Primer universality is an important criterion for an ideal DNA barcode. The primers for *rbcL*b were recently optimised^6^. Although *matK* primers had been the subject of several studies^41^,^42^,^43^, obtaining *matK* fragments from ferns and mosses continues to be a challenge. At the beginning of our work, hardly any *ycf1* sequences had been deposited in GenBank; consequently, we had to generate *ycf1* sequences to facilitate primer design. The amplification successes of the *ycf1* primers used in this study were quite satisfactory: 98.17% for angiosperms, 90.91% for gymnosperms, 82.80% for Monilophytes, and 94.12% for Bryophytes. Amplification in seed plants would not be very difficult using the universal primers. The primer performances for Monilophytes and gymnosperms were relatively poor (Table 1) due to the significant divergence within these groups. To minimise PCR failure, taxon-specific primers were generated for focal taxa (see the electronic supplementary material, Table S3).

## Methods

### Reconfirming the variability of the *ycf1*a and *ycf1*b regions

Very few *ycf1* sequences have been deposited in GenBank. We downloaded 144 whole plastid or chloroplast genomes from GenBank (see the electronic supplementary material, Table S1). The *ycf1* sequences were extracted from two or more plastid genomes from the same genera of land plants (sorted into four groups: Bryophytes, Monilophytes, gymnosperms and angiosperms), aligned using MAFFT and manually adjusted with Se-Al 2.0 as necessary. The nucleotide diversity (π) was computed using the R package with a 600 bp sliding-window and a 50 bp step size. The averages within each group represent *ycf1* variability and the most variable regions were roughly identified.

### Primer design

All of the *ycf1* sequences deposited in GenBank were downloaded or extracted from the plastid genomes. The sequences were sorted into four groups as described above, aligned in each group using Clustal X ver. 2.0 and then adjusted using Se-Al 2.0. These sequences served as initial templates for the design of several primer pairs spanning the roughly identified regions for each group using Primer Premier 5.0 software (Premier Biosoft International, Palo Alto, CA). The successfully amplified fragments were sequenced, and together with those from GenBank, served as templates for universal primer design and hypervariable region positioning.

### Plant materials for primer design and discrimination power analyses

To test the universality of the primers, we used 368 samples of land plants, including 34 samples from 34 Bryophyte families, 93 samples from 42 Monilophyte families, 22 samples from 10 gymnosperm families, and 219 samples from 216 angiosperm families (see the electronic supplementary material, Table S2).

Two types of tests were used to assay the performance of *ycf1*a, *ycf1*b and other markers of the same genome. The first test used almost entirely woody plant species cultivated in the Beijing Botanical Garden (BBG) of the Chinese Academy of Sciences (CAS). We used 490 samples belonging to 420 species in 76 families, including 53 samples belonging to 48 species in 5 gymnosperm families and 437 samples belonging to 372 species in 71 angiosperm families (see the electronic supplementary material, Table S4). The use of BBG materials represents a common practice of using DNA barcoding for the identification of plant materials from local flora such as from large-scale community plots.

The second test used the well-sampled groups of seed plants (see the electronic supplementary material, Table S5), namely gymnosperm *Pinus* (Pinaceae); basal angiosperm Calycanthaceae; monocotyledon *Iris* (Iridaceae); Saxifragales *Paeonia* sect. *Moutan* (Paeoniaceae); rosid *Prunus* sect. *Armeniaca* (Rosaceae); *Quercus* (Fagaceae); and asterid *Panax* (Araliaceae). We attempted to include a representative from each major angiosperm group. The species of Calycanthaceae, *Paeonia* sect. *Moutan*, *Prunus* sect. *Armeniaca*, and *Panax* were completely sampled. Unfortunately, there are too many species in *Pinus*, *Iris*, and *Quercus* for full inclusion in this study.

### Experimental details

Genomic DNA was extracted from fresh or silica gel-dried leaves using the mCTAB method^44^. The 25-μL PCR reactions contained 1 × PCR buffer (with Mg^2+^), 0.25 mmol/L of each dNTP, 0.25 μmol/L of each primer, 1.25 U Taq polymerase, and 20–30 ng DNA. The PCR program consisted of 4 min at 94°C, 34 cycles of 30 s at 94°C, 40 s at 52°C, and 1 min at 72°C, followed by 10 min at 72°C. The PCR products were examined by electrophoresis on a 1% agarose gel containing ethidium bromide and visualised using an ultraviolet transilluminator. Both strands were sequenced on an ABI 3730xl DNA analyzer (Applied Biosystems, Foster City, U.S.A.) according to the manufacturer's protocols.

The primers used in this study were matK472F and matK1248R^43^ for angiosperm *matK*, Gym_F1A and Gym_R1A^42^ for gymnosperm *matK*, rbcLbF and rbcLbR for *rbcL*b^6^, and cp001F and cp001R^45^ for *trnH-psbA*.

### Data analyses

The sequences were edited and assembled using a Sequencer 4.7 (Gene Codes, Ann Arbor, MI, USA), aligned using Clustal X ver2.0 and manually adjusted with Se-Al 2.0 as necessary.

BLAST, distance and tree-building methods were used to evaluate the performance of *ycf1*, *matK*, *rbcL* and *trnH-psbA*. BLAST (version 2.2.17) was used for the BBG sequences. The entire data set was used as a reference database and each sequence was used as a query. Only hits having E values < 1 × 10^−5^ were considered. If the hits with the highest scores included sequences from more than one species, the identification was considered to be a failure, otherwise it was considered successful following China Plant BOL Groups^20^.

In addition to Blast, distance and tree-building methods were used on the seven well-sampled plant groups. The Kimura 2-parameter (K2P) distances were calculated using MEGA 5.0. We considered discrimination to be successful if the minimum uncorrected interspecific K2P distance of focal species was greater than their maximum intraspecific distance. When using the tree-building method, neighbour joining (NJ) and unweighted pair group method with arithmetic mean (UPGMA) dendrograms based on K2P distances were constructed using PAUP 4.0. Species were considered to have been discriminated from one another if all of the individuals of a species formed a single and exclusive clade.

To assess the effects of multiple gene regions on the resolution of species, we compared the resolution of species as a cumulative percentage for each combination of gene regions for both the BBG samples and the seven representative plant groups.

## Author Contributions

S.Z. and W.D. designed the study; W.D. and C.L. analyzed the data; W.D. and C.X. performed the laboratory work; S.Z., W.D., C.X., J.S., Y.Z., S.S., T.C. and J.G. collected materials; W.D., C.X. and S.Z. wrote the manuscript. All authors read and approve the final manuscript.

## Supplementary Material

Supplementary Information Supplementary Information

## Figures and Tables

**Table 1:** Universal primers for amplifying *ycf1* from Bryophytes, Monilophytes, gymnosperms and angiosperms as a DNA barcode

Plant group	Name	Sequence (5′–3′)	Tm (°C)	PCR success (%)
Bryophytes	ycf1mF	AGTTAAACGTATTATTTATCGAAC	47.2	94.12
	ycf1mR	AGATTTTTCCAAGAGCGTTCTAGTA	54.3	
Monilophytes	ycf1fF	TCTCAAGCTTRTCTCTATGACRRTATWTGG	56.4	82.80
	ycf1fR1	ATCTGTAACTAGCCAYGGCATYAAATCA	58.3	
	ycf1fR2	AGTTTCRCTTCARATTTCCATTTCCA	55.7	
Gymnosperm	ycf1gF	TGAAAGCTCTAAGCAATGGATCYCC	58.1	90.91
	ycf1gR	ATACGACCAATATTTTTRGCTATTAT	49.8	
Angiosperm	ycf1bF	TCTCGACGAAAATCAGATTGTTGTGAAT	57.0	98.17
	ycf1bR	ATACATGTCAAAGTGATGGAAAA	51.1	
