# Supplementary material for: ycf1, the most promising plastid DNA barcode of land plants
Source: Sci Rep. 2015 Feb 12;5:8348. doi: 10.1038/srep08348 (PMC4325322; doi:10.1038/srep08348)
Supplement: Supplementary Information [file srep08348-s1.pdf]

**Supplementary Information**

# *ycf1*, the most promising plastid DNA barcode of land plants

Wenpan Dong[1], Chao Xu[1], Changhao Li[1, 2], Jiahui Sun[1, 2], Yunjuan Zuo[1], Shuo Shi[1], Tao Cheng[1], Junjie Guo[3], Shiliang Zhou[1]*

1. State Key Laboratory of Systematic and Evolutionary Botany, Institute of Botany, Chinese Academy of Sciences, Beijing 100093, China

2. University of Chinese Academy of Sciences, Beijing 100049, China

3. Research Institute of Tropical Forestry, the Chinese Academy of Forestry, Gongdong, Guangzhou 510520, China

*Correspondence: Shiliang Zhou, Email: slzhou@ibcas.ac.cn; Fax: 8610 62590843

Figure S1. Performances of different combinations of *matK*, *rbcL*b, *trnH-psbA* and *ycf1*b in resolving species in seven well-sampled plant groups representing gymnosperms, basal angiosperms, monocots, Saxifragales, rosids, and asterids.

Table S1. The 144 plastid genomes used for *ycf1* primer design.

Table S2. The 368 samples used to test primer universality.

Table S3. Taxon-specific *ycf1* primers in the event of failure of the universal primers listed in Table 1.

Table S4. The 490 samples collected from the Beijing Botanical Garden, CAS for candidate barcode resolution testing.

Table S5. Samples of seven groups representing seed plants for candidate barcode resolution testing. The sequences of taxa without vouchers were downloaded

from GenBank.

Table S6. Nucleotide diversity of *ycf1*b together with three other markers and their species-discriminating powers in *Pinus* (Pinaceae).

Table S7. Nucleotide diversity of *ycf1*b together with three other markers and their species-discriminating powers in Calycanthaceae.

Table S8. Nucleotide diversity of *ycf1*b together with three other markers and their species-discriminating powers in *Iris* (Iridaceae).

Table S9. Nucleotide diversity of *ycf1*b together with three other markers and their species-discriminating powers in *Paeonia* sect. *Moutan* (Paeoniaceae).

Table S10. Nucleotide diversity of *ycf1*b together with three other markers and their species-discriminating powers in *Prunus* sect. *Armeniaca* (Rosaceae).

Table S11. Nucleotide diversity of *ycf1*b together with three other markers and their species-discriminating powers in *Quercus* (Fagaceae).

Table S12. Nucleotide diversity of *ycf1*b together with three other markers and their species-discriminating powers in *Panax* (Araliaceae).

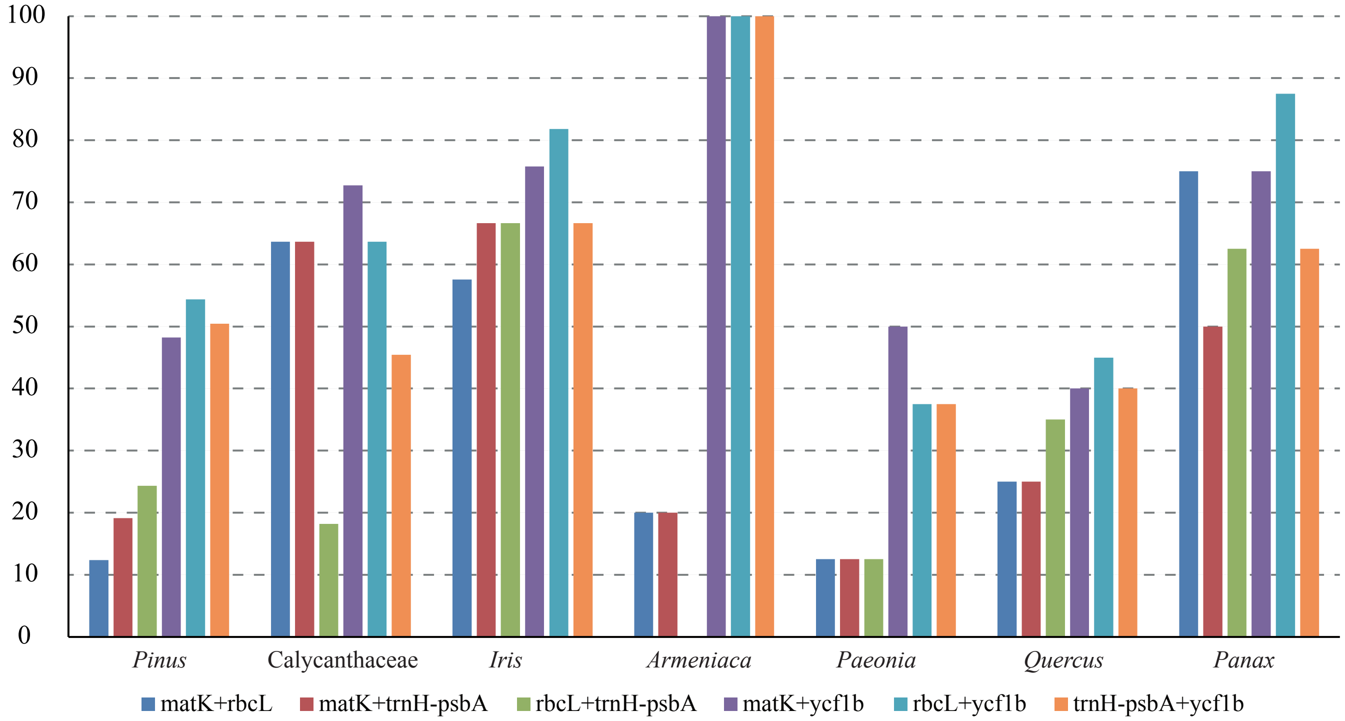
100
90
80
70
60
50
40
30
20
10
0
Pinus
Calycanthaceae
Iris
Armeniaca
Paeonia
Quercus
Panax
matK+rbcL
matK+trnH-psbA
rbcL+trnH-psbA
matK+ycf1b
rbcL+ycf1b
trnH-psbA+ycf1b

**Table S1. The 144 plastid genomes used for *ycf1* primer design.**

| Groups | family | species | Accession Number |
|---|---|---|---|
| Bryophytes | Funariaceae | Physcomitrella patens subsp. patens | NC_005087 |
| Bryophytes | Marchantiaceae | Marchantia polymorpha | NC_001319 |
| Bryophytes | Pottiaceae | Syntrichia ruralis | NC_012052 |
| Bryophytes | Ptilidiaceae | Ptilidium pulcherrimum | NC_015402 |
| | | | |
| Monilophytes | Equisetaceae | Equisetum arvense | NC_014699 |
| Monilophytes | Equisetaceae | Equisetum hyemale | NC_020146 |
| Monilophytes | Ophioglossaceae | Mankyua chejuensis | NC_017006 |
| Monilophytes | Ophioglossaceae | Ophioglossum californicum | NC_020147 |
| | | | |
| Gymnosperms | Cephalotaxaceae | Cephalotaxus oliveri | KC136217 |
| Gymnosperms | Cephalotaxaceae | Cephalotaxus wilsoniana | AP012265 |
| Gymnosperms | Cycadaceae | Cycas revoluta | JN867588 |
| Gymnosperms | Cycadaceae | Cycas taitungensis | AP009339 |
| Gymnosperms | Pinaceae | Picea abies | NC_021456 |
| Gymnosperms | Pinaceae | Picea morrisonicola | NC_016069 |
| Gymnosperms | Pinaceae | Picea sitchensis | NC_011152 |
| Gymnosperms | Taxodiaceae | Taiwania cryptomerioides | AP012266 |
| Gymnosperms | Taxodiaceae | Taiwania flousiana | KC427274 |
| | | | |
| Angiosperms | Acoraceae | Acorus americanus | NC_010093 |
| Angiosperms | Acoraceae | Acorus calamus | NC_007407 |
| Angiosperms | Apocynaceae | Asclepias albicans | JN710458 |
| Angiosperms | Apocynaceae | Asclepias albicans | JN710457 |
| Angiosperms | Apocynaceae | Asclepias albicans x Asclepias subulata | JN710470 |
| Angiosperms | Apocynaceae | Asclepias coulteri | JN710459 |
| Angiosperms | Apocynaceae | Asclepias cutleri | JN710461 |
| Angiosperms | Apocynaceae | Asclepias cutleri | JN710460 |
| Angiosperms | Apocynaceae | Asclepias leptopus | JN710462 |
| Angiosperms | Apocynaceae | Asclepias macrotis | JN710464 |
| Angiosperms | Apocynaceae | Asclepias macrotis | JN710463 |
| Angiosperms | Apocynaceae | Asclepias masonii | JN710465 |
| Angiosperms | Apocynaceae | Asclepias nivea | KF539844 |
| Angiosperms | Apocynaceae | Asclepias subulata | JN710469 |
| Angiosperms | Apocynaceae | Asclepias subulata | JN710468 |
| Angiosperms | Apocynaceae | Asclepias subulata | JN710467 |
| Angiosperms | Apocynaceae | Asclepias subulata | JN710466 |
| Angiosperms | Apocynaceae | Asclepias syriaca | KF386166 |
| Angiosperms | Apocynaceae | Asclepias syriaca | JF433943 |
| Angiosperms | Asteraceae | Chrysanthemum indicum | JN867589 |
| Angiosperms | Asteraceae | Chrysanthemum indicum | JN867592 |
| Angiosperms | Brassicaceae | Aethionema cordifolium | AP009336 |
| Angiosperms | Brassicaceae | Aethionema grandiflorum | AP009367 |
| Angiosperms | Brassicaceae | Brassica napus | NC_016734 |
| Angiosperms | Brassicaceae | Brassica rapa subsp. pekinensis | DQ231548 |
| Angiosperms | Brassicaceae | Pachycladon cheesemanii | NC_021102 |

| | | | |
|---|---|---|---|
| Angiosperms | Brassicaceae | Pachycladon enysii | NC_018565 |
| Angiosperms | Calycanthaceae | Calycanthus chinensis | This study |
| Angiosperms | Calycanthaceae | Calycanthus floridus var. glaucus | AJ428413 |
| Angiosperms | Calycanthaceae | Chimonanthus nitens | This study |
| Angiosperms | Calycanthaceae | Chimonanthus praecox | This study |
| Angiosperms | Cucurbitaceae | Cucumis melo subsp. melo | JF412791 |
| Angiosperms | Cucurbitaceae | Cucumis sativus | DQ865975 |
| Angiosperms | Cucurbitaceae | Cucumis sativus | DQ865976 |
| Angiosperms | Fabaceae | Glycine canescens | KC893635 |
| Angiosperms | Fabaceae | Glycine cyrtoloba | KC893632 |
| Angiosperms | Fabaceae | Glycine dolichocarpa | KC893636 |
| Angiosperms | Fabaceae | Glycine falcata | KC893637 |
| Angiosperms | Fabaceae | Glycine max | DQ317523 |
| Angiosperms | Fabaceae | Glycine stenophita | KC893634 |
| Angiosperms | Fabaceae | Glycine syndetika | KC893638 |
| Angiosperms | Fabaceae | Glycine tomentella | KC893633 |
| Angiosperms | Fabaceae | Glycine soja | KC779227 |
| Angiosperms | Fabaceae | Vigna angularis | AP012598 |
| Angiosperms | Fabaceae | Vigna radiata | GQ893027 |
| Angiosperms | Fabaceae | Vigna unguiculata | JQ755301 |
| Angiosperms | Fabaceae | Vigna unguiculata | JN676191 |
| Angiosperms | Magnoliaceae | Magnolia denudata | JN227740 |
| Angiosperms | Magnoliaceae | Magnolia denudata | JN867577 |
| Angiosperms | Magnoliaceae | Magnolia grandiflora | JN867584 |
| Angiosperms | Magnoliaceae | Magnolia grandiflora | JN867587 |
| Angiosperms | Magnoliaceae | Magnolia kwangsiensis | HM775382 |
| Angiosperms | Magnoliaceae | Magnolia officinalis | JN867579 |
| Angiosperms | Magnoliaceae | Magnolia officinalis subsp. biloba | JN867580 |
| Angiosperms | Magnoliaceae | Magnolia officinalis subsp. biloba | JN867581 |
| Angiosperms | Magnoliaceae | Magnolia officinalis subsp. biloba | JN867582 |
| Angiosperms | Malvaceae | Gossypium anomalum | JF317351 |
| Angiosperms | Malvaceae | Gossypium arboreum | HQ325740 |
| Angiosperms | Malvaceae | Gossypium areysianum | JN019795 |
| Angiosperms | Malvaceae | Gossypium barbadense | HQ901200 |
| Angiosperms | Malvaceae | Gossypium barbadense | HQ901198 |
| Angiosperms | Malvaceae | Gossypium barbadense | HQ901199 |
| Angiosperms | Malvaceae | Gossypium barbadense | AP009123 |
| Angiosperms | Malvaceae | Gossypium bickii | JF317352 |
| Angiosperms | Malvaceae | Gossypium capitis-viridis | JN019794 |
| Angiosperms | Malvaceae | Gossypium darwinii | HQ325741 |
| Angiosperms | Malvaceae | Gossypium gossypioides | HQ901195 |
| Angiosperms | Malvaceae | Gossypium herbaceum | JF317353 |
| Angiosperms | Malvaceae | Gossypium herbaceum var. africanum | HQ325742 |
| Angiosperms | Malvaceae | Gossypium hirsutum | HQ901196 |
| Angiosperms | Malvaceae | Gossypium hirsutum | DQ345959 |
| Angiosperms | Malvaceae | Gossypium hirsutum | HQ901197 |
| Angiosperms | Malvaceae | Gossypium incanum | JN019792 |
| Angiosperms | Malvaceae | Gossypium longicalyx | JF317354 |

| | | | |
|---|---|---|---|
| Angiosperms | Malvaceae | Gossypium mustelinum | HQ325743 |
| Angiosperms | Malvaceae | Gossypium raimondii | HQ325744 |
| Angiosperms | Malvaceae | Gossypium robinsonii | JN019791 |
| Angiosperms | Malvaceae | Gossypium somalense | JN019793 |
| Angiosperms | Malvaceae | Gossypium stocksii | JF317355 |
| Angiosperms | Malvaceae | Gossypium sturtianum | JF317356 |
| Angiosperms | Malvaceae | Gossypium thurberi | GU907100 |
| Angiosperms | Malvaceae | Gossypium tomentosum | HQ325745 |
| Angiosperms | Myrtaceae | Angophora costata | NC_022412 |
| Angiosperms | Myrtaceae | Angophora floribunda | NC_022411 |
| Angiosperms | Myrtaceae | Corymbia eximia | NC_022409 |
| Angiosperms | Myrtaceae | Corymbia gummifera | NC_022407 |
| Angiosperms | Myrtaceae | Corymbia maculata | NC_022408 |
| Angiosperms | Myrtaceae | Corymbia tessellaris | NC_022410 |
| Angiosperms | Myrtaceae | Eucalyptus globulus subsp. globulus | AY780259 |
| Angiosperms | Myrtaceae | Eucalyptus grandis | HM347959 |
| Angiosperms | Nelumbonaceae | Nelumbo lutea | JQ336992 |
| Angiosperms | Nelumbonaceae | Nelumbo nucifera | JQ336993 |
| Angiosperms | Oleaceae | Olea europaea | GU228899.2 |
| Angiosperms | Oleaceae | Olea europaea | GU931818 |
| Angiosperms | Oleaceae | Olea europaea subsp. cuspidata | FN996943.2 |
| Angiosperms | Oleaceae | Olea europaea subsp. cuspidata | FN996944 |
| Angiosperms | Oleaceae | Olea europaea subsp. cuspidata | FN650747.2 |
| Angiosperms | Oleaceae | Olea europaea subsp. Europaea | FN996972 |
| Angiosperms | Oleaceae | Olea europaea subsp. europaea | HF558645 |
| Angiosperms | Oleaceae | Olea europaea subsp. europaea | FN997651 |
| Angiosperms | Oleaceae | Olea europaea subsp. europaea | FN997650.2 |
| Angiosperms | Oleaceae | Olea europaea subsp. maroccana | FN998900.2 |
| Angiosperms | Oleaceae | Olea woodiana subsp. woodiana | FN998901 |
| Angiosperms | Orchidaceae | Cymbidium aloifolium | KC876122 |
| Angiosperms | Orchidaceae | Cymbidium mannii | KC876129 |
| Angiosperms | Orchidaceae | Cymbidium mannii | KC876126 |
| Angiosperms | Orchidaceae | Cymbidium sinense | KC876123 |
| Angiosperms | Orchidaceae | Cymbidium tortisepalum | KC876124 |
| Angiosperms | Orchidaceae | Cymbidium tortisepalum | KC876128 |
| Angiosperms | Orchidaceae | Cymbidium tortisepalum | KC876125 |
| Angiosperms | Orchidaceae | Cymbidium tracyanum | KC876127 |
| Angiosperms | Salicaceae | Populus alba | NC_008235 |
| Angiosperms | Salicaceae | Populus trichocarpa | NC_009143 |
| Angiosperms | Solanaceae | Nicotiana sylvestris | NC_007500 |
| Angiosperms | Solanaceae | Nicotiana tabacum | NC_001879 |
| Angiosperms | Solanaceae | Nicotiana tomentosiformis | NC_007602 |
| Angiosperms | Solanaceae | Solanum bulbocastanum | DQ347958 |
| Angiosperms | Solanaceae | Solanum lycopersicum | AM087200.3 |
| Angiosperms | Solanaceae | Solanum tuberosum | DQ231562 |
| Angiosperms | Theaceae | Camellia cuspidata | KF156833 |
| Angiosperms | Theaceae | Camellia danzaiensis | KF156834 |
| Angiosperms | Theaceae | Camellia impressinervis | KF156835 |

| | | | |
|---|---|---|---|
| Angiosperms | Theaceae | Camellia pitardii | KF156837 |
| Angiosperms | Theaceae | Camellia sinensis | KC143082 |
| Angiosperms | Theaceae | Camellia taliensis | KF156839 |
| Angiosperms | Theaceae | Camellia taliensis | KF156836 |
| Angiosperms | Theaceae | Camellia yunnanensis | KF156838 |

**Table S2. The 368 samples used to test primer universality.**

| | Group | Family | Species | Locality | Voucher | PCR success |
|---|---|---|---|---|---|---|
| 1 | Bryophytes | Amblystegiaceae | *Calliergon* sp. | Mt.Emeishan, Sichuan, China | H807 | yes |
| 2 | Bryophytes | Bartramiaceae | *Philonotis falcata* | Bomi, Tibet, China | J05625 | yes |
| 3 | Bryophytes | Brachytheciaceae | *Eurhynchium arbuscula* | Luding, Sichuang, China | J02022 | no |
| 4 | Bryophytes | Bryaceae | *Bryum caespiticium* | Luding, Sichuang, China | J02232 | no |
| 5 | Bryophytes | Calymperaceae | *Syrrhopodon* sp. | Ledong, Hainan. China | W24382 | yes |
| 6 | Bryophytes | Climaciaceae | *Climacium dendroides* | Kangding, Sichuan, China | J02421 | yes |
| 7 | Bryophytes | Cryphaeaceae | *Pilotrichopisis dentata* | Maerkang, Sichuan, China | J03005 | yes |
| 8 | Bryophytes | Dicranaceae | *Campylopus* sp. | Leiwuqi, Tibet, China | J08073 | yes |
| 9 | Bryophytes | Ditrichaceae | *Ceratodon purpureus* | Leiwuqi, Tibet, China | J07953 | yes |
| 10 | Bryophytes | Entodontaceae | *Entodon concinnus* | Kangding, Sichuan, China | J02385 | yes |
| 11 | Bryophytes | Fabroniaceae | *Schwetschkeopisis* | Maerkang, Sichuan, China | J02983 | yes |
| 12 | Bryophytes | Fissidentales | *Fissdens cristatus* | Luding, Sichuang, China | J01755 | yes |
| 13 | Bryophytes | Fontinalaceae | *Fontinalis antipyretica* | Bruce Allen | B24287 | yes |
| 14 | Bryophytes | Hookeriaceae | *Hookeriopsis utacamundiama* | Mt.Emeishan, Sichuan, China | P537 | yes |
| 15 | Bryophytes | Hylocomiaceae | *Hylocomiastrum umbratum* | Wenchuan, Sichuan, China | J06618 | yes |
| 16 | Bryophytes | Hypnaceae | *Hypnnum piumaeforme* | Mangkang, Tibet, China | J05118 | yes |
| 17 | Bryophytes | Hypopterygiaceae | *Cyathophorella hookeriana* | Luding, Sichuang, China | J01934 | yes |
| 18 | Bryophytes | Lembophyllaceae | *Isothecium myurum* | Nanyue, Hainan, China | L96420 | yes |
| 19 | Bryophytes | Leskeaceae | *Pseudleskeopsis zippelii* | Bomi, Tibet, China | J05642 | yes |
| 20 | Bryophytes | Leucodontaceae | *Leucodon secundus* | Bomi, Tibet, China | J05729 | yes |
| 21 | Bryophytes | Meteoriaceae | *Floribundaria setschwanica* | Maerkang, Sichuan, China | J03259 | yes |
| 22 | Bryophytes | Mniaceae | *Plagiomnium japonicum* | Luding, Sichuang, China | J01933 | yes |
| 23 | Bryophytes | Myuriaceae | *Oedicladium fragile* | Changdu, Hainan, China | W45245 | yes |
| 24 | Bryophytes | Neckeraceae | *Neckera pennata* | Linzhi, Tibet, China | J05784 | yes |
| 25 | Bryophytes | Orthotrichaceae | *Orthotrihcum alpestre* | Mangkang, Tibet, China | J05082 | yes |
| 26 | Bryophytes | Pogotrichaceae | *Pogonatum* sp. | Jiangda, Tibet, China | J07643 | yes |
| 27 | Bryophytes | Pottiaceae | *Tortella* sp. | Changdu, Tibet, China | J08103 | yes |

| | | | | | | | |
|---|---|---|---|---|---|---|---|
| 28 | Bryophytes | Pterobryaceae | *Meteoriella soluta* | Dao zhen, Guizhou, China | H364 | yes | |
| 29 | Bryophytes | Ptychomitriaceae | *Ptychomitrium formosicum* | Xiangcheng, Sichuang, China | J07207 | yes | |
| 30 | Bryophytes | Racopiaceae | *Racopilum* sp. | Mt.Emeishan, Sichuan, China | H1289 | yes | |
| 31 | Bryophytes | Splachnaceae | *Tayloria* sp. | Leiwuqi, Tibet, China | J07973 | yes | |
| 32 | Bryophytes | Theliaceae | *Fauriella* sp. | Mt.Emeishan, Sichuan, China | H1009 | yes | |
| 33 | Bryophytes | Thuidiaceae | *Abietinella abietina* | Kangding, Sichuan, China | J02321 | yes | |
| 34 | Bryophytes | Trachypodaceae | *Trachypus bicolor* | Luding, Sichuang, China | J01988 | yes | |
| | PCR success | | | | | | 94.12% |
| 1 | Monilophytes | Acrostichaceae | *Acrostichum* sp. | Fairylake Botanical Garden, Shenzhen, Guangdong, China | BOP026932 | | no |
| 2 | Monilophytes | Adiantaceae | *Adiantum capillus-veneris* | Gaoshu, Pingtung, Taiwan, China | BOP017860 | yes | |
| 3 | Monilophytes | Adiantaceae | *Coniogramme intermedia* | Mt.Jigongshan, Xinyang, Henan, China | BOP017018 | yes | |
| 4 | Monilophytes | Angiopteridaceae | *Angiopteris* sp. | Fairylake Botanical Garden, Shenzhen, Guangdong, China | BOP026934 | | no |
| 5 | Monilophytes | Antrophyllaceae | *Antrophyllum* sp. | Fairylake Botanical Garden, Shenzhen, Guangdong, China | BOP026951 | yes | |
| 6 | Monilophytes | Aspidiaceae | *Hypodematium crenatum* | Gaoshu, Pingtung, Taiwan, China | BOP017861 | yes | |
| 7 | Monilophytes | Aspidiaceae | *Tectaria decurrens* | Gaoshu, Pingtung, Taiwan, China | BOP017862 | yes | |
| 8 | Monilophytes | Aspleniaceae | *Asplenium australasicum* | Gaoshu, Pingtung, Taiwan, China | BOP017863 | yes | |
| 9 | Monilophytes | Aspleniaceae | *Boniniella cadiplylla* | Fairylake Botanical Garden, Shenzhen, Guangdong, China | BOP026900 | yes | |
| 10 | Monilophytes | Aspleniaceae | *Neottopteris nidus* | Fairylake Botanical Garden, Shenzhen, Guangdong, China | BOP026901 | yes | |
| 11 | Monilophytes | Aspleniaceae | *Sinephropteris delavayi* | Fairylake Botanical Garden, Shenzhen, Guangdong, China | BOP026902 | yes | |
| 12 | Monilophytes | Athyriaceae | *Athyrium* sp. | Fairylake Botanical Garden, Shenzhen, Guangdong, China | BOP026964 | yes | |
| 13 | Monilophytes | Athyriaceae | *Cornopteris decurrenti-alata* | Fairylake Botanical Garden, Shenzhen, Guangdong, China | BOP026961 | yes | |
| 14 | Monilophytes | Athyriaceae | *Deparia lancea* | Gaoshu, Pingtung, Taiwan, China | BOP017864 | yes | |
| 15 | Monilophytes | Athyriaceae | *Diplazium subsinuatum* | Fairylake Botanical Garden, Shenzhen, Guangdong, China | BOP026960 | yes | |
| 16 | Monilophytes | Athyriaceae | *Dryoathyrium okuboanum* | Fairylake Botanical Garden, Shenzhen, Guangdong, China | BOP026958 | yes | |
| 17 | Monilophytes | Athyriaceae | *Pseudocystopteris atkinsonii* | Mt.Jigongshan, Xinyang, Henan, China | BOP017168 | yes | |
| 18 | Monilophytes | Athyriaceae | *Rhachidosorus mesosorus* | Fairylake Botanical Garden, Shenzhen, Guangdong, China | BOP026929 | yes | |
| 19 | Monilophytes | Blechnaceae | *Blechnum orientale* | Fairylake Botanical Garden, Shenzhen, Guangdong, China | BOP026905 | yes | |
| 20 | Monilophytes | Blechnaceae | *Brainea insignis* | Fairylake Botanical Garden, Shenzhen, Guangdong, China | BOP026973 | yes | |
| 21 | Monilophytes | Blechnaceae | *Woodwardia unigemmata* | Fairylake Botanical Garden, Shenzhen, Guangdong, China | BOP026909 | yes | |
| 22 | Monilophytes | Bolbitidtiaceae | *Egenolfia fengiana* | Fairylake Botanical Garden, Shenzhen, Guangdong, China | BOP026888 | yes | |
| 23 | Monilophytes | Cyatheaceae | *Alsophila spinulosa* | Beijing Botanical Garden, CAS, Beijing, China | BOP017696 | yes | |

| | | | | | | | |
|---|---|---|---|---|---|---|---|
| 24 | Monilophytes | Cyatheaceae | *Cyathea lepifera* | Gaoshu, Pingtung, Taiwan, China | BOP017866 | yes | |
| 25 | Monilophytes | Cyatheaceae | *Gymnosphaera khasyana* | Fairylake Botanical Garden, Shenzhen, Guangdong, China | BOP026957 | | no |
| 26 | Monilophytes | Cyatheaceae | *Sphaeropteris lepifera* | Fairylake Botanical Garden, Shenzhen, Guangdong, China | BOP026954 | yes | |
| 27 | Monilophytes | Cystopteridaceae | *Cystopteris pellucida* | Mt.Jigongshan, Xinyang, Henan, China | BOP017203 | | no |
| 28 | Monilophytes | Davalliaceae | *Davallia denticulata* | Fairylake Botanical Garden, Shenzhen, Guangdong, China | BOP026860 | yes | |
| 29 | Monilophytes | Davalliaceae | *Humata repens* | Fairylake Botanical Garden, Shenzhen, Guangdong, China | BOP026856 | **yes** | |
| 30 | Monilophytes | Dennstaedtiaceae | *Dennstaedtia wilfordii* | Mt.Jigongshan, Xinyang, Henan, China | BOP017010 | yes | |
| 31 | Monilophytes | Dennstaedtiaceae | *Microlepia hancei* | Fairylake Botanical Garden, Shenzhen, Guangdong, China | BOP026943 | yes | |
| 32 | Monilophytes | Dicksoniaceae | *Cibotium taiwanense* | Gaoshu, Pingtung, Taiwan, China | BOP017869 | | no |
| 33 | Monilophytes | Drynariaceae | *Drynaria roosii* | Beijing Botanical Garden, CAS, Beijing, China | BOP017692 | yes | |
| 34 | Monilophytes | Dryopteridaceae | *Arachniodes chinensis* | Mt.Jigongshan, Xinyang, Henan, China | BOP017021 | yes | |
| 35 | Monilophytes | Dryopteridaceae | *Cyrtognellum caducum* | Fairylake Botanical Garden, Shenzhen, Guangdong, China | BOP026874 | yes | |
| 36 | Monilophytes | Dryopteridaceae | *Cyrtomidictyum lepidocaulon* | Fairylake Botanical Garden, Shenzhen, Guangdong, China | BOP026875 | yes | |
| 37 | Monilophytes | Dryopteridaceae | *Cyrtomium caryotideum* | Beijing Botanical Garden, CAS, Beijing, China | BOP017698 | yes | |
| 38 | Monilophytes | Dryopteridaceae | *Cyrtomium fortunei* | Mt.Jigongshan, Xinyang, Henan, China | BOP017019 | yes | |
| 39 | Monilophytes | Dryopteridaceae | *Dryopteris labordei* | Gaoshu, Pingtung, Taiwan, China | BOP017870 | yes | |
| 40 | Monilophytes | Dryopteridaceae | *Phanerophlebiopsis blinii* | Fairylake Botanical Garden, Shenzhen, Guangdong, China | BOP026865 | yes | |
| 41 | Monilophytes | Dryopteridaceae | *Polystichum excelcior* | Fairylake Botanical Garden, Shenzhen, Guangdong, China | BOP026871 | yes | |
| 42 | Monilophytes | Elaphoglossaceae | *Elaphoglossum conforme* | Beijing Botanical Garden, CAS, Beijing, China | BOP017714 | | no |
| 43 | Monilophytes | Equisetaceae | *Equisetum ramosissimum* | Gaoshu, Pingtung, Taiwan, China | BOP017872 | | no |
| 44 | Monilophytes | Gleicheniaceae | *Dicranopteris linearis* | Gaoshu, Pingtung, Taiwan, China | BOP017873 | yes | |
| 45 | Monilophytes | Hemionitidaceae | *Hemionitis arifolia* | Fairylake Botanical Garden, Shenzhen, Guangdong, China | BOP026945 | yes | |
| 46 | Monilophytes | Hemionitidaceae | *Pityrogramma calomelanos* | Fairylake Botanical Garden, Shenzhen, Guangdong, China | BOP026950 | yes | |
| 47 | Monilophytes | Hymenophyllacea | *Trichomanes birmanica* | Fairylake Botanical Garden, Shenzhen, Guangdong, China | BOP026903 | no | |
| 48 | Monilophytes | Lindsaeaceae | *Lindsaea ensifoila* | Fairylake Botanical Garden, Shenzhen, Guangdong, China | BOP026975 | no | |
| 49 | Monilophytes | Lomariopsidaceae | *Bolbitis heteroclita* | Gaoshu, Pingtung, Taiwan, China | BOP017874 | yes | |
| 50 | Monilophytes | Lomariopsidaceae | *Lomariopsis spectabilis* | Gaoshu, Pingtung, Taiwan, China | BOP017875 | yes | |
| 51 | Monilophytes | Lycopodiaceae | *Lycopodium salvinioides* | Gaoshu, Pingtung, Taiwan, China | BOP017876 | | no |
| 52 | Monilophytes | Lygodiaceae | *Lygodium conforme* | Fairylake Botanical Garden, Shenzhen, Guangdong, China | BOP026935 | | no |
| 53 | Monilophytes | Marattiaceae | *Marattia pellucida* | Gaoshu, Pingtung, Taiwan, China | BOP017877 | | no |
| 54 | Monilophytes | Marsileaceae | *Marsilea crenata* | Gaoshu, Pingtung, Taiwan, China | BOP017878 | | no |
| 55 | Monilophytes | Nephrolepidaceae | *Nephrolepis hirsutula* | Fairylake Botanical Garden, Shenzhen, Guangdong, China | BOP026939 | | no |

| | | | | | | | |
|---|---|---|---|---|---|---|---|
| 56 | Monilophytes | Oleandraceae | *Oleandra wallichii* | Gaoshu, Pingtung, Taiwan, China | BOP017880 | yes | |
| 57 | Monilophytes | Onocleaceae | *Matteuccia struthiopteris* | Beijing Botanical Garden, CAS, Beijing, China | BOP017708 | yes | |
| 58 | Monilophytes | Ophioglossaceae | *Ophioderma pendula* | Gaoshu, Pingtung, Taiwan, China | BOP017881 | | no |
| 59 | Monilophytes | Osmundaceae | *Osmunda mildei* | Fairylake Botanical Garden, Shenzhen, Guangdong, China | BOP026936 | yes | |
| 60 | Monilophytes | Platyceriaceae | *Platycerium wallichii* | Fairylake Botanical Garden, Shenzhen, Guangdong, China | BOP026928 | | no |
| 61 | Monilophytes | Polypodiaceae | *Colysis elliptica* | Fairylake Botanical Garden, Shenzhen, Guangdong, China | BOP026916 | yes | |
| 62 | Monilophytes | Polypodiaceae | *Colysis elliptica var. longipe* | Fairylake Botanical Garden, Shenzhen, Guangdong, China | BOP026914 | yes | |
| 63 | Monilophytes | Polypodiaceae | *Lepidogrammitis pyriformis* | Fairylake Botanical Garden, Shenzhen, Guangdong, China | BOP026918 | yes | |
| 64 | Monilophytes | Polypodiaceae | *Lepidomicrosorium buergerianum* | Fairylake Botanical Garden, Shenzhen, Guangdong, China | BOP026920 | yes | |
| 65 | Monilophytes | Polypodiaceae | *Lepisorus bicolor* | Mt.Jigongshan, Xinyang, Henan, China | BOP017207 | yes | |
| 66 | Monilophytes | Polypodiaceae | *Leptochilus cantoniensis* | Fairylake Botanical Garden, Shenzhen, Guangdong, China | BOP026919 | yes | |
| 67 | Monilophytes | Polypodiaceae | *Microsorum fortunei* | Fairylake Botanical Garden, Shenzhen, Guangdong, China | BOP026912 | yes | |
| 68 | Monilophytes | Polypodiaceae | *Neolepisrus ovatus* | Fairylake Botanical Garden, Shenzhen, Guangdong, China | BOP026917 | yes | |
| 69 | Monilophytes | Polypodiaceae | *Phymatopteris rhynchophylla* | Fairylake Botanical Garden, Shenzhen, Guangdong, China | BOP026922 | yes | |
| 70 | Monilophytes | Polypodiaceae | *Phymatosorus scolopendria* | Beijing Botanical Garden, CAS, Beijing, China | BOP017690 | yes | |
| 71 | Monilophytes | Polypodiaceae | *Polypodiodes* sp. | Fairylake Botanical Garden, Shenzhen, Guangdong, China | BOP026925 | yes | |
| 72 | Monilophytes | Polypodiaceae | *Pyrrosia drakeana* | Fairylake Botanical Garden, Shenzhen, Guangdong, China | BOP026923 | yes | |
| 73 | Monilophytes | Polypodiaceae | *Saxiglossum angustissimum* | Fairylake Botanical Garden, Shenzhen, Guangdong, China | BOP026927 | yes | |
| 74 | Monilophytes | Psilotaceae | *Psilotum nudum* | Gaoshu, Pingtung, Taiwan, China | BOP017887 | yes | |
| 75 | Monilophytes | Pteridaceae | *Pteris faurei* | Fairylake Botanical Garden, Shenzhen, Guangdong, China | BOP026946 | yes | |
| 76 | Monilophytes | Salviniaceae | *Salvinia molesta* | Gaoshu, Pingtung, Taiwan, China | BOP017889 | yes | |
| 77 | Monilophytes | Selaginellaceae | *Selaginella uncinata* | Gaoshu, Pingtung, Taiwan, China | BOP017891 | yes | |
| 78 | Monilophytes | Sinopteridaceae | *Aleuritopteris* sp. | Beijing Botanical Garden, CAS, Beijing, China | BOP017715 | yes | |
| 79 | Monilophytes | Sinopteridaceae | *Doryopteris ludens* | Fairylake Botanical Garden, Shenzhen, Guangdong, China | BOP026949 | yes | |
| 80 | Monilophytes | Sinopteridaceae | *Onychium* sp. | Fairylake Botanical Garden, Shenzhen, Guangdong, China | BOP026972 | yes | |
| 81 | Monilophytes | Tectariaceae | *Ctenitis tibetica* | Fairylake Botanical Garden, Shenzhen, Guangdong, China | BOP026877 | yes | |
| 82 | Monilophytes | Tectariaceae | *Ctenitopsis glabra* | Fairylake Botanical Garden, Shenzhen, Guangdong, China | BOP026880 | yes | |
| 83 | Monilophytes | Tectariaceae | *Pleocnemia winitii* | Fairylake Botanical Garden, Shenzhen, Guangdong, China | BOP026883 | yes | |
| 84 | Monilophytes | Tectariaceae | *Pteridrys* sp. | Fairylake Botanical Garden, Shenzhen, Guangdong, China | BOP026882 | yes | |
| 85 | Monilophytes | Tectariaceae | *Queracifilix zeylanica* | Fairylake Botanical Garden, Shenzhen, Guangdong, China | BOP026884 | yes | |
| 86 | Monilophytes | Thelypteridaceae | *Ampelopteris prolifera* | Gaoshu, Pingtung, Taiwan, China | BOP017892 | yes | |
| 87 | Monilophytes | Thelypteridaceae | *Cyclogramma emeiense* | Fairylake Botanical Garden, Shenzhen, Guangdong, China | BOP026891 | yes | |

| | | | | | | |
|---|---|---|---|---|---|---|
| 88 | Monilophytes | Thelypteridaceae | *Cyclosorus truncatus* | Fairylake Botanical Garden, Shenzhen, Guangdong, China | BOP026897 | yes |
| 89 | Monilophytes | Thelypteridaceae | *Dictyocline griffithii* | Fairylake Botanical Garden, Shenzhen, Guangdong, China | BOP026890 | yes |
| 90 | Monilophytes | Thelypteridaceae | *Phegopteris decurrenti-pinoata* | Fairylake Botanical Garden, Shenzhen, Guangdong, China | BOP026940 | yes |
| 91 | Monilophytes | Thelypteridaceae | *Pronephrium penangianum* | Beijing Botanical Garden, CAS, Beijing, China | BOP017694 | yes |
| 92 | Monilophytes | Thelypteridaceae | *Stegnogramma cyrtomioides* | Fairylake Botanical Garden, Shenzhen, Guangdong, China | BOP026892 | yes |
| 93 | Monilophytes | Vittariaceae | *Haplopteris* sp. | Fairylake Botanical Garden, Shenzhen, Guangdong, China | BOP026952 | yes |
| | PCR success | | | | | 82.80% |
| 1 | Gymnosperm | Araucariaceae | *Araucaria cunninghamii* | Beijing Botanical Garden, CAS, Beijing, China | BOP017780 | no |
| 2 | Gymnosperm | Cephalotaxaceae | *Cephalotaxus sinensis* | Beijing Botanical Garden, CAS, Beijing, China | BOP010413 | yes |
| 3 | Gymnosperm | Cupressaceae | *Chamaecyparis pisifera* | Beijing Botanical Garden, CAS, Beijing, China | BOP010369 | yes |
| 4 | Gymnosperm | Cupressaceae | *Platyladus orientalis* | Beijing Botanical Garden, CAS, Beijing, China | BOP010371 | yes |
| 5 | Gymnosperm | Cupressaceae | *Sabina chinensis* | Mt.Jigongshan, Xinyang, Henan, China | BOP017339 | yes |
| 6 | Gymnosperm | Cycadaceae | *Cycas revoluta* | Beijing Botanical Garden, CAS, Beijing, China | BOP017781 | yes |
| 7 | Gymnosperm | Ephedraceae | *Ephedra equisetina* | Beijing Botanical Garden, CAS, Beijing, China | BOP010404 | no |
| 8 | Gymnosperm | Ginkgoaceae | *Ginkgo biloba* | Beijing Botanical Garden, CAS, Beijing, China | BOP010383 | yes |
| 9 | Gymnosperm | Podocarpaceae | *Podocarpus macrophyllus* | Mt.Jigongshan, Xinyang, Henan, China | BOP017081 | yes |
| 10 | Gymnosperms | Taxaceae | *Amento taxus* | Beijing Botanical Garden, CAS, Beijing, China | BOP010495 | yes |
| 11 | Gymnosperm | Taxaceae | *Taxus cuspidata* | Beijing Botanical Garden, CAS, Beijing, China | BOP010401 | yes |
| 12 | Gymnosperms | Taxodiaceae | *Larix gmelinii* | Beijing Botanical Garden, CAS, Beijing, China | BOP010368 | yes |
| 13 | Gymnosperms | Taxodiaceae | *Pseudotsuga menziesii* | Beijing Botanical Garden, CAS, Beijing, China | BOP010481 | yes |
| 14 | Gymnosperms | Taxodiaceae | *Taxodium ascendens* | Mt.Jigongshan, Xinyang, Henan, China | BOP017530 | yes |
| 15 | Gymnosperm | Taxodiaceae | *Abies firma* | Beijing Botanical Garden, CAS, Beijing, China | BOP010382 | yes |
| 16 | Gymnosperm | Taxodiaceae | *Cedrus deodara* | Beijing Botanical Garden, CAS, Beijing, China | BOP010415 | yes |
| 17 | Gymnosperm | Taxodiaceae | *Cunninghamia lanceolata* | Mt.Jigongshan, Xinyang, Henan, China | BOP017093 | yes |
| 18 | Gymnosperm | Taxodiaceae | *Juniperus rigida* | Beijing Botanical Garden, CAS, Beijing, China | BOP010380 | yes |
| 19 | Gymnosperm | Taxodiaceae | *Metasequoia glyptostroboides* | Beijing Botanical Garden, CAS, Beijing, China | BOP010414 | yes |
| 20 | Gymnosperm | Taxodiaceae | *Picea crassifolia* | Beijing Botanical Garden, CAS, Beijing, China | BOP010386 | yes |
| 21 | Gymnosperm | Taxodiaceae | *Taiwania cryptomerioides* | Beijing Botanical Garden, CAS, Beijing, China | BOP017785 | yes |
| 22 | Gymnosperm | Zamiaceae | *Zamia sp.* | Beijing Botanical Garden, CAS, Beijing, China | BOP017782 | yes |
| | PCR success | | | | | 90.91% |
| 1 | Angiosperms | Acanthaceae | | Beijing Botanical Garden, CAS, Beijing, China | BOP010549 | yes |

| | | | | | | |
|---|---|---|---|---|---|---|
| 2 | Angiosperms | Aceraceae | *Acer truncatum* | Beijing Botanical Garden, CAS, Beijing, China | BOP010011 | yes |
| 3 | Angiosperms | Acoraceae | | Beijing Botanical Garden, CAS, Beijing, China | BOP010522 | yes |
| 4 | Angiosperms | Actinidiaceae | *Actinidia chinensis* | Beijing Botanical Garden, CAS, Beijing, China | BOP010142 | yes |
| 5 | Angiosperms | Adoxaceae | *Adoxa moschatellina* | Heilongjiang, China | M01 | yes |
| 6 | Angiosperms | Agavaceae | *Agave* sp. | Beijing Botanical Garden, CAS, Beijing, China | BOP010551 | yes |
| 7 | Angiosperms | Aizoaceae | | Xishuangbanna Botanical Garden, CAS,Yunnan,China | BOP016916 | yes |
| 8 | Angiosperms | Alangiaceae | *Alangium chinense* subsp. *pauciflorum* | Beijing Botanical Garden, CAS, Beijing, China | BOP010310 | yes |
| 9 | Angiosperms | Alismataceae | *Alisma orientale* | Mt. Maoershan, Heilongjiang, China | BOP017758 | yes |
| 10 | Angiosperms | Altingiaceae | | Xishuangbanna Botanical Garden, Yunnan, China | B2 | yes |
| 11 | Angiosperms | Amaranthaceae | | Beijing Botanical Garden, CAS, Beijing, China | BOP010537 | yes |
| 12 | Angiosperms | Amaryllidaceae | *Crinum asiaticum* var. *sinicum* | Beijing Botanical Garden, CAS, Beijing, China | BOP010547 | yes |
| 13 | Angiosperms | Anacardiaceae | *Rhus punjabensis* var. *sinica* | Beijing Botanical Garden, CAS, Beijing, China | BOP010074 | yes |
| 14 | Angiosperms | Ancistrocladaceae | *Ancistrocladus tectorius* | Xishuangbanna Botanical Garden, CAS,Yunnan,China | BOP016875 | yes |
| 15 | Angiosperms | Annonaceae | *Dasymaschalon* | Ledong, Hainan, China | 1630004 | yes |
| 16 | Angiosperms | Annonaceae | *Desmos chinensis* | Xishuangbanna Botanical Garden, Yunnan, China | BOP017746 | yes |
| 17 | Angiosperms | Apocynaceae | | Beijing Botanical Garden, CAS, Beijing, China | BOP010558 | yes |
| 18 | Angiosperms | Aquifoliaceae | *Ilex ficoidea* | Ledong, Hainan, China | 1630189 | yes |
| 19 | Angiosperms | Araceae | | Beijing Botanical Garden, CAS, Beijing, China | BOP010508 | yes |
| 20 | Angiosperms | Araliaceae | *Eleutherococcus sessiliflorus* | Beijing Botanical Garden, CAS, Beijing, China | BOP010019 | yes |
| 21 | Angiosperms | Aristolochiaceae | *Callicarpa americana* | Beijing Botanical Garden, CAS, Beijing, China | BOP010293 | yes |
| 22 | Angiosperms | Asclepiadaceae | *Cynanchum auriculatum* | Beijing Botanical Garden, CAS, Beijing, China | BOP010451 | yes |
| 23 | Angiosperms | Asparagaceae | *Asparagus densiflorus* | Xishuangbanna Botanical Garden, CAS,Yunnan,China | BOP016876 | yes |
| 24 | Angiosperms | Asteraceae | *Myripnois dioica* | Beijing Botanical Garden, CAS, Beijing, China | BOP010410 | yes |
| 25 | Angiosperms | Austrobaileyaceae | *Austrobaileya scandens* | Melbourne Botanic Garden, Melbourne, Australia | BOP017838 | yes |
| 26 | Angiosperms | Balsaminaceae | | Beijing Botanical Garden, CAS, Beijing, China | BOP010538 | yes |
| 27 | Angiosperms | Barringtoniaceae | *Barringtonia fusicarpa* | Xishuangbanna Botanical Garden, CAS,Yunnan,China | BOP016901 | yes |
| 28 | Angiosperms | Berberidaceae | *Berberis* sp. | Beijing Botanical Garden, CAS, Beijing, China | BOP010081 | yes |
| 29 | Angiosperms | Betulaceae | *Betula alnoides* | Beijing Botanical Garden, CAS, Beijing, China | BOP010014 | yes |
| 30 | Angiosperms | Bignoniaceae | *Campsis grandiflora* | Beijing Botanical Garden, CAS, Beijing, China | BOP010266 | yes |
| 31 | Angiosperms | Bixaceae | *Bixa orellana* | Xishuangbanna Botanical Garden, CAS,Yunnan,China | BOP016891 | yes |

| | | | | | | | |
|---|---|---|---|---|---|---|---|
| 32 | Angiosperms | Bombacaceae | *Ochroma lagopus* | Xishuangbanna Botanical Garden, Yunnan, China | BOP017744 | yes | |
| 33 | Angiosperms | Boraginaceae | | Beijing Botanical Garden, CAS, Beijing, China | BOP010530 | yes | |
| 34 | Angiosperms | Bromeliaceae | *Viresea splendens* | Beijing Botanical Garden, CAS, Beijing, China | BOP010542 | yes | |
| 35 | Angiosperms | Burseraceae | *Protium yunnanense* | Xishuangbanna Botanical Garden, CAS,Yunnan,China | BOP016908 | yes | |
| 36 | Angiosperms | Butomaceae | *Limnocharis flaca* | Xishuangbanna Botanical Garden, Yunnan, China | BOP017751 | yes | |
| 37 | Angiosperms | Buxaceae | *Buxus sinica* var. *parvifolia* | Beijing Botanical Garden, CAS, Beijing, China | BOP010025 | yes | |
| 38 | Angiosperms | Cactaceae | *Pereskia aculeata* | Xishuangbanna Botanical Garden, Yunnan, China | B11 | yes | |
| 39 | Angiosperms | Calophyllaceae | *Calophyllum inophyllum* | Xishuangbanna Botanical Garden, CAS,Yunnan,China | BOP016895 | yes | |
| 40 | Angiosperms | Calycanthaceae | *Calycanthus chinensis* | Beijing Botanical Garden, CAS, Beijing, China | BOP010108 | yes | |
| 41 | Angiosperms | Campanulaceae | *Campanula medium* | Beijing Botanical Garden, CAS, Beijing, China | BOP010286 | yes | |
| 42 | Angiosperms | Cannabaceae | *Humulus lupulus* | Beijing Botanical Garden, CAS, Beijing, China | BOP010139 | yes | |
| 43 | Angiosperms | Cannaceae | | Beijing Botanical Garden, CAS, Beijing, China | BOP010516 | yes | |
| 44 | Angiosperms | Capparaceae | *Capparis tenera* | Xishuangbanna Botanical Garden, CAS,Yunnan,China | BOP016902 | yes | |
| 45 | Angiosperms | Capparidaceae | *Stixis suaveolens* | Xishuangbanna Botanical Garden, Yunnan, China | BOP017752 | yes | |
| 46 | Angiosperms | Caprifoliaceae | *Kolkwitzia amabilis* | Beijing Botanical Garden, CAS, Beijing, China | BOP010103 | yes | |
| 47 | Angiosperms | Caryophyllaceae | | Beijing Botanical Garden, CAS, Beijing, China | BOP010511 | yes | |
| 48 | Angiosperms | Celastraceae | *Euonymus bungeanus* | Beijing Botanical Garden, CAS, Beijing, China | BOP010129 | yes | |
| 49 | Angiosperms | Cercidiphyllaceae | *Cercidiphyllum japonicum* | Beijing Botanical Garden, CAS, Beijing, China | BOP010113 | yes | |
| 50 | Angiosperms | Chenopodiaceae | *Chenopodium album* | Mt.Jigongshan, Xinyang, Henan, China | BOP017094 | yes | |
| 51 | Angiosperms | Chloranthaceae | | Harbin Botanical Garden, Harbin, Heilongjiang, China | BOP016588 | yes | |
| 52 | Angiosperms | Cistaceae | *Cistas salvifolius* | Adelaide Botanic Garden, Adelaide, Australia | BOP017810 | | no |
| 53 | Angiosperms | Cochlospermaceae | *Cochlospermum religiosum* | Xishuangbanna Botanical Garden, CAS,Yunnan,China | BOP016918 | yes | |
| 54 | Angiosperms | Combretaceae | *Combretum caffrum* | Melbourne Botanic Garden, Melbourne, Australia | BOP017856 | yes | |
| 55 | Angiosperms | Commelinaceae | | Beijing Botanical Garden, CAS, Beijing, China | BOP010509 | yes | |
| 56 | Angiosperms | Connaraceae | *Connarus yunnanensis* | Xishuangbanna Botanical Garden, CAS,Yunnan,China | BOP016884 | yes | |
| 57 | Angiosperms | Convolvulaceae | | Beijing Botanical Garden, CAS, Beijing, China | BOP010507 | yes | |
| 58 | Angiosperms | Coriariaceae | | Xishuangbanna Botanical Garden, Yunnan, China | BOP017733 | | no |
| 59 | Angiosperms | Cornaceae | *Helwingia japonica* | Xishuangbanna Botanical Garden, Yunnan, China | BOP017735 | yes | |
| 60 | Angiosperms | Corynocarpaceae | *Corynocarpus laevigatus* | Melbourne Botanic Garden, Melbourne, Australia | BOP017839 | yes | |
| 61 | Angiosperms | Crassulaceae | *Sedum sarmentosum* | Beijing Botanical Garden, CAS, Beijing, China | B4b | yes | |

| | | | | | | |
|---|---|---|---|---|---|---|
| 62 | Angiosperms | Crypteroniaceae | | Xishuangbanna Botanical Garden, CAS,Yunnan,China | BOP016910 | yes |
| 63 | Angiosperms | Cucurbitaceae | | Beijing Botanical Garden, CAS, Beijing, China | BOP010533 | yes |
| 64 | Angiosperms | Cyperaceae | *Cyperus alternifolius* | Beijing Botanical Garden, CAS, Beijing, China | BOP010552 | yes |
| 65 | Angiosperms | Daphniphyllaceae | *Daphniphyllum oldhamii* | Xishuangbanna Botanical Garden, Yunnan, China | B04c | yes |
| 66 | Angiosperms | Davidiaceae | *Davidia involucrata* var. *involucrata* | Mt.Jigongshan, Xinyang, Henan, China | BOP017086 | yes |
| 67 | Angiosperms | Davidsoniaceae | *Davidsonia pruriens* | Adelaide Botanic Garden, Adelaide, Australia | BOP017825 | yes |
| 68 | Angiosperms | Dilleniaceae | *Dillenia* sp. | Ledong, Hainan, China | 2113456 | yes |
| 69 | Angiosperms | Dioscoreaceae | *Dioscorea polystachya* | Beijing Botanical Garden, CAS, Beijing, China | BOP010540 | yes |
| 70 | Angiosperms | Dipsacaceae | | Zhongdian, Yunnan, China | BOP003131 | yes |
| 71 | Angiosperms | Dipterocarpaceae | *Dipterocarpus retusus* | Xishuangbanna Botanical Garden, CAS,Yunnan,China | BOP016898 | yes |
| 72 | Angiosperms | Ebenaceae | *Diospyros cathayensis* | Beijing Botanical Garden, CAS, Beijing, China | BOP010151 | yes |
| 73 | Angiosperms | Elaeagnaceae | *Elaeagnus multiflora* | Beijing Botanical Garden, CAS, Beijing, China | BOP010319 | yes |
| 74 | Angiosperms | Elaeocarpaceae | *Elaeocarpus hainanensis* | Xishuangbanna Botanical Garden, CAS,Yunnan,China | BOP016882 | yes |
| 75 | Angiosperms | Ericaceae | *Rhododendron noriakianum* | Tahe, Heilongjiang, China | BOP016533 | yes |
| 76 | Angiosperms | Erythroxylaceae | *Erythroxylum novogranatense* | Xishuangbanna Botanical Garden, CAS,Yunnan,China | BOP016892 | yes |
| 77 | Angiosperms | Escalloniaceae | *Polyosma cambodiana* | Ledong, Hainan, China | 1630197 | yes |
| 78 | Angiosperms | Eucommiaceae | *Eucommia ulmoides* | Beijing Botanical Garden, CAS, Beijing, China | BOP010485 | yes |
| 79 | Angiosperms | Eucryphiaceae | *Eupomatia bennettii* | Melbourne Botanic Garden, Melbourne, Australia | BOP017844 | yes |
| 80 | Angiosperms | Euphorbiaceae | *Flueggea suffruticosa* | Beijing Botanical Garden, CAS, Beijing, China | BOP010052 | yes |
| 81 | Angiosperms | Eupomatiaceae | *Eupomatia laurina* | Melbourne Botanic Garden, Melbourne, Australia | BOP017845 | yes |
| 82 | Angiosperms | Fabaceae | *Cercis chinensis* | Beijing Botanical Garden, CAS, Beijing, China | BOP010459 | yes |
| 83 | Angiosperms | Fagaceae | *Quercus aliena* | Beijing Botanical Garden, CAS, Beijing, China | BOP010439 | yes |
| 84 | Angiosperms | Findersiaceae | *Findersia australis* | Adelaide Botanic Garden, Adelaide, Australia | BOP017831 | yes |
| 85 | Angiosperms | Flacourtiaceae | *Homalium laoticum* var. *glabratum* | Ledong, Hainan, China | 1602124 | yes |
| 86 | Angiosperms | Garryaceae | *Garrya elliptica* | Melbourne Botanic Garden, Melbourne, Australia | BOP017858 | yes |
| 87 | Angiosperms | Gentianaceae | *Euptelea pleiosperma* | Beijing Botanical Garden, CAS, Beijing, China | BOP010109 | yes |
| 88 | Angiosperms | Geraniaceae | | Beijing Botanical Garden, CAS, Beijing, China | BOP010514 | yes |
| 89 | Angiosperms | Gesneriaceae | *Digitalis purpurea* | Beijing Botanical Garden, CAS, Beijing, China | BOP010528 | yes |
| 90 | Angiosperms | Goodeniaceae | *Goodenia varia* | Adelaide Botanic Garden, Adelaide, Australia | BOP017805 | yes |
| 91 | Angiosperms | Grossulariaceae | *Ribes* sp. | Beijing Botanical Garden, CAS, Beijing, China | BOP010192 | yes |

| | | | | | | |
|---|---|---|---|---|---|---|
| 92 | Angiosperms | Haemodoraceae | *Anigozanthos* sp. | Adelaide Botanic Garden, Adelaide, Australia | BOP017821 | yes |
| 93 | Angiosperms | Haloragidaceae | *Gunnera manicata* | Kunming Botanical Garden, CAS,Yunnan,China | BOP017798 | yes |
| 94 | Angiosperms | Hamamelidaceae | *Hamamelis mollis* | Beijing Botanical Garden, CAS, Beijing, China | BOP010148 | yes |
| 95 | Angiosperms | Helwingiaceae | *Helwingia chinensis* | Kunming Botanical Garden, CAS,Yunnan,China | BOP016933 | yes |
| 96 | Angiosperms | Hernandiaceae | | Xishuangbanna Botanical Garden, CAS,Yunnan,China | BOP016914 | yes |
| 97 | Angiosperms | Hippocastanaceae | *Aesculus wangii* | Beijing Botanical Garden, CAS, Beijing, China | BOP010114 | yes |
| 98 | Angiosperms | Hydrangeaceae | *Philadelphus coronarius* | Beijing Botanical Garden, CAS, Beijing, China | BOP010219 | yes |
| 99 | Angiosperms | Hydrocharitaceae | | Beijing Botanical Garden, CAS, Beijing, China | BOP010520 | yes |
| 100 | Angiosperms | Hydrophyllaceae | *Ludwigia adscendens* | Xishuangbanna Botanical Garden, CAS,Yunnan,China | BOP016919 | yes |
| 101 | Angiosperms | Hypericaceae | | Tahe, Heilongjiang, China | BOP016869 | yes |
| 102 | Angiosperms | Hypoxidaceae | *Curculigo capitulata* | Xishuangbanna Botanical Garden, Yunnan, China | BOP017747 | yes |
| 103 | Angiosperms | Icacinaceae | *Gonocaryum lobbianum* | Xishuangbanna Botanical Garden, CAS,Yunnan,China | BOP016881 | yes |
| 104 | Angiosperms | Illiciaceae | | Kunming Botanical Garden, CAS,Yunnan,China | BOP016938 | yes |
| 105 | Angiosperms | Iridaceae | *Iris lactea* var. *chinensis* | Beijing Botanical Garden, Beijing, China | BOP017765 | yes |
| 106 | Angiosperms | Iteaceae | *Itea chinensis* var. *oblonga* | Hangzhou Botanical Garden, Hangzhou, Zhejing, China | B06 | yes |
| 107 | Angiosperms | Juglandaceae | *Cyclocarya paliurus* | Beijing Botanical Garden, CAS, Beijing, China | BOP010527 | yes |
| 108 | Angiosperms | Juncaceae | | Beijing Botanical Garden, CAS, Beijing, China | BOP010519 | yes |
| 109 | Angiosperms | Lamiaceae | *Elsholtzia stauntoni* | Beijing Botanical Garden, CAS, Beijing, China | BOP010179 | yes |
| 110 | Angiosperms | Lardizabalaceae | *Akebia trifoliata* | Beijing Botanical Garden, CAS, Beijing, China | BOP010268 | yes |
| 111 | Angiosperms | Lauraceae | | Sanxia Rare Plant Garden, Chongqing, China | BOP017777 | yes |
| 112 | Angiosperms | Lemnaceae | | Beijing Botanical Garden, CAS, Beijing, China | BOP010526 | yes |
| 113 | Angiosperms | Liliaceae | | Beijing Botanical Garden, CAS, Beijing, China | BOP010517 | yes |
| 114 | Angiosperms | Linaceae | *Reinwardtia indica* | Xishuangbanna Botanical Garden, CAS,Yunnan,China | BOP016900 | yes |
| 115 | Angiosperms | Loganiaceae | *Buddleja albiflora* | Beijing Botanical Garden, CAS, Beijing, China | BOP010282 | yes |
| 116 | Angiosperms | Loranthaceae | *Sarcandra glabra* subsp. *brachystachy* | Ledong, Hainan, China | 0207026 | yes |
| 117 | Angiosperms | Lythraceae | *Lagerstroemia indica* | Beijing Botanical Garden, CAS, Beijing, China | BOP010171 | yes |
| 118 | Angiosperms | Magnoliaceae | *Magnolia amoena* | Beijing Botanical Garden, CAS, Beijing, China | BOP010098 | yes |
| 119 | Angiosperms | Malpighiaceae | *Thryallis glacuca* | Xishuangbanna Botanical Garden, Yunnan, China | B9 | yes |
| 120 | Angiosperms | Malvaceae | *Hibiscus syriacus* | Beijing Botanical Garden, CAS, Beijing, China | BOP010090 | yes |
| 121 | Angiosperms | Marantaceae | *Thalia dealbata* | Beijing Botanical Garden, CAS, Beijing, China | BOP010525 | yes |

| | | | | | | |
|---|---|---|---|---|---|---|
| 122 | Angiosperms | Melastomataceae | *Tibouchina hetero* | Xishuangbanna Botanical Garden, CAS,Yunnan,China | BOP016874 | yes |
| 123 | Angiosperms | Meliaceae | *Melia azedarach* | Beijing Botanical Garden, CAS, Beijing, China | BOP010460 | yes |
| 124 | Angiosperms | Melianthaceae | *Melianthus major* | Melbourne Botanic Garden, Melbourne, Australia | BOP017846 | yes |
| 125 | Angiosperms | Menispermaceae | *Menispermum dauricum* | Beijing Botanical Garden, CAS, Beijing, China | BOP010535 | yes |
| 126 | Angiosperms | Menyanthaceae | *Nymphoides peltatum* | Xishuangbanna Botanical Garden, CAS,Yunnan,China | BOP016920 | yes |
| 127 | Angiosperms | Moraceae | *Ficus carica* | Beijing Botanical Garden, CAS, Beijing, China | BOP010474 | yes |
| 128 | Angiosperms | Moringaceae | *Moringa oleifera* | Xishuangbanna Botanical Garden, Yunnan, China | BOP017749 | yes |
| 129 | Angiosperms | Musaceae | | Xishuangbanna Botanical Garden, Yunnan, China | BOP017732 | yes |
| 130 | Angiosperms | Myoporaceae | *Myoporum Parvifolium* | Adelaide Botanic Garden, Adelaide, Australia | BOP017820 | yes |
| 131 | Angiosperms | Myristicaceae | *Horsfieldia amygdalina* | Xishuangbanna Botanical Garden, CAS,Yunnan,China | BOP016890 | yes |
| 132 | Angiosperms | Myrsinaceae | *Brassaiopsis glomerulata* | Ledong, Hainan, China | 0230219 | yes |
| 133 | Angiosperms | Myrtaceae | *Syzygium samarangense* | Beijing Botanical Garden, CAS, Beijing, China | BOP010488 | yes |
| 134 | Angiosperms | Nelumbonaceae | *Nelumbo nucifera* | Beijing Botanical Garden, CAS, Beijing, China | A12 | yes |
| 135 | Angiosperms | Notofagaceae | *Notofagus codonandra* | Melbourne Botanic Garden, Melbourne, Australia | BOP017843 | yes |
| 136 | Angiosperms | Nyctaginaceae | *Pisnia umbellifera* | Adelaide Botanic Garden, Adelaide, Australia | BOP017811 | yes |
| 137 | Angiosperms | Nymphaeaceae | | Beijing Botanical Garden, CAS, Beijing, China | BOP010524 | yes |
| 138 | Angiosperms | Ochnaceae | *Ochna integerrima* | Xishuangbanna Botanical Garden, CAS,Yunnan,China | BOP016879 | yes |
| 139 | Angiosperms | Oleaceae | *Fontanesia phillyreoides* subsp. *fortun* | Beijing Botanical Garden, CAS, Beijing, China | BOP010056 | yes |
| 140 | Angiosperms | Onagraceae | *Epilobium hirsutum* | Beijing Botanical Garden, CAS, Beijing, China | BOP010529 | yes |
| 141 | Angiosperms | Orchidaceae | *Cymbidium aloifolium* | Beijing Botanical Garden, CAS, Beijing, China | BOP010557 | yes |
| 142 | Angiosperms | Orobanchaceae | | Xijiashan Botanical Garden, Tahe, Heilongjiang, China | BOP016748 | yes |
| 143 | Angiosperms | Oxalidaceae | | Beijing Botanical Garden, CAS, Beijing, China | BOP010513 | yes |
| 144 | Angiosperms | Paeoniaceae | *Paeonia decomposita* ssp. *decomposit* | Maerkang, Sichuan, China | BOP001444 | yes |
| 145 | Angiosperms | Palmaceae | *Pinanga* sp. | Ledong, Hainan, China | 1730117 | yes |
| 146 | Angiosperms | Pandanaceae | *Pandanus veitchii* | Beijing Botanical Garden, CAS, Beijing, China | BOP010496 | yes |
| 147 | Angiosperms | Papaveraceae | | Beijing Botanical Garden, CAS, Beijing, China | BOP010515 | yes |
| 148 | Angiosperms | Parnassiaceae | | Wulumuqi, Xinjiang, China | BOP016962 | yes |
| 149 | Angiosperms | Passifloraceae | *Passiflora caerulea* | Xishuangbanna Botanical Garden, Yunnan, China | A7 | yes |
| 150 | Angiosperms | Pedaliaceae | *Uncarina peltata* | Adelaide Botanic Garden, Adelaide, Australia | BOP017816 | yes |
| 151 | Angiosperms | Penthoraceae | *Penthorum chinense* | Beijing, China | PE01 | yes |

| | | | | | | | |
|---|---|---|---|---|---|---|---|
| 152 | Angiosperms | Philydraceae | *Helmholtzia glaberrima* | Melbourne Botanic Garden, Melbourne, Australia | BOP017854 | yes | |
| 153 | Angiosperms | Phrymaceae | *Phryma leptostachya* var. *asiatica* | Mt. Maoershan, Heilongjiang, China | BOP017754 | yes | |
| 154 | Angiosperms | Phyllanthaceae | *Phyllanthus emblica* | Xishuangbanna Botanical Garden, CAS,Yunnan,China | BOP016906 | yes | |
| 155 | Angiosperms | Phytolaccaceae | *Phytolacca acinosa* | Mt.Jigongshan, Xinyang, Henan, China | BOP017598 | yes | |
| 156 | Angiosperms | Pittosporaceae | *Pittosporum illicioides* | Mt.Jigongshan, Xinyang, Henan, China | BOP017202 | yes | |
| 157 | Angiosperms | Plantaginaceae | *Plantago depressa* | Beijing Botanical Garden, CAS, Beijing, China | BOP010502 | yes | |
| 158 | Angiosperms | Platanaceae | *Platanus orientalis* | Beijing Botanical Garden, CAS, Beijing, China | BOP010462 | yes | |
| 159 | Angiosperms | Plumbaginaceae | *Plumbago zeylanica* | Xishuangbanna Botanical Garden, CAS,Yunnan,China | BOP016903 | yes | |
| 160 | Angiosperms | Polemoniaceae | *Phlox longifolia* | Beijing Botanical Garden, CAS, Beijing, China | BOP010512 | yes | |
| 161 | Angiosperms | Polygalaceae | | Tahe, Heilongjiang, China | BOP016851 | yes | |
| 162 | Angiosperms | Polygonaceae | *Fallopia multiflora* | Beijing Botanical Garden, CAS, Beijing, China | BOP010146 | yes | |
| 163 | Angiosperms | Pontederiaceae | *Eichhornia crassipes* | Beijing Botanical Garden, CAS, Beijing, China | BOP017717 | yes | |
| 164 | Angiosperms | Portulacaceae | | Beijing Botanical Garden, CAS, Beijing, China | BOP010505 | yes | |
| 165 | Angiosperms | Potamogetonaceae | | Beijing Botanical Garden, CAS, Beijing, China | BOP010521 | yes | |
| 166 | Angiosperms | Primulaceae | | Beijing Botanical Garden, CAS, Beijing, China | BOP010532 | yes | |
| 167 | Angiosperms | Proteaceae | *Grevillea robusta* | Beijing Botanical Garden, CAS, Beijing, China | BOP010494 | yes | |
| 168 | Angiosperms | Punicaceae | *Punica granatum* | Beijing Botanical Garden, CAS, Beijing, China | BOP010501 | yes | |
| 169 | Angiosperms | Pyrolaceae | | Xijiashan Botanical Garden, Tahe, Heilongjiang, China | BOP016637 | yes | |
| 170 | Angiosperms | Ranunculaceae | *Clematis florida* | Beijing Botanical Garden, CAS, Beijing, China | BOP010450 | yes | |
| 171 | Angiosperms | Restionaceae | *Elegia capensis* | Melbourne Botanic Garden, Melbourne, Australia | BOP017853 | | no |
| 172 | Angiosperms | Rhamnaceae | *Rhamnus globosa* | Beijing Botanical Garden, CAS, Beijing, China | BOP010001 | yes | |
| 173 | Angiosperms | Rhizophoraceae | *Carallia diphopetala* | Xishuangbanna Botanical Garden, CAS,Yunnan,China | BOP016921 | yes | |
| 174 | Angiosperms | Rosaceae | *Prunus davidiana* | Beijing Botanical Garden, CAS, Beijing, China | BOP010009 | yes | |
| 175 | Angiosperms | Rubiaceae | *Leptodermis oblonga* | Beijing Botanical Garden, CAS, Beijing, China | BOP010280 | yes | |
| 176 | Angiosperms | Rutaceae | *Zanthoxylum bungeanum* | Beijing Botanical Garden, CAS, Beijing, China | BOP010483 | yes | |
| 177 | Angiosperms | Sabiaceae | *Meliosma simplicifolia* | Xishuangbanna Botanical Garden, CAS,Yunnan,China | BOP016913 | yes | |
| 178 | Angiosperms | Salicaceae | *Salix babylonica* | Beijing Botanical Garden, CAS, Beijing, China | BOP010457 | yes | |
| 179 | Angiosperms | Salvadoraceae | *Heritiera littoralis* | Xishuangbanna Botanical Garden, CAS,Yunnan,China | BOP016922 | yes | |
| 180 | Angiosperms | Samydaceae | *Casearia aequilateralis* | Ledong, Hainan, China | 0216129 | yes | |
| 181 | Angiosperms | Santalaceae | *Santalum album* | Xishuangbanna Botanical Garden, CAS,Yunnan,China | BOP016893 | yes | |

| | | | | | | |
|---|---|---|---|---|---|---|
| 182 | Angiosperms | Santalaceae | *Thesium chinense* | Mt. Xinglong, Gansu, China | BOP017759 | yes |
| 183 | Angiosperms | Sapindaceae | *Koelreuteria paniculata* | Beijing Botanical Garden, CAS, Beijing, China | BOP010197 | yes |
| 184 | Angiosperms | Sapotaceae | *Manilkara zapota* | Beijing Botanical Garden, CAS, Beijing, China | BOP010544 | yes |
| 185 | Angiosperms | Sarcospermatacea | *Sarcosperma laurinum* | Ledong, Hainan, China | 0230146 | yes |
| 186 | Angiosperms | Saurauiaceae | *Saurauia tristyla* | Kunming Botanical Garden, CAS,Yunnan,China | BOP016925 | yes |
| 187 | Angiosperms | Saururaceae | *Houttuynia cordata* | Mt.Jigongshan, Xinyang, Henan, China | BOP017042 | yes |
| 188 | Angiosperms | Saxifragaceae | *Philadelphus pekinensis* | Beijing Botanical Garden, CAS, Beijing, China | BOP010046 | yes |
| 189 | Angiosperms | Schisandraceae | *Schisandra chinensis* | Beijing Botanical Garden, CAS, Beijing, China | BOP010144 | yes |
| 190 | Angiosperms | Scrophulariaceae | *Buddleja lindleyana* | Beijing Botanical Garden, CAS, Beijing, China | BOP010309 | yes |
| 191 | Angiosperms | Simaroubaceae | *Ailanthus altissima* | Beijing Botanical Garden, CAS, Beijing, China | BOP010321 | yes |
| 192 | Angiosperms | Smilacaceae | *Smilax china* | Mt. Guipi, Yunnan, China | BOP017764 | yes |
| 193 | Angiosperms | Solanaceae | *Datura stramonium* | Beijing Botanical Garden, CAS, Beijing, China | BOP010539 | yes |
| 194 | Angiosperms | Sonneratiaceae | *Duabanga grandiflora* | Xishuangbanna Botanical Garden, CAS,Yunnan,China | BOP016889 | yes |
| 195 | Angiosperms | Stachyuraceae | *Stachyurus himalaicus* | Kunming Botanical Garden, CAS,Yunnan,China | BOP016926 | yes |
| 196 | Angiosperms | Staphyleaceae | *Tapiscia sinensis* | Xishuangbanna Botanical Garden, CAS,Yunnan,China | BOP016915 | yes |
| 197 | Angiosperms | Stemonaceae | *Stemona tuberosa* | Xishuangbanna Botanical Garden, Yunnan, China | BOP017739 | yes |
| 198 | Angiosperms | Sterculiaceae | *Scaphium wallichii* | Xishuangbanna Botanical Garden, Yunnan, China | BOP017745 | yes |
| 199 | Angiosperms | Styracaceae | *Styrax* sp. | Beijing Botanical Garden, CAS, Beijing, China | BOP010242 | yes |
| 200 | Angiosperms | Symplocaceae | *Symplocos anomala* | Ledong, Hainan, China | 1628143 | yes |
| 201 | Angiosperms | Tamaricaceae | *Tamarix chinensis* | Beijing Botanical Garden, CAS, Beijing, China | BOP010456 | yes |
| 202 | Angiosperms | Tetracentraceae | *Tetracentron sinense* | Kunming Botanical Garden, CAS,Yunnan,China | BOP016924 | yes |
| 203 | Angiosperms | Tetragoniaceae | *Tetragonia implexicoma* | Adelaide Botanic Garden, Adelaide, Australia | BOP017806 | yes |
| 204 | Angiosperms | Tetramelaceae | *Tetrameles nudiflora* | Xishuangbanna Botanical Garden, CAS,Yunnan,China | BOP016904 | yes |
| 205 | Angiosperms | Theaceae | *Camellia sinensis var. assamica* | Xishuangbanna Botanical Garden, Yunnan, China | B10 | yes |
| 206 | Angiosperms | Thymelaeaceae | *Edgeworthia chrysantha* | Beijing Botanical Garden, CAS, Beijing, China | BOP010487 | yes |
| 207 | Angiosperms | Tiliaceae | *Tilia cordata* | Beijing Botanical Garden, CAS, Beijing, China | BOP010096 | yes |
| 208 | Angiosperms | Trapaceae | *Trapa* sp. | Beijing Botanical Garden, CAS, Beijing, China | BOP017718 | yes |
| 209 | Angiosperms | Typhaceae | *Typha* sp. | Beijing Botanical Garden, CAS, Beijing, China | BOP017719 | yes |
| 210 | Angiosperms | Ulmaceae | *Ulmus laciniata* | Mt. Maoershan, Heilongjiang, China | BOP017756 | yes |
| 211 | Angiosperms | Urticaceae | | Beijing Botanical Garden, CAS, Beijing, China | BOP010510 | yes |

| | | | | | | | |
|---|---|---|---|---|---|---|---|
| 212 | Angiosperms | Valerianaceae | *Patrinia monandra* | Mt. Xinglong, Gansu, China | BOP017760 | yes | |
| 213 | Angiosperms | Verbenaceae | *Caryopteris clandonensis* | Beijing Botanical Garden, CAS, Beijing, China | BOP010086 | yes | |
| 214 | Angiosperms | Violaceae | *Viola prionantha* | Beijing Botanical Garden, CAS, Beijing, China | BOP010506 | yes | |
| 215 | Angiosperms | Vitaceae | *Vitis* sp. | Beijing Botanical Garden, CAS, Beijing, China | BOP010263 | yes | |
| 216 | Angiosperms | Xanthorrhoeaceae | *Xanthoceras sorbifolia* | Beijing Botanical Garden, CAS, Beijing, China | BOP010107 | yes | |
| 217 | Angiosperms | Xanthorrhoeaceae | *Xamthorrhoea australis* | Adelaide Botanic Garden, Adelaide, Australia | BOP017808 | yes | |
| 218 | Angiosperms | Zingiberaceae | *Alpinia zerumbet* | Beijing Botanical Garden, CAS, Beijing, China | BOP010548 | | no |
| 219 | Angiosperms | Zygophyllaceae | *Tribulus terrestris* | Beijing Botanical Garden, CAS, Beijing, China | BOP010560 | yes | |
| | PCR success | | | | | | 98.17% |

**Table S3. Taxon-specific *ycf1* primers in the event of failure of the universal primers listed in Table 1.**

| Groups | family | Forward primer (5'-3') | Reverse primer (5'-3') |
|---|---|---|---|
| Bryophytes | Amblystegiaceae | | AGATTTTTCCAAGAGCGTTCTAGTA |
| Bryophytes | Aneuraceae | AGTAAAACGCATAATTTATCGAAC | GAGTCTTTTCGACGATTCCTTGAGA |
| Bryophytes | Anthocerotaceae | AGTCAAACGTCTTATTTATCGAAC | AAATCTTTCCAAGAATTTTTAATCG |
| Bryophytes | Bartramiaceae | GGTAAAACGCCTTATTTATCGAAC | |
| Bryophytes | Calymperaceae | AGTAAAACGTCTTATATATCGAAT | |
| Bryophytes | Cryphaeaceae | | AGATTTTTCCAAGAGCGTTCTAGTA |
| Bryophytes | Dicranaceae | | AAATTTTTCCATGAGCGTTCTAGTA |
| Bryophytes | Ditrichaceae | | AAATTTTTCCATGAGCGTTCTAATA |
| Bryophytes | Entodontaceae | AGTAAAACGTCTTATTTATCGAAC | |
| Bryophytes | Fabroniaceae | AGTAAAACGTCTTATTTATCGAAC | |
| Bryophytes | Fontinalaceae | | AGATTTTTCCAAGAGCGTTCTAGTA |
| Bryophytes | Funariaceae | GGTCAAACGGATAATTTATCGAAC | AAATTTTTCCAAGAACGTACTAATA |
| Bryophytes | Hookeriaceae | | AGATTTTTCCAACCTTGTTCTAGTA |
| Bryophytes | Hypnaceae | | AGATTTTTCCAAGAGCGTTCTAGTA |
| Bryophytes | Hypopterygiaceae | | AGATTTTTCCAAGAGCGTTCTAGTA |
| Bryophytes | Leskeaceae | | AGATTTTTCCAAGAGCGTTCTAGTA |
| Bryophytes | Leucodontaceae | | AGATTTTTCCAAGAGCGTTCTAGTA |
| Bryophytes | Marchantiaceae | AGTTAAACGTATTATTTATCGAAC | AAATTTTTCCAAGAAATTTTAAGCG |
| Bryophytes | Meteoriaceae | AGTAAAACGTATTGTTTATCGAAC | ATATTTTTCCAAGAGCGTTCTAGTA |
| Bryophytes | Orthotrichaceae | AGTAAAACGCCTTGTTTATCGAAC | |
| Bryophytes | Plagiotheciaceae | | AGATTTTTCCAAGAGCGTTCTAGTA |
| Bryophytes | Pogotrichaceae | AGTAAAGCGTCTTATTCATCGAAC | |
| Bryophytes | Pottiaceae | AGTAAAACGCCTTATTTATCGAAC | AAATTTTTCCAAGAGCGTTCTAGTA |
| Bryophytes | Pterobryaceae | | AGATTTTTCCAAGAGCGTTCTAGTA |
| Bryophytes | Ptilidiaceae | GGTGAAACGTATAATTCATCGAAC | AAATTTTTCCAATAAATTTTGAATG |
| Bryophytes | Ptychomitriaceae | | AAATTTTTCCAAGAGCGTTCTAGTA |
| Bryophytes | Racopiaceae | | AGATTTTTCCAAGAGCGTTCTAGTA |
| Bryophytes | Splachnaceae | AGTAAAACGAGTTATTTATAGAAC | AAATTTTTCCAAGACCGTACTAGTA |
| Bryophytes | Thuidiaceae | | AGATTTTTCCAAGAGCGTTCTAGTA |
| Bryophytes | Trachypodaceae | | AGATTTTTCCAAGAGCGTTCTAGTA |

| | | | |
|---|---|---|---|
| Monilophytes | Adiantaceae | TCTCAAACCTGTATTTATATTGATATGTGG | ATCTGGATCTAACCACGATAGCAAATCA |
| Monilophytes | Adiantaceae | TCTCAAGCTTGTCTCTATGCCGATATATGG | ATTTAGATTTAGCCATGGTATTAAGTCA |
| Monilophytes | Angiopteridaceae | TCTCACGCGTATATACTGGATAGGTTGTGG | TTCTGGAAATAACCATAGCATTAGATTG |
| Monilophytes | Aspidiaceae | | CCCTGTAACTAGCCACGGCATCAAATCA |
| Monilophytes | Aspidiaceae | TCTCAAGCTTATCTCTGCAACAGTATTTGG | ATCCGTAACTAGCCACGGCATTAAATCA |
| Monilophytes | Athyriaceae | CCTCAAGCTTATCTCTATAACAGTATTTGG | ATCTGTAACTAGCCACGGCATCAAACCA |
| Monilophytes | Athyriaceae | TCTCAAGCTTGTCTCTGTGGCGATATATGG | ATCTGGAATTAGCCACGGCATCAAATCA |
| Monilophytes | Blechnaceae | TCTCAAGCTTATCTTTATAACAGTATTTGG | ATCTGTAACTAGCCACGGCATCAAATCA |
| Monilophytes | Cyatheaceae | TCTCAAGCTCGTCTTTATGACAATATGTGG | ATCCGGAACTACCCATAGCATCAAATCA |
| Monilophytes | Davalliaceae | TCTCAAGCTTATATCTATAACAGTATTTGG | ATCTGTAACTAGCCAAGGCATCAAATCA |
| Monilophytes | Dennstaedtiaceae | TCTCAAGCTTGTCTCTGTGGCGATATATGG | ATCTGGAACTAGCCACGGCATCAAATCA |
| Monilophytes | Drynariaceae | | ATCTGTTACTAACCACGGCATCAAATCA |
| Monilophytes | Dryopteridaceae | TCCCAAGCTTATCTTTTTAACAGTATTTGG | ATCTGCAACTAGCCACGGTATCAAATCA |
| Monilophytes | Elaphoglossaceae | TCTCAAGCTGATCTCTATAACAGTATTTGG | ATCTGTAACTAGCCATGGCATCAAATCA |
| Monilophytes | Equisetaceae | TCGCAAGCATATATATTCCATCAATTATGG | |
| Monilophytes | Huperziaceae | TCTCAAGCAGATGTTTTTCATGGGGTACGG | TTCAGGGAATAACCACAACTTCATCTTG |
| Monilophytes | Hypolepidaceae | TCTCAAGCTTGTCTCTGCGGCGATATATGG | ATCTGGAATTAGCCACGGCATCAAATCA |
| Monilophytes | Isoetaceae | TCCCAAGCATATGTATTTCACAAAATATGG | CTCTGGATATAACCATGAATATAAATTG |
| Monilophytes | Lomariopsidaceae | | ATCTGTAACTAGCCATGGCATCACATCA |
| Monilophytes | Lygodiaceae | TCTCACGCTTACCTCTACGAAAAAACATGG | ATCCGTAATTGACCATAAAGCCAAATCT |
| Monilophytes | Marsileaceae | TCGCAGGCTTATATTTATGAGAATTTGTGG | ATCCGGAATCGTCCACAGCATCAGATCG |
| Monilophytes | Oleandraceae | | ATCTGTAACTAGCCACGGCATCAAATCA |
| Monilophytes | Onocleaceae | CCTCAAGCTTATCTACATAACACTATTTGG | ATCTGTAACTAGCCACGGCATCAAATCA |
| Monilophytes | Ophioglossaceae | TCTCAAGCAGATGTGTTTCATAGATTATGG | ATCCGGAACTAACCATAGTGTTAGATCA |
| Monilophytes | Polypodiaceae | TCTCAAGCTGATCTCTATAATAGTATTTGG | ATCTGTAACTAGCCACGGCATCAAATCA |
| Monilophytes | Psilotaceae | TCTCAAGCATATGTATTTCAAAAGTTATGG | ATTCGGAAGTAACCAGAGCATTAGATTA |
| Monilophytes | Pteridacae | TCTCAAGCTTGTCTCTATGCCGATATATGG | ATTTAGATTTAGCCATGGTATTAAGTCA |
| Monilophytes | Pteridaceae | TCTCAAGCTTGCCTCTATGCCGATTTATGG | ATCTGGATCTAACCACAGCATCAAATCA |
| Monilophytes | Selaginellaceae | TCTCAAGCATATGTACTTCACGGGGCATGG | ATCCGGCAGGAACCATAATATCAGTTTA |
| Monilophytes | Thelypteridaceae | CCCCAAGCTTATCTCTATAACAGTATTTGG | ATCTGTAACTAGCCACGGCATCAAATCA |

| | | | |
|---|---|---|---|
| Gymnosperms | Cephalotaxaceae | TTAAAGCTCTAAGCAATGGATTTCC | ATACGACCAATATTTTTGGCTATTAT |
| Gymnosperms | Cupressaceae | TTAGAGCTTTAAGCAATGGGTCTCC | CTACGACCAACATTTTTAGCTATTAT |
| Gymnosperms | Cycadaceae | TAGAAGCTCTAAGCAATGGATCCCC | ATACGACCAATATTTTTAGCTATTAT |
| Gymnosperms | Ephedraceae | CGGGAAGTACAAAGAAAGAAGCATT | ATGCGACCGATATTTTTAATACCAAT |
| Gymnosperms | Ginkgoaceae | TAGAAGCTCTAGGCAATGGATATAC | ATACGACCAATATTTTTAGCTATTAT |
| Gymnosperms | Gnetaceae | TTCTAGCTATAAGAAAAGAAGCGCT | ATACGTCCAACATTCTTGATTCCGAT |
| Gymnosperms | Pinaceae | TGAAAGCTTTAAGCAATGGATCTCC | ATACGACCGATATTCTTGACTATTAT |
| Gymnosperms | Podocarpaceae | TAAAAGCTCTAAGCAATGGATCTCC | GTACGAACAATATTCTTCGTTATTAT |
| Gymnosperms | Taxodiaceae | TGAGAGCTCTAAGYAATGGATCYCC | ACACGAGCGAAGTTTTTGGCTAYTAT |
| Gymnosperms | Welwitschiaceae | CGTCAGTTATTAGGAAAGAAACGTT | AAACGACCACAATTTTTTATTAAGAT |
| | | | |
| Angiosperms | Acanthaceae | ATATATGCCAAAGTGATGGAAAT | CCTCGTCGAAAGTCGGGTTGGTGTGAAT |
| Angiosperms | Acoraceae | ATACATGTCTGAATAATGGGAAA | TCCCGACTAAAATCTGATTGTTGCGCGT |
| Angiosperms | Altingiaceae | ACACATGTCCAAGTGATGGAAAA | TCCCGACGAAAATCCGATTGTTGCGAGT |
| Angiosperms | Amborellaceae | ACACATGTCCAAGTGATGGAAAA | TCCCGACGAAAATCAGGTTTCTGTGTGT |
| Angiosperms | Apiaceae | ATACATGCCGAAGTGATGGAAAA | TCTCGACGAAAATCTGATTCTTCTGAAT |
| Angiosperms | Apocynaceae | ACACATGCCAAAGTGATGGAAAA | CCTCGCCGAAAGTCCGATTGTTGTGAAT |
| Angiosperms | Araceae | ATACATGTCCAAGTGATGGAAAA | TCCCGACGAAAATCCGATTGTTGTGAGT |
| Angiosperms | Araliaceae | ATACATGCCGAAGTGATGGAAAA | TTTCGACGAAAATCCGATTCTTGCAAAT |
| Angiosperms | Arecaceae | ATACATGTCCAAGTAATGGGAAA | TCTCTACGAAAATCTGATTGTTGCGAGT |
| Angiosperms | Berberidaceae | ATACATGTATAAGTGACGGAAAA | TCTCGACGAAAATCTGGTTGTTGCGAAT |
| Angiosperms | Brassicaceae | ATACATGCCAAAGTGATGGAAAA | TCTCGACGAAAATCAGATTGTTGTGAAT |
| Angiosperms | Buxaceae | ATACATGTAAAAATGATGGAAAA | TCTCGACGAAAATCTGATTGCTGCGAAT |
| Angiosperms | Calycanthaceae | ACAAATGTCCAGTTGATGGAAAA | TCCCGACGAAAATCCGATTGTTGCGAAT |
| Angiosperms | Campanulaceae | ATACATGCCGAAGCGATGGAAAA | CCCGGACGATAATTGTCCCGGTTCGAAA |
| Angiosperms | Caricaceae | ATACATGTCAAAGTGATGGAAAA | TCTCGACGAAAATCTGATTGTTGTGAAT |
| Angiosperms | Caryophyllaceae | ATACATGTCAAAGCGATGGAAAA | TCTCGAGCAAAATCAGTTTCGCTTGGAT |
| Angiosperms | Ceratophyllaceae | ACACATGTACAAGTGACGGAAAA | TTCCGACGAAAATCCGGTTGGTGCGAGT |
| Angiosperms | Chenopodiaceae | ATACATGTCAAAATGATGGAAAA | TCTCGACGAAAATCAGATTCTTGTGGAT |
| Angiosperms | Chloranthaceae | ACACATGCCCAAGTGATGGAAAA | TTCCGACGAAAATCCGATTGTTGCGAGT |
| Angiosperms | Convolvulaceae | ATACACAACAAAGTGATGGAAAA | CCGTGCTTAAAATCGGGTTGGTGTGCAT |
| Angiosperms | Corynocarpaceae | ATACATGTCAAAGTGAGGGAAAA | TCTCGACGAAAATCCGATAGTTGTGAAT |

| | | | |
|---|---|---|---|
| Angiosperms | Cucurbitaceae | ACACATATCAAAGTGATGGAAAA | CCTTGACGAAAATCGGATTGTTGCGGAT |
| Angiosperms | Dasypogonaceae | ATACGTGTCCAAGTGATGGAAAA | TCCCGACGAAAATCTGATTGTTGCGAGT |
| Angiosperms | Dioscoreaceae | ATACATGTCCAAGTGATGGAAAA | TCCCGACGAAAATCGGATTGTTGTGAGT |
| Angiosperms | Euphorbiaceae | ATACATGTCGAAGTGATGGAAAA | TCTCGACGAAAGTCCGATTGTTGTGAAT |
| Angiosperms | Fabaceae | AAACATGTCAAAGTGATGGAAAA | TCTCTGTTAAACTCTGCGCCGGGTACAT |
| Angiosperms | Fagaceae | ATACATGTAAAAGTGATGGAAAA | TCTCGACGAAAATCCGATTGTTGTGAAT |
| Angiosperms | Geraniaceae | ATAAATGTCAAAGCGATGGAAAA | TTGCGACGAAAATGCGTGATCTTAGCGT |
| Angiosperms | Gesneriaceae | ACATATGCCAAAGTGATGGAAAA | CCTCGCCGAAAATCTGATTGTTGTGAAT |
| Angiosperms | Heliconiaceae | ATACATGTTCAAGTGATGGAAAA | CCCCGACGAAAATCAGGTTGTTGCGAGT |
| Angiosperms | Hydatellaceae | AAATAAGTACAAGTGCTGGAAAA | GAACGATCAAAATCGGGTTCGTCGACAA |
| Angiosperms | Hydrocharitaceae | ATCCATGTCCAAGTGATGGAAAA | TTTCGACGGAAATCTGGTGACTGTGAGT |
| Angiosperms | Illiciaceae | ATACATCAAAAAGGGATGGAAAC | TTCCTACGAAAATCTGATTTTTGTGAGT |
| Angiosperms | Lamiaceae | ACATACGTCAAAGTGATGGAAAA | TTCCGCCGAAAGTCTGATTGTTGTGAAT |
| Angiosperms | Lemnaceae | ATACATGTCCAAGTGATGGAAAA | TCCCGACGAAAATCCGATTGTTGTGAGT |
| Angiosperms | Malvaceae | ATACATGTCAAAGTGATGGAAAA | TCGCGCCGAAAATCCGATTGTTGCGAAT |
| Angiosperms | Melianthaceae | ATACATGCCGAAGTGATGGAAAC | TCTCGACGAAAATCCGATTCTTGCAAAT |
| Angiosperms | Moraceae | ATACATGTGAAAGCGATGGAAAA | TCTCGACGAAAATCCGATTGTTGCGAAT |
| Angiosperms | Myrtaceae | ATATATGTCGAAACAATGGAAAA | TCTCGACGAAAATCTGATTGTTGTGAAT |
| Angiosperms | Nelumbonaceae | ATACATGCCAAAGTGATGGAAAA | TTCCTACGAAAATCCGATTGTTGTGAAT |
| Angiosperms | Nymphaeaceae | ACACATGCGCAAGTGATGGAAAA | TCCCGACGAAAATCCGATTGTTGCGAGT |
| Angiosperms | Nymphaeaceae | ATACATGCGCAAGTGATGGAAAG | TCACGGCGAAAATCTGATTGTTGCGAGT |
| Angiosperms | Oleaceae | ACACATGCCAAAGTGCTGGAAAG | CCTCGCCTAAAATCTGATTGTTGTGAAT |
| Angiosperms | Orchidaceae | TTACATGTAAAAGTGATGGTAAA | TTGCGACGAAAATCCGATTGTTGTGAGT |
| Angiosperms | Orchidaceae | ATACATATCCACGTAATGGAAGA | TCTCTCCGAAAATCCGACTGTTGGGAAT |
| Angiosperms | Orobanchaceae | ACATATGCAAAAATGATGGAAAA | GCTCGACGAAAGTCTGATTTTTTTGAAA |
| Angiosperms | Paeoniaceae | ACACATGCCGAAGTGATGGAAAA | TTTCGACGAAAATCTGATTGTTGCGAAT |
| Angiosperms | Pedaliaceae | ACGTATCCAAAAGTGATGGAAAA | TCTCGCTCAAAGTCTGATTGTTGTGAAT |
| Angiosperms | Penthoraceae | ACACATGTCAAAGTGATGGAAAA | TCTCGACGAAAATCTGATTGTTGTGAAT |
| Angiosperms | Platanaceae | ATACATGCCAAAGTGATGGAAAA | TCCCGACGAAAATCCGGTTGTTGTGAAT |
| Angiosperms | Polygonaceae | ATACATGTCAAAGTGATGGAAAA | TCTCGACGAAAATCCGCTTCATAGGGAT |
| Angiosperms | Primulaceae | ATACATGTCAAAGTTATGGAAAA | TCTCGACGAAAATCCGATTGTTGCGAAT |
| Angiosperms | Ranunculaceae | ATACATGCCAAAGTGATGGAAAA | TCTCGGCGAAAATCGGATTGTTCCAAAT |

| | | | |
|---|---|---|---|
| Angiosperms | Rosaceae | ATACATGTCAAAGTGATGGAAAA | TCTCGACGAAAATCCGATTGTTGTGAAT |
| Angiosperms | Rubiaceae | AGATCTACGAAAGCAATGGGAAA | CTTCTTCGAAAATCCGACTGTTGCGAAT |
| Angiosperms | Rutaceae | ATGCATGTCGAAGTGATGGAAAA | TATCGACGAAAATCCGATTGTTCCAAAT |
| Angiosperms | Salicaceae | CTACATGTCGAAGTGATGGAAAA | TCTCGACGAAAGTCCGATTGTTGTGAAT |
| Angiosperms | Smilacaceae | ATACATGTCCAAGCGCTGGAAAA | TTCCGACGAAAATCCGATTGTTGCGAGT |
| Angiosperms | Solanaceae | ACACATGTAAAAGTGATGGAAAA | CCTCGCCGAAAATCTGATTGTTGCGAAT |
| Angiosperms | Sterculiaceae | ATACATGTCAAAGTGATGGAAAA | TCTCGACGAAAATCCGATTGTTGCGAAT |
| Angiosperms | Theaceae | ATACACGGCAAAGTTATGGAAAA | TCTCGACGAAAATCAGATTGTTGCGAAT |
| Angiosperms | Trochodendraceae | ATACATGTCAAAGTGACGGAAAA | TCCCGACGAAAATCTGCTTGTTGAGAAT |
| Angiosperms | Typhaceae | ATACATGCCCAAGTGATGGAAAA | TCCCGACGATAATCAGATTGTTGCGAGT |
| Angiosperms | Vitaceae | ATACATGCCTCAGTGATGGAAAA | TCTCGACGAAAATCCGATTGTTGTGAAT |
| Angiosperms | Winteraceae | CCACTACTTGTACATGTGGAAAA | TTCCGACGAAAATCCGATTGTTGTGAGT |
| Angiosperms | Zingiberaceae | ATACATGTTTAAGTGATGGAAAA | CCTCGATGAAAATCAGGTTGTTCTGAGT |

**Table S4. The 490 samples collected from the Beijing Botanical Garden, CAS for candidate barcode resolution testing.**

| | Family | Species | Voucher | ***rbcL* b** | ***matK*** | ***ycf1* b** |
|---|---|---|---|---|---|---|
| 1 | Aceraceae | *Acer davidii* | BOP010091 | KP088448 | KP088935 | KP088140 |
| 2 | Aceraceae | *Acer ginnala* | BOP010199 | KP088449 | KP088936 | KP088227 |
| 3 | Aceraceae | *Acer palmatum* | BOP010210 | KP088450 | KP088937 | KP088237 |
| 4 | Aceraceae | *Acer pseudo-sieboldianum* | BOP010209 | KP088451 | KP088938 | KP088236 |
| 5 | Aceraceae | *Acer semenovii* | BOP010201 | KP088452 | KP088939 | KP088229 |
| 6 | Aceraceae | *Acer* sp. | BOP010198 | KP088453 | KP088940 | KP088226 |
| 7 | Aceraceae | *Acer* sp. | BOP010211 | KP088454 | KP088941 | KP088238 |
| 8 | Aceraceae | *Acer stenolobum* | BOP010200 | KP088455 | KP088942 | KP088228 |
| 9 | Aceraceae | *Acer truncatum* | BOP010011 | KP088456 | KP088943 | KP088081 |
| 10 | Aceraceae | *Acer truncatum* | BOP010195 | KP088457 | KP088944 | KP088224 |
| 11 | Actinidiaceae | *Actinidia chinensis* | BOP010142 | KP088458 | | |
| 12 | Actinidiaceae | *Actinidia* sp. | BOP010141 | KP088459 | | |
| 13 | Actinidiaceae | *Actinidia* sp. | BOP010479 | KP088460 | KP088945 | |
| 14 | Alangiaceae | *Alangium chinense* subsp. *pauciflorum* | BOP010310 | KP088468 | KP088952 | KP088313 |
| 15 | Alangiaceae | *Alangium platanifolium* | BOP010311 | KP088469 | KP088953 | KP088314 |
| 16 | Anacardiaceae | *Cotinus coggygria* | BOP010064 | KP088547 | KP089025 | KP088127 |
| 17 | Anacardiaceae | *Pistacia chinensis* | BOP010204 | KP088746 | KP089206 | KP088232 |
| 18 | Anacardiaceae | *Rhus chinensis* | BOP010160 | KP088829 | KP089289 | KP088193 |
| 19 | Anacardiaceae | *Rhus chinensis* | BOP010359 | KP088830 | KP089290 | KP088355 |
| 20 | Anacardiaceae | *Rhus potaninii* | BOP010163 | KP088831 | KP089291 | KP088195 |
| 21 | Anacardiaceae | *Rhus punjabensis var. sinica* | BOP010074 | KP088832 | KP089292 | KP088132 |
| 22 | Anacardiaceae | *Rhus typhina* | BOP010203 | KP088833 | KP089293 | KP088231 |
| 23 | Anacardiaceae | *Toxicodendron delavayi* | BOP010202 | KP088891 | KP089347 | KP088230 |
| 24 | Anacardiaceae | *Toxicodendron vernicifluum* | BOP010484 | | KP089348 | KP088408 |
| 25 | Apocynaceae | *Apocynum venetum* | BOP010531 | KP088474 | KP088957 | KP088422 |
| 26 | Apocynaceae | *Nerium indicum* | BOP010558 | KP088700 | KP089165 | KP088428 |

| | | | | | | |
|---|---|---|---|---|---|---|
| 27 | Araliaceae | *Aralia chinensis* | BOP010322 | KP088475 | KP088958 | KP088322 |
| 28 | Araliaceae | *Eleutherococcus gracilistylus* | BOP010323 | KP088581 | KP089056 | KP088323 |
| 29 | Araliaceae | *Eleutherococcus sessiflorus* | BOP010019 | KP088582 | KP089057 | KP088089 |
| 30 | Araliaceae | *Hedera* sp. | BOP010489 | KP088628 | KP089102 | KP088412 |
| 31 | Araliaceae | *Kalopanax septemlobus* | BOP010325 | KP088641 | KP089114 | KP088325 |
| 32 | Araliaceae | *Tetrapanax papyrifer* | BOP010454 | KP088879 | KP089336 | KP088390 |
| 33 | Aristolochiaceae | *Aristolochia manshuriensis* | BOP010101 | KP088477 | KP088960 | KP088149 |
| 34 | Aristolochiaceae | *Callicarpa americana* | BOP010293 | KP088501 | KP088984 | KP088298 |
| 35 | Aristolochiaceae | *Callicarpa dichotoma* | BOP010295 | KP088503 | KP088986 | KP088299 |
| 36 | Aristolochiaceae | *Callicarpa* sp. | BOP010135 | KP088504 | KP088985 | KP088175 |
| 37 | Asteraceae | *Myripnois dioica* | BOP010410 | KP088698 | | KP088359 |
| 38 | Berberidaceae | *Berberis brachypoda* | BOP010078 | KP088478 | KP088961 | |
| 39 | Berberidaceae | *Berberis brachypoda* | BOP010082 | KP088479 | KP088962 | |
| 40 | Berberidaceae | *Berberis diaphana* | BOP010069 | KP088480 | KP088963 | |
| 41 | Berberidaceae | *Berberis* sp. | BOP010067 | KP088481 | KP088964 | |
| 42 | Berberidaceae | *Berberis* sp. | BOP010070 | KP088486 | KP088969 | |
| 43 | Berberidaceae | *Berberis* sp. | BOP010073 | KP088484 | KP088967 | |
| 44 | Berberidaceae | *Berberis* sp. | BOP010076 | KP088487 | KP088970 | |
| 45 | Berberidaceae | *Berberis* sp. | BOP010077 | KP088482 | KP088965 | |
| 46 | Berberidaceae | *Berberis* sp. | BOP010079 | KP088490 | KP088973 | |
| 47 | Berberidaceae | *Berberis* sp. | BOP010080 | KP088489 | KP088972 | |
| 48 | Berberidaceae | *Berberis* sp. | BOP010081 | KP088483 | KP088966 | |
| 49 | Berberidaceae | *Berberis* sp. | BOP010083 | KP088485 | KP088968 | |
| 50 | Berberidaceae | *Berberis* sp. | BOP010084 | KP088488 | KP088971 | |
| 51 | Berberidaceae | *Berberis thunbergii* | BOP010068 | KP088491 | KP088974 | |
| 52 | Berberidaceae | *Berberis thunbergii* | BOP010136 | KP088492 | KP088975 | |
| 53 | Berberidaceae | *Nandina domestica* | BOP010491 | KP088699 | | KP088413 |
| 54 | Betulaceae | *Betula alnoides* | BOP010014 | KP088493 | KP088976 | KP088084 |

| | | | | | | |
|---|---|---|---|---|---|---|
| 55 | Betulaceae | *Carpinus* sp. | BOP010436 | KP088508 | KP088991 | KP088379 |
| 56 | Betulaceae | *Carpinus turczaninowii* | BOP010434 | KP088509 | KP088992 | KP088377 |
| 57 | Betulaceae | *Corylus heterophylla* | BOP010330 | KP088546 | KP089024 | KP088330 |
| 58 | Bignoniaceae | *Campsis grandiflora* | BOP010266 | KP088506 | KP088989 | |
| 59 | Bignoniaceae | *Catalpa bungei* | BOP010225 | KP088515 | KP088996 | KP088246 |
| 60 | Bignoniaceae | *Catalpa ovata* | BOP010238 | KP088516 | KP088997 | KP088258 |
| 61 | Bignoniaceae | *Catalpa ovata* | BOP010458 | KP088517 | KP088998 | KP088394 |
| 62 | Bignoniaceae | *Catalpa* sp. | BOP010227 | KP088518 | KP088999 | KP088248 |
| 63 | Bombacaceae | *Bombax ceiba* | BOP010555 | KP088494 | KP088977 | KP088427 |
| 64 | Buxaceae | *Buxus sinica* var. *parvifolia* | BOP010025 | KP088499 | KP088982 | KP088095 |
| 65 | Buxaceae | *Buxus sinica* var. *parvifolia* | BOP010223 | KP088500 | KP088983 | KP088244 |
| 66 | Buxaceae | *Pachysandra terminalis* | BOP010463 | KP088701 | KP089166 | KP088398 |
| 67 | Calycanthaceae | *Calycanthus chinensis* | BOP010108 | KP088505 | KP088988 | KP088153 |
| 68 | Caprifoliaceae | *Abelia chinensis* | BOP010416 | KP088444 | KP088930 | |
| 69 | Caprifoliaceae | *Kolkwitzia amabilis* | BOP010103 | KP088645 | KP089118 | |
| 70 | Caprifoliaceae | *Lonicera × tellmanniana* | BOP010448 | KP088671 | KP089140 | |
| 71 | Caprifoliaceae | *Lonicera ferdinandii* | BOP010123 | KP088660 | KP089133 | |
| 72 | Caprifoliaceae | *Lonicera fragrantissima* | BOP010122 | KP088661 | KP089134 | |
| 73 | Caprifoliaceae | *Lonicera fragrantissima* | BOP010287 | KP088662 | | |
| 74 | Caprifoliaceae | *Lonicera japonica* | BOP010449 | KP088664 | KP089136 | |
| 75 | Caprifoliaceae | *Lonicera japonica* | BOP010152 | KP088663 | KP089135 | |
| 76 | Caprifoliaceae | *Lonicera maackii* | BOP010053 | KP088665 | KP089137 | |
| 77 | Caprifoliaceae | *Lonicera microphylla* | BOP010291 | KP088666 | | |
| 78 | Caprifoliaceae | *Lonicera* sp. | BOP010288 | KP088667 | | |
| 79 | Caprifoliaceae | *Lonicera tatarica* | BOP010283 | KP088669 | KP089138 | |
| 80 | Caprifoliaceae | *Lonicera tatarica* | BOP010292 | KP088670 | KP089139 | |
| 81 | Caprifoliaceae | *Lonicera tatarica* | BOP010281 | KP088668 | | |
| 82 | Caprifoliaceae | *Loricera korolkowii* | BOP010294 | KP088672 | KP089141 | |

| | | | | | | | |
|---|---|---|---|---|---|---|---|
| 83 | Caprifoliaceae | *Sambucus canaclensis* | BOP010269 | KP088849 | KP089308 | KP088285 |
| 84 | Caprifoliaceae | *Sambucus williamsii* | BOP010261 | KP088850 | KP089309 | KP088278 |
| 85 | Caprifoliaceae | *Viburnum burejaeticum* | BOP010262 | KP088903 | KP089357 | KP088279 |
| 86 | Caprifoliaceae | *Viburnum carlesii* | BOP010257 | KP088904 | KP089358 | KP088274 |
| 87 | Caprifoliaceae | *Viburnum dentatum* | BOP010259 | KP088905 | KP089359 | KP088276 |
| 88 | Caprifoliaceae | *Viburnum dilatatum* | BOP010158 | KP088906 | KP089360 | KP088191 |
| 89 | Caprifoliaceae | *Viburnum farreri* | BOP010166 | KP088907 | KP089361 | KP088198 |
| 90 | Caprifoliaceae | *Viburnum melanocarpum* | BOP010260 | KP088908 | KP089362 | KP088277 |
| 91 | Caprifoliaceae | *Viburnum mongolicum* | BOP010251 | KP088909 | KP089363 | KP088269 |
| 92 | Caprifoliaceae | *Viburnum opulus var. calvescens* | BOP010245 | KP088910 | KP089364 | KP088264 |
| 93 | Caprifoliaceae | *Viburnum prunifolium* | BOP010254 | KP088911 | KP089365 | KP088272 |
| 94 | Caprifoliaceae | *Viburnum rhytidophyllum* | BOP010258 | KP088912 | KP089366 | KP088275 |
| 95 | Caprifoliaceae | *Viburnum* sp. | BOP010246 | KP088914 | KP089368 | KP088265 |
| 96 | Caprifoliaceae | *Viburnum* sp. | BOP010247 | KP088915 | KP089369 | KP088266 |
| 97 | Caprifoliaceae | *Viburnum* sp. | BOP010255 | KP088913 | KP089367 | KP088273 |
| 98 | Caprifoliaceae | *Weigela florida* | BOP010212 | KP088919 | KP089372 | |
| 99 | Caprifoliaceae | *Weigela florida* | BOP010230 | KP088920 | | |
| 100 | Caprifoliaceae | *Weigela florida* | BOP010248 | KP088921 | KP089373 | |
| 101 | Caprifoliaceae | *Zabelia biflora* | BOP010125 | KP088924 | KP089376 | |
| 102 | Casuarinaceae | *Casuarina equisetifolia* | BOP010492 | KP088514 | | |
| 103 | Celastraceae | *Euongmus japonica* | BOP010131 | KP088586 | KP089060 | KP088172 |
| 104 | Celastraceae | *Euongmus kiautschovicus* | BOP010252 | KP088587 | KP089061 | KP088270 |
| 105 | Celastraceae | *Euonymus alatus* | BOP010126 | KP088588 | KP089062 | KP088167 |
| 106 | Celastraceae | *Euonymus bungeanus* | BOP010129 | KP088589 | KP089063 | KP088170 |
| 107 | Celastraceae | *Euonymus fortunei* | BOP010087 | KP088590 | KP089064 | KP088136 |
| 108 | Celastraceae | *Euonymus* sp. | BOP010127 | KP088591 | KP089065 | KP088168 |
| 109 | Celastraceae | *Euonymus* sp. | BOP010128 | KP088592 | KP089066 | KP088169 |
| 110 | Celastraceae | *Euonymus* sp. | BOP010253 | KP088593 | KP089067 | KP088271 |

| | | | | | | |
|---|---|---|---|---|---|---|
| 111 | Cephalotaxaceae | *Cephalotaxus sinensis* | BOP010413 | KP088524 | KP089001 | KP089424 |
| 112 | Cercidiphyllaceae | *Cercidiphyllum japonicum* | BOP010113 | | | KP088158 |
| 113 | Cornaceae | *Cornus alba* | BOP010093 | KP088541 | KP089019 | KP088443 |
| 114 | Cornaceae | *Cornus controversa* | BOP010289 | KP088542 | KP089020 | KP088439 |
| 115 | Cornaceae | *Cornus walteri* | BOP010312 | KP088543 | KP089021 | KP088440 |
| 116 | Cornaceae | *Cornus walteri* | BOP010313 | KP088544 | KP089022 | KP088441 |
| 117 | Cornaceae | *Cornus walteri* | BOP010446 | KP088545 | KP089023 | KP088442 |
| 118 | Cornaceae | *Dendrobenthamia japonica* var. *chinensis* | BOP010164 | KP088565 | KP089043 | KP088196 |
| 119 | Cornaceae | *Dendrobenthamia japonica* var. *chinensis* | BOP010315 | KP088566 | KP089044 | KP088316 |
| 120 | Cornaceae | *Dendrobenthamia japonica* var. *chinensis* | BOP010316 | KP088567 | KP089045 | KP088317 |
| 121 | Cornaceae | *Macrocarpium officinalis* | BOP010048 | KP088676 | KP089145 | KP088115 |
| 122 | Cucurbitaceae | *Trichosanthes* sp. | BOP010533 | KP088892 | KP089349 | KP088423 |
| 123 | Cupressaceae | *Chamaecyparis pisifera* | BOP010370 | KP088535 | KP089014 | KP089387 |
| 124 | Cupressaceae | *Juniperus procumbens* | BOP010361 | KP088638 | KP089111 | |
| 125 | Cupressaceae | *Juniperus virginiana* | BOP010369 | KP088534 | KP089013 | |
| 126 | Cupressaceae | *Juniperus virginiana* | BOP010381 | KP088640 | KP089113 | |
| 127 | Cupressaceae | *Platycladus orientalis* | BOP010371 | KP088750 | KP089210 | KP089388 |
| 128 | Cupressaceae | *Platycladus* sp. | BOP010375 | KP088749 | KP089209 | KP089390 |
| 129 | Cupressaceae | *Sabina chinensis* | BOP010372 | KP088843 | KP089302 | |
| 130 | Cupressaceae | *Thuja occidentalis* | BOP010373 | KP088880 | KP089337 | KP089419 |
| 131 | Ebenaceae | *Diospyros cathayensis* | BOP010150 | KP088569 | KP089047 | KP088185 |
| 132 | Ebenaceae | *Diospyros cathayensis* | BOP010151 | KP088570 | KP089048 | KP088186 |
| 133 | Ebenaceae | *Diospyros cathayensis* | BOP010244 | KP088571 | | KP088263 |
| 134 | Ebenaceae | *Diospyros kaki* | BOP010327 | KP088572 | KP089049 | KP088327 |
| 135 | Ebenaceae | *Diospyros lotus* | BOP010186 | KP088573 | | KP088215 |
| 136 | Ebenaceae | *Diospyros virginiana* | BOP010243 | KP088574 | | KP088262 |
| 137 | Elaeagnaceae | *Elaeagnus angustifolia* | BOP010326 | KP088577 | KP089052 | KP088326 |
| 138 | Elaeagnaceae | *Elaeagnus* sp. | BOP010119 | KP088578 | KP089053 | KP088164 |

| | | | | | | |
|---|---|---|---|---|---|---|
| 139 | Elaeagnaceae | *Elaeagnus umbellata* | BOP010319 | KP088579 | KP089054 | KP088320 |
| 140 | Elaeagnaceae | *Elaeagnus umbellata* | BOP010320 | KP088580 | KP089055 | |
| 141 | Ephedraceae | *Ephedra equisetina* | BOP010404 | KP088584 | | |
| 142 | Eucommiaceae | *Eucommia ulmoides* | BOP010485 | KP088585 | KP089059 | KP088409 |
| 143 | Euphorbiaceae | *Flueggea suffruticosa* | BOP010052 | KP088600 | KP089074 | KP088118 |
| 144 | Eupteleaceae | *Euptelea pleiospermum* | BOP010109 | KP088594 | KP089068 | KP088154 |
| 145 | Fabaceae | *Albizia julibrissin* | BOP010324 | KP088470 | KP088954 | KP088324 |
| 146 | Fabaceae | *Amorpha fruticosa* | BOP010050 | KP088472 | KP088955 | KP088117 |
| 147 | Fabaceae | *Caragana frutex* | BOP010063 | KP088507 | KP088990 | |
| 148 | Fabaceae | *Cercis canadensis* | BOP010060 | KP088526 | | KP088124 |
| 149 | Fabaceae | *Cercis chinensis* | BOP010459 | KP088527 | KP089007 | KP088395 |
| 150 | Fabaceae | *Cercis gigantea* | BOP010059 | KP088528 | | KP088123 |
| 151 | Fabaceae | *Coluten arborescens* | BOP010270 | KP088540 | KP089018 | |
| 152 | Fabaceae | *Gleditsia sinensis* | BOP010054 | KP088622 | KP089096 | |
| 153 | Fabaceae | *Gleditsia triacanthos* | BOP010444 | KP088623 | KP089097 | |
| 154 | Fabaceae | *Halimodendron halodendron* | BOP010290 | KP088627 | KP089101 | |
| 155 | Fabaceae | *Indigofera kirilowii* | BOP010061 | KP088633 | KP089107 | KP088125 |
| 156 | Fabaceae | *Pueraria* sp. | BOP010161 | KP088799 | | |
| 157 | Fabaceae | *Sophora davidii* | BOP010062 | KP088853 | KP089311 | KP088126 |
| 158 | Fabaceae | *Sophora japonica* | BOP010088 | KP088854 | KP089312 | KP088137 |
| 159 | Fabaceae | *Sophora viciifolia* | BOP010272 | KP088855 | KP089313 | KP088287 |
| 160 | Fabaceae | *Wisteria floribunda* | BOP010102 | KP088922 | KP089374 | |
| 161 | Fagaceae | *Castanea mollissima* | BOP010328 | KP088513 | | KP088328 |
| 162 | Fagaceae | *Quercus aliena* | BOP010439 | KP088805 | KP089264 | KP088382 |
| 163 | Fagaceae | *Quercus aliena* | BOP010423 | KP088804 | KP089263 | KP088366 |
| 164 | Fagaceae | *Quercus aliena* var.*acuteserrata* | BOP010421 | KP088801 | KP089260 | KP088364 |
| 165 | Fagaceae | *Quercus aliena* var.*acuteserrata* | BOP010422 | KP088802 | KP089261 | KP088365 |
| 166 | Fagaceae | *Quercus aliena* var.*acuteserrata* | BOP010442 | KP088803 | KP089262 | KP088385 |

| | | | | | | |
|---|---|---|---|---|---|---|
| 167 | Fagaceae | *Quercus baronii* | BOP010441 | KP088806 | KP089265 | KP088384 |
| 168 | Fagaceae | *Quercus dentata* | BOP010271 | KP088807 | KP089266 | KP088286 |
| 169 | Fagaceae | *Quercus fabri* | BOP010420 | KP088808 | KP089267 | KP088363 |
| 170 | Fagaceae | *Quercus fabri* | BOP010426 | KP088809 | KP089268 | KP088369 |
| 171 | Fagaceae | *Quercus gambelii* | BOP010431 | KP088810 | KP089269 | KP088374 |
| 172 | Fagaceae | *Quercus glandulifera* var. *brevipetiolata* | BOP010427 | KP088811 | KP089270 | KP088370 |
| 173 | Fagaceae | *Quercus macrocalyx* | BOP010443 | | KP089271 | |
| 174 | Fagaceae | *Quercus macrocarpa* | BOP010428 | KP088812 | KP089272 | KP088371 |
| 175 | Fagaceae | *Quercus palustris* | BOP010429 | KP088813 | KP089273 | KP088372 |
| 176 | Fagaceae | *Quercus robur* | BOP010418 | KP088814 | KP089274 | KP088361 |
| 177 | Fagaceae | *Quercus robur* | BOP010419 | KP088815 | KP089275 | KP088362 |
| 178 | Fagaceae | *Quercus rubra* | BOP010433 | KP088816 | KP089276 | KP088376 |
| 179 | Fagaceae | *Quercus serrata* | BOP010440 | KP088817 | KP089277 | KP088383 |
| 180 | Fagaceae | *Quercus* sp. | BOP010417 | KP088818 | KP089278 | KP088360 |
| 181 | Fagaceae | *Quercus* sp. | BOP010425 | KP088819 | KP089279 | KP088368 |
| 182 | Fagaceae | *Quercus* sp. | BOP010430 | KP088820 | KP089280 | KP088373 |
| 183 | Fagaceae | *Quercus stellata* | BOP010424 | KP088821 | KP089281 | KP088367 |
| 184 | Fagaceae | *Quercus variabilis* | BOP010445 | KP088822 | KP089282 | KP088386 |
| 185 | Ginkgoaceae | *Ginkgo biloba* | BOP010383 | KP088621 | KP089095 | |
| 186 | Hamamelidaceae | *Hamamelis mollis* | BOP010148 | | | KP088183 |
| 187 | Hippocastanaceae | *Aesculus chinensis* | BOP010140 | KP088461 | KP088946 | KP088178 |
| 188 | Hippocastanaceae | *Aesculus chinensis* | BOP010250 | KP088462 | KP088947 | KP088268 |
| 189 | Hippocastanaceae | *Aesculus* sp. | BOP010155 | KP088463 | KP088948 | KP088188 |
| 190 | Hippocastanaceae | *Aesculus turbinata* | BOP010249 | KP088464 | KP088949 | KP088267 |
| 191 | Hippocastanaceae | *Aesculus wangii* | BOP010114 | KP088465 | KP088950 | KP088159 |
| 192 | Hydrangeaceae | *Deutzia parviflora* | BOP010045 | KP088568 | KP089046 | KP088113 |
| 193 | Hydrangeaceae | *Hydrangea macrophylla* f. *otaksa* | BOP010452 | KP088632 | KP089106 | KP088388 |
| 194 | Hydrangeaceae | *Philadelphus coronarius* | BOP010219 | KP088708 | KP089170 | KP088434 |

| | | | | | | | |
|---|---|---|---|---|---|---|---|
| 195 | Hydrangeaceae | *Philadelphus lemoiner* | BOP010218 | KP088709 | KP089171 | KP088433 |
| 196 | Hydrangeaceae | *Philadelphus pekinensis* | BOP010046 | KP088710 | KP089172 | KP088432 |
| 197 | Hydrangeaceae | *Philadelphus pekinensis* | BOP010220 | KP088711 | KP089173 | KP088435 |
| 198 | Hydrangeaceae | *Philadelphus pekinensis* | BOP010221 | KP088712 | KP089174 | KP088436 |
| 199 | Hydrangeaceae | *Philadelphus pekinensis* | BOP010222 | KP088713 | KP089175 | KP088437 |
| 200 | Hydrangeaceae | *Philadelphus pekinensis* | BOP010465 | KP088714 | KP089176 | KP088438 |
| 201 | Juglandaceae | *Carya cathayensis* | BOP010299 | KP088510 | KP088993 | KP088302 |
| 202 | Juglandaceae | *Carya illinoensis* | BOP010378 | KP088511 | KP088994 | KP088357 |
| 203 | Juglandaceae | *Cyclocarya paliurus* | BOP010527 | KP088561 | KP089039 | KP088421 |
| 204 | Juglandaceae | *Juglans mandshurica* | BOP010116 | KP088635 | KP089108 | KP088161 |
| 205 | Juglandaceae | *Juglans regia* | BOP010117 | KP088636 | KP089109 | KP088162 |
| 206 | Juglandaceae | *Juglans* sp. | BOP010348 | KP088637 | KP089110 | KP088346 |
| 207 | Juglandaceae | *Pterocarya* sp. | BOP010297 | KP088797 | KP089254 | KP088301 |
| 208 | Juglandaceae | *Pterocarya stenoptera* | BOP010296 | KP088798 | KP089255 | KP088300 |
| 209 | Lamiaceae | *Elsholtzia stauntoni* | BOP010179 | KP088583 | KP089058 | KP088208 |
| 210 | Lardizabalaceae | *Akebia trifoliata* | BOP010268 | KP088467 | KP088951 | KP088284 |
| 211 | Lauraceae | *Laurus nobilis* | BOP010497 | KP088651 | KP089124 | KP088417 |
| 212 | Lauraceae | *Lindera glauca* | BOP010154 | KP088658 | KP089131 | KP088187 |
| 213 | Loganiaceae | *Buddleja albiflora* | BOP010282 | KP088497 | KP088980 | KP088296 |
| 214 | Lythraceae | *Lagerstroemia indica* | BOP010171 | KP088646 | KP089119 | KP088202 |
| 215 | Magnoliaceae | *Liriodendron chinense* | BOP010099 | KP088659 | KP089132 | KP088147 |
| 216 | Magnoliaceae | *Magnolia* × *soulangeana* | BOP010455 | KP088680 | | KP088391 |
| 217 | Magnoliaceae | *Magnolia amoena* | BOP010098 | KP088677 | KP089146 | KP088146 |
| 218 | Magnoliaceae | *Magnolia bionlii* | BOP010156 | KP088678 | KP089147 | KP088189 |
| 219 | Magnoliaceae | *Magnolia cylindrica* | BOP010104 | KP088679 | KP089148 | KP088150 |
| 220 | Magnoliaceae | *Magnolia* sp. | BOP010092 | KP088681 | KP089149 | KP088141 |
| 221 | Magnoliaceae | *Magnolia zenii* | BOP010100 | KP088682 | KP089150 | KP088148 |
| 222 | Malvaceae | *Grewia biloba* | BOP010214 | KP088625 | KP089099 | KP088240 |

| | | | | | | |
|---|---|---|---|---|---|---|
| 223 | Malvaceae | *Grewia biloba* | BOP010476 | KP088626 | KP089100 | KP088404 |
| 224 | Malvaceae | *Hibiscus syriacus* | BOP010090 | KP088630 | KP089104 | KP088139 |
| 225 | Meliaceae | *Melia azedarach* | BOP010460 | KP088694 | KP089161 | KP088396 |
| 226 | Meliaceae | *Toona sinensis* | BOP010473 | KP088890 | | |
| 227 | Moraceae | *Broussonetia papyifera* | BOP010317 | KP088496 | KP088979 | KP088318 |
| 228 | Moraceae | *Ficus carica* | BOP010474 | KP088598 | KP089072 | KP088402 |
| 229 | Moraceae | *Ficus religiosa* | BOP010493 | KP088599 | KP089073 | KP088414 |
| 230 | Moraceae | *Maclura pomifera* | BOP010191 | KP088674 | KP089143 | KP088220 |
| 231 | Moraceae | *Maclura tricuspidata* | BOP010167 | KP088675 | KP089144 | KP088199 |
| 232 | Moraceae | *Morus alba* | BOP010318 | KP088697 | KP089164 | KP088319 |
| 233 | Myrtaceae | *Syzggiam samaragense* | BOP010488 | KP088873 | KP089330 | KP088411 |
| 234 | Nyctaginaceae | *Bougainvillea spectabilis* | BOP010543 | KP088495 | KP088978 | KP088431 |
| 235 | Oleaceae | *Chionanthus retusus* | BOP010231 | KP088536 | KP089015 | KP088251 |
| 236 | Oleaceae | *Fontanesia fortunei* | BOP010121 | KP088601 | KP089075 | KP088166 |
| 237 | Oleaceae | *Fontanesia phillyreoides* subsp. *fortunei* | BOP010056 | KP088602 | KP089076 | KP088120 |
| 238 | Oleaceae | *Forsythia ovata* | BOP010275 | KP088603 | KP089077 | KP088290 |
| 239 | Oleaceae | *Forsythia suspensa* | BOP010094 | KP088604 | KP089078 | KP088142 |
| 240 | Oleaceae | *Forsythia suspensa* | BOP010274 | KP088605 | KP089079 | KP088289 |
| 241 | Oleaceae | *Forsythia viridissima* | BOP010273 | KP088606 | KP089080 | KP088288 |
| 242 | Oleaceae | *Fraxinus americana* | BOP010237 | KP088608 | KP089082 | KP088257 |
| 243 | Oleaceae | *Fraxinus baroniana* | BOP010224 | KP088609 | KP089083 | KP088245 |
| 244 | Oleaceae | *Fraxinus bungeana* | BOP010229 | KP088610 | KP089084 | KP088250 |
| 245 | Oleaceae | *Fraxinus excelsior* var. *aurea* | BOP010235 | KP088611 | KP089085 | KP088255 |
| 246 | Oleaceae | *Fraxinus hubeiensis* | BOP010236 | KP088612 | KP089086 | KP088256 |
| 247 | Oleaceae | *Fraxinus mandshurica* | BOP010120 | KP088613 | KP089087 | KP088165 |
| 248 | Oleaceae | *Fraxinus paxiana* | BOP010085 | KP088614 | KP089088 | KP088134 |
| 249 | Oleaceae | *Fraxinus pennsylvanica* | BOP010233 | KP088616 | KP089090 | KP088253 |
| 250 | Oleaceae | *Fraxinus pennsylvanica* var. *subintegerrima* | BOP010241 | KP088615 | KP089089 | KP088260 |

| | | | | | | |
|---|---|---|---|---|---|---|
| 251 | Oleaceae | *Fraxinus rhynchophylla* | BOP010232 | KP088617 | KP089091 | KP088252 |
| 252 | Oleaceae | *Fraxinus* sp. | BOP010228 | KP088619 | KP089093 | KP088249 |
| 253 | Oleaceae | *Fraxinus* sp. | BOP010234 | KP088618 | KP089092 | KP088254 |
| 254 | Oleaceae | *Fraxinus velutina* | BOP010226 | KP088620 | KP089094 | KP088247 |
| 255 | Oleaceae | *Ligustrum* × *vicaryi* | BOP010278 | KP088657 | KP089130 | KP088293 |
| 256 | Oleaceae | *Ligustrum lucidum* | BOP010075 | KP088653 | KP089126 | KP088133 |
| 257 | Oleaceae | *Ligustrum quihoui* | BOP010314 | KP088654 | KP089127 | KP088315 |
| 258 | Oleaceae | *Ligustrum sinense* | BOP010279 | KP088655 | KP089128 | KP088294 |
| 259 | Oleaceae | *Ligustrum* sp. | BOP010277 | KP088656 | KP089129 | KP088292 |
| 260 | Oleaceae | *Syringa oblata* | BOP010004 | KP088867 | KP089324 | KP088074 |
| 261 | Oleaceae | *Syringa oblata* | BOP010464 | KP088869 | KP089326 | KP088399 |
| 262 | Oleaceae | *Syringa oblata* | BOP010005 | KP088868 | KP089325 | KP088075 |
| 263 | Oleaceae | *Syringa pekinensis* | BOP010002 | KP088870 | KP089327 | KP088072 |
| 264 | Oleaceae | *Syringa reticulata* subsp. *amurensis* | BOP010153 | KP088871 | KP089328 | |
| 265 | Oleaceae | *Syringa wolfii* | BOP010003 | KP088872 | KP089329 | KP088073 |
| 266 | Pandanaceae | *Pandanus veitchii* | BOP010496 | KP088702 | | KP088416 |
| 267 | Pinaceae | *Abies firma* | BOP010382 | KP088445 | KP088931 | KP089394 |
| 268 | Pinaceae | *Abies holophylla* | BOP010412 | KP088446 | KP088932 | KP089416 |
| 269 | Pinaceae | *Abies nephrolepis* | BOP010411 | KP088447 | KP088933 | KP089415 |
| 270 | Pinaceae | *Cedrus deodara* | BOP010415 | KP088519 | KP089000 | KP089417 |
| 271 | Pinaceae | *Juniperus rigida* | BOP010380 | KP088639 | KP089112 | |
| 272 | Pinaceae | *Larix gmelinii* | BOP010368 | KP088648 | KP089121 | KP089386 |
| 273 | Pinaceae | *Larix gmelinii* | BOP010407 | KP088649 | KP089122 | KP089412 |
| 274 | Pinaceae | *Larix gmelinii* var. *olgensis* | BOP010408 | KP088647 | KP089120 | KP089413 |
| 275 | Pinaceae | *Larix kaempferi* | BOP010395 | KP088650 | KP089123 | KP089405 |
| 276 | Pinaceae | *Picea asperata* | BOP010388 | KP088718 | KP089178 | KP089398 |
| 277 | Pinaceae | *Picea crassifolia* | BOP010386 | KP088719 | KP089179 | KP089396 |
| 278 | Pinaceae | *Picea koraiensis* | BOP010389 | KP088720 | KP089180 | KP089399 |

| | | | | | | |
|---|---|---|---|---|---|---|
| 279 | Pinaceae | *Picea meyeri* | BOP010393 | KP088721 | KP089181 | KP089403 |
| 280 | Pinaceae | *Picea polita* | BOP010392 | KP088722 | KP089182 | KP089402 |
| 281 | Pinaceae | *Picea schrenkiana* | BOP010387 | KP088723 | KP089183 | KP089397 |
| 282 | Pinaceae | *Picea* sp. | BOP010364 | KP088726 | KP089186 | KP089382 |
| 283 | Pinaceae | *Picea* sp. | BOP010365 | KP088725 | KP089185 | KP089383 |
| 284 | Pinaceae | *Picea* sp. | BOP010366 | KP088727 | KP089187 | KP089384 |
| 285 | Pinaceae | *Picea* sp. | BOP010391 | KP088724 | KP089184 | KP089401 |
| 286 | Pinaceae | *Picea wilsonii* | BOP010390 | KP088728 | KP089188 | KP089400 |
| 287 | Pinaceae | *Pinus armandii* | BOP010394 | KP088729 | KP089189 | KP089404 |
| 288 | Pinaceae | *Pinus banksiana* | BOP010398 | KP088730 | KP089190 | KP089408 |
| 289 | Pinaceae | *Pinus bungeana* | BOP010376 | KP088731 | KP089191 | KP089391 |
| 290 | Pinaceae | *Pinus densifora* | BOP010367 | KP088732 | KP089192 | KP089385 |
| 291 | Pinaceae | *Pinus griffithii* | BOP010379 | KP088733 | KP089193 | KP089393 |
| 292 | Pinaceae | *Pinus koraiensis* | BOP010403 | KP088734 | KP089194 | KP089410 |
| 293 | Pinaceae | *Pinus nigra* | BOP010406 | KP088735 | KP089195 | KP089411 |
| 294 | Pinaceae | *Pinus ponderosa* | BOP010377 | KP088736 | KP089196 | KP089392 |
| 295 | Pinaceae | *Pinus* sp. | BOP010396 | KP088737 | KP089197 | KP089406 |
| 296 | Pinaceae | *Pinus* sp. | BOP010400 | KP088738 | KP089198 | KP089409 |
| 297 | Pinaceae | *Pinus* sp. | BOP010480 | KP088739 | KP089199 | KP089418 |
| 298 | Pinaceae | *Pinus strobus* | BOP010374 | KP088740 | KP089200 | KP089389 |
| 299 | Pinaceae | *Pinus sylvestris* var. *mongholica* | BOP010409 | KP088741 | KP089201 | KP089414 |
| 300 | Pinaceae | *Pinus sylvestris* var.*sylvestriformis* | BOP010397 | KP088742 | KP089202 | KP089407 |
| 301 | Pinaceae | *Pinus tabulaeformis* | BOP010362 | KP088743 | KP089203 | KP089380 |
| 302 | Pinaceae | *Pinus thunbergii* | BOP010363 | KP088744 | KP089204 | KP089381 |
| 303 | Pinaceae | *Pinus thunbergii* | BOP010384 | KP088745 | KP089205 | KP089395 |
| 304 | Pinaceae | *Pseudotsuga menziesii* | BOP010481 | KP088796 | KP089253 | |
| 305 | Pittosporaceae | *Pittosporum* sp. | BOP010559 | KP088747 | KP089207 | KP088429 |
| 306 | Platanaceae | *Platanus orientalis* | BOP010462 | KP088748 | KP089208 | |

| | | | | | | |
|---|---|---|---|---|---|---|
| 307 | Poaceae | *Fargesia* sp. | BOP010468 | KP088597 | KP089071 | |
| 308 | Poaceae | *Indocalamus tessellatus* | BOP010499 | KP088634 | | |
| 309 | Poaceae | *Phyllostachys nigra var. henonis* | BOP010498 | KP088716 | | |
| 310 | Polemoniaceae | *Phlox longifolia* | BOP010512 | KP088715 | | KP088420 |
| 311 | Profeaceae | *Grevillea robusta* | BOP010494 | KP088624 | KP089098 | KP088415 |
| 312 | Punicaceae | *Punica granatum* | BOP010501 | KP088800 | KP089256 | KP088419 |
| 313 | Rhamnaceae | *Hovenia acerba* | BOP010183 | KP088631 | KP089105 | KP088212 |
| 314 | Rhamnaceae | *Rhamnus davurica* | BOP010172 | KP088823 | KP089283 | KP088203 |
| 315 | Rhamnaceae | *Rhamnus davurica* | BOP010181 | KP088824 | KP089284 | KP088210 |
| 316 | Rhamnaceae | *Rhamnus davurica* | BOP010193 | KP088825 | KP089285 | KP088222 |
| 317 | Rhamnaceae | *Rhamnus globosa* | BOP010001 | KP088826 | KP089286 | KP088071 |
| 318 | Rhamnaceae | *Rhamnus utilis* | BOP010182 | KP088827 | KP089287 | KP088211 |
| 319 | Rhamnaceae | *Sageretia paucicostata* | BOP010189 | KP088844 | KP089303 | KP088218 |
| 320 | Rhamnaceae | *Ziziphus jujuba* | BOP010132 | KP088929 | KP089379 | KP088173 |
| 321 | Ribesiaceae | *Ribes* sp. | BOP010049 | KP088834 | KP089294 | KP088116 |
| 322 | Ribesiaceae | *Ribes* sp. | BOP010190 | KP088835 | KP089295 | KP088219 |
| 323 | Ribesiaceae | *Ribes* sp. | BOP010192 | KP088836 | KP089296 | KP088221 |
| 324 | Rosaceae | *Aria* sp. | BOP010044 | KP088476 | KP088959 | KP088112 |
| 325 | Rosaceae | *Cerasus yedoensis* | BOP010038 | KP088525 | KP089006 | KP088106 |
| 326 | Rosaceae | *Chaenomeles sinensis* | BOP010349 | KP088529 | KP089008 | KP088347 |
| 327 | Rosaceae | *Chaenomeles* sp. | BOP010031 | KP088531 | KP089010 | KP088101 |
| 328 | Rosaceae | *Chaenomeles speciosa* | BOP010026 | KP088532 | KP089011 | KP088096 |
| 329 | Rosaceae | *Chaenomeles speciosa* | BOP010027 | KP088533 | KP089012 | KP088097 |
| 330 | Rosaceae | *Chaenomeles speciosa* | BOP010159 | KP088530 | KP089009 | KP088192 |
| 331 | Rosaceae | *Cotoneaster microphyllus* | BOP010036 | KP088548 | KP089026 | KP088104 |
| 332 | Rosaceae | *Cotoneaster multiflorus* | BOP010016 | KP088549 | KP089027 | KP088086 |
| 333 | Rosaceae | *Cotoneaster* sp. | BOP010023 | KP088550 | KP089028 | KP088093 |
| 334 | Rosaceae | *Cotoneaster* sp. | BOP010029 | KP088551 | KP089029 | KP088099 |

| | | | | | | |
|---|---|---|---|---|---|---|
| 335 | Rosaceae | *Cotoneaster* sp. | BOP010030 | KP088552 | KP089030 | KP088100 |
| 336 | Rosaceae | *Cotoneaster submultiflorus* | BOP010185 | KP088553 | KP089031 | KP088214 |
| 337 | Rosaceae | *Crataegus kansuensis* | BOP010010 | KP088554 | KP089032 | KP088080 |
| 338 | Rosaceae | *Crataegus pinnatifida* | BOP010344 | KP088555 | KP089033 | KP088342 |
| 339 | Rosaceae | *Crataegus pinnatifida* | BOP010345 | KP088556 | KP089034 | KP088343 |
| 340 | Rosaceae | *Crataegus pinnatifida* | BOP010346 | KP088557 | KP089035 | KP088344 |
| 341 | Rosaceae | *Crataegus pinnatifida* | BOP010347 | KP088558 | KP089036 | KP088345 |
| 342 | Rosaceae | *Crataegus* sp. | BOP010018 | KP088559 | KP089037 | KP088088 |
| 343 | Rosaceae | *Crataegus* sp. | BOP010478 | KP088560 | KP089038 | KP088405 |
| 344 | Rosaceae | *Cydonia oblanga* | BOP010020 | KP088562 | KP089040 | KP088090 |
| 345 | Rosaceae | *Cydonia* sp. | BOP010351 | KP088563 | KP089041 | KP088349 |
| 346 | Rosaceae | *Dasiphora* sp. | BOP010541 | KP088564 | KP089042 | KP088425 |
| 347 | Rosaceae | *Duchesnea indica* | BOP010165 | KP088575 | KP089050 | KP088197 |
| 348 | Rosaceae | *Exochorda racemosa* | BOP010047 | KP088595 | KP089069 | KP088114 |
| 349 | Rosaceae | *Exochorda* sp. | BOP010472 | KP088596 | KP089070 | |
| 350 | Rosaceae | *Fragaria ananassa* | BOP010562 | KP088607 | KP089081 | KP088430 |
| 351 | Rosaceae | *Kerria japonica* | BOP010028 | KP088642 | KP089115 | KP088098 |
| 352 | Rosaceae | *Malus halliana* | BOP010285 | KP088683 | KP089151 | KP088297 |
| 353 | Rosaceae | *Malus micromalus* | BOP010089 | KP088684 | KP089152 | KP088138 |
| 354 | Rosaceae | *Malus sieversii* | BOP010115 | KP088685 | KP089153 | KP088160 |
| 355 | Rosaceae | *Malus* sp. | BOP010032 | KP088686 | KP089154 | KP088102 |
| 356 | Rosaceae | *Malus* sp. | BOP010033 | KP088687 | KP089155 | KP088103 |
| 357 | Rosaceae | *Malus* sp. | BOP010354 | KP088688 | KP089156 | KP088351 |
| 358 | Rosaceae | *Malus* sp. | BOP010356 | KP088690 | KP089158 | KP088352 |
| 359 | Rosaceae | *Malus* sp. | BOP010357 | KP088689 | KP089157 | KP088353 |
| 360 | Rosaceae | *Malus* sp. | BOP010358 | KP088691 | KP089159 | KP088354 |
| 361 | Rosaceae | *Malus spectabilis* | BOP010021 | KP088692 | KP089160 | KP088091 |
| 362 | Rosaceae | *Mespilus* sp. | BOP010017 | KP088695 | KP089162 | KP088087 |

| | | | | | | |
|---|---|---|---|---|---|---|
| 363 | Rosaceae | *Physocarpus amuresis* | BOP010043 | KP088717 | KP089177 | KP088111 |
| 364 | Rosaceae | *Prinsepia sinensis* | BOP010133 | KP088760 | KP089220 | KP088174 |
| 365 | Rosaceae | *Prinsepia uniflora* | BOP010147 | KP088761 | KP089221 | KP088182 |
| 366 | Rosaceae | *Prunus cerasifera* f. *atropurpurea* | BOP010039 | KP088768 | KP089222 | KP088107 |
| 367 | Rosaceae | *Prunus davidiana* | BOP010009 | KP088769 | KP089223 | KP088079 |
| 368 | Rosaceae | *Prunus davidiana* | BOP010343 | KP088770 | KP089224 | KP088341 |
| 369 | Rosaceae | *Prunus glandulosa* | BOP010170 | KP088771 | KP089225 | KP088201 |
| 370 | Rosaceae | *Prunus glandulosa* | BOP010337 | KP088772 | KP089226 | KP088335 |
| 371 | Rosaceae | *Prunus japonica* | BOP010041 | KP088762 | KP089227 | KP088109 |
| 372 | Rosaceae | *Prunus japonica* | BOP010432 | KP088763 | KP089228 | KP088375 |
| 373 | Rosaceae | *Prunus padus* | BOP010012 | KP088764 | KP089229 | KP088082 |
| 374 | Rosaceae | *Prunus persica* | BOP010342 | KP088766 | KP089231 | KP088340 |
| 375 | Rosaceae | *Prunus persica* | BOP010265 | KP088765 | KP089230 | KP088282 |
| 376 | Rosaceae | *Prunus salicina* | BOP010335 | KP088767 | KP089232 | |
| 377 | Rosaceae | *Prunus sargentii* | BOP010500 | KP088773 | KP089233 | KP088418 |
| 378 | Rosaceae | *Prunus serrulata* | BOP010111 | KP088783 | KP089236 | KP088156 |
| 379 | Rosaceae | *Prunus serrulata* | BOP010333 | KP088784 | KP089237 | KP088332 |
| 380 | Rosaceae | *Prunus serrulata* | BOP010334 | KP088785 | KP089238 | KP088333 |
| 381 | Rosaceae | *Prunus serrulata* | BOP010338 | KP088786 | KP089239 | KP088336 |
| 382 | Rosaceae | *Prunus serrulata* | BOP010461 | KP088787 | KP089240 | KP088397 |
| 383 | Rosaceae | *Prunus serrulata* var. *lannesiana* | BOP010037 | KP088781 | KP089234 | KP088105 |
| 384 | Rosaceae | *Prunus serrulata* var. *lannesiana* | BOP010040 | KP088782 | KP089235 | KP088108 |
| 385 | Rosaceae | *Prunus sibirica* | BOP010006 | KP088788 | KP089241 | KP088076 |
| 386 | Rosaceae | *Prunus sibirica* | BOP010118 | KP088789 | KP089242 | KP088163 |
| 387 | Rosaceae | *Prunus* sp. | BOP010331 | KP088794 | KP089248 | KP088331 |
| 388 | Rosaceae | *Prunus* sp. | BOP010336 | KP088792 | KP089246 | KP088334 |
| 389 | Rosaceae | *Prunus* sp. | BOP010339 | KP088793 | KP089247 | KP088337 |
| 390 | Rosaceae | *Prunus* sp. | BOP010341 | KP088791 | KP089245 | KP088339 |

| | | | | | | |
|---|---|---|---|---|---|---|
| 391 | Rosaceae | *Prunus* sp. | BOP010477 | KP088780 | KP089243 | |
| 392 | Rosaceae | *Prunus* sp. | BOP010482 | KP088790 | KP089244 | KP088406 |
| 393 | Rosaceae | *Prunus subhirtella* var. *pedula* | BOP010112 | KP088774 | KP089249 | KP088157 |
| 394 | Rosaceae | *Prunus tangutica* | BOP010008 | KP088775 | KP089250 | KP088078 |
| 395 | Rosaceae | *Prunus triloba* | BOP010340 | KP088795 | KP089251 | KP088338 |
| 396 | Rosaceae | *Prunus ussuriensis* | BOP010329 | KP088776 | KP089252 | KP088329 |
| 397 | Rosaceae | *Pyrus betulifolia* | BOP010352 | KP088777 | KP089257 | KP088350 |
| 398 | Rosaceae | *Pyrus bretschneideri* | BOP010065 | KP088778 | KP089258 | KP088128 |
| 399 | Rosaceae | *Pyrus calleryana* | BOP010350 | KP088779 | KP089259 | KP088348 |
| 400 | Rosaceae | *Rhodotypos scandens* | BOP010022 | KP088828 | KP089288 | KP088092 |
| 401 | Rosaceae | *Rosa multiflora* | BOP010453 | KP088837 | KP089297 | KP088389 |
| 402 | Rosaceae | *Rosa rugosa* | BOP010536 | KP088838 | KP089298 | KP088424 |
| 403 | Rosaceae | *Rosa* sp. | BOP010157 | KP088840 | KP089299 | KP088190 |
| 404 | Rosaceae | *Rosa* sp. | BOP010267 | KP088839 | | KP088283 |
| 405 | Rosaceae | *Rosa xanthina* | BOP010007 | KP088841 | KP089300 | KP088077 |
| 406 | Rosaceae | *Rubus palmatus* | BOP010130 | KP088842 | KP089301 | KP088171 |
| 407 | Rosaceae | *Sorbaria sorbifolia* | BOP010264 | KP088856 | KP089314 | KP088281 |
| 408 | Rosaceae | *Spiraea chinensis* | BOP010437 | KP088857 | KP089315 | KP088380 |
| 409 | Rosaceae | *Spiraea nipponica 'snowmucond'* | BOP010240 | KP088858 | KP089316 | KP088259 |
| 410 | Rosaceae | *Spiraea pubesces* | BOP010042 | KP088859 | KP089317 | KP088110 |
| 411 | Rosaceae | *Spiraea salicifolia* | BOP010066 | KP088860 | KP089318 | KP088129 |
| 412 | Rosaceae | *Spiraea salicifolia* | BOP010071 | KP088861 | KP089319 | KP088130 |
| 413 | Rosaceae | *Spiraea salicifolia* | BOP010072 | KP088862 | KP089320 | KP088131 |
| 414 | Rosaceae | *Spiraea salicifolia* | BOP010174 | KP088863 | KP089321 | |
| 415 | Rosaceae | *Spiraea salicifolia* | BOP010438 | KP088864 | KP089322 | KP088381 |
| 416 | Rosaceae | *Spiraea* sp. | BOP010360 | KP088865 | KP089323 | KP088356 |
| 417 | Rubiaceae | *Leptodermis oblonga* | BOP010280 | KP088652 | KP089125 | KP088295 |
| 418 | Rutaceae | *Citrus maxima* | BOP010486 | KP088537 | | KP088410 |

| | | | | | | |
|---|---|---|---|---|---|---|
| 419 | Rutaceae | *Citrus trifoliata* | BOP010149 | KP088538 | KP089016 | KP088184 |
| 420 | Rutaceae | *Phellodendron chinense* | BOP010196 | KP088706 | KP089168 | KP088225 |
| 421 | Rutaceae | *Phellodendron* sp. | BOP010097 | KP088707 | KP089169 | KP088145 |
| 422 | Rutaceae | *Tetradium daniellii* | BOP010405 | KP088878 | KP089335 | KP088358 |
| 423 | Rutaceae | *Zanthoxylum bungeanum* | BOP010483 | KP088925 | KP089377 | KP088407 |
| 424 | Salicaceae | *Populus ×beijingensis* | BOP010177 | KP088757 | KP089217 | KP088206 |
| 425 | Salicaceae | *Populus ×canadensis* | BOP010175 | KP088758 | KP089218 | KP088204 |
| 426 | Salicaceae | *Populus ×canadensis* | BOP010178 | KP088759 | KP089219 | KP088207 |
| 427 | Salicaceae | *Populus alba* | BOP010184 | KP088752 | KP089212 | KP088213 |
| 428 | Salicaceae | *Populus alba var. pyramdalis* | BOP010169 | KP088751 | KP089211 | KP088200 |
| 429 | Salicaceae | *Populus candicans* | BOP010187 | KP088753 | KP089213 | KP088216 |
| 430 | Salicaceae | *Populus cathayana* | BOP010176 | KP088754 | KP089214 | KP088205 |
| 431 | Salicaceae | *Populus* sp. | BOP010024 | KP088755 | KP089215 | KP088094 |
| 432 | Salicaceae | *Populus* sp. | BOP010188 | KP088756 | KP089216 | KP088217 |
| 433 | Salicaceae | *Salix babylonica* | BOP010457 | KP088845 | KP089304 | KP088393 |
| 434 | Salicaceae | *Salix matsudana* | BOP010180 | KP088847 | KP089306 | KP088209 |
| 435 | Salicaceae | *Salix matsudana f. pendula* | BOP010137 | KP088846 | KP089305 | KP088176 |
| 436 | Salicaceae | *Salix* sp. | BOP010194 | KP088848 | KP089307 | KP088223 |
| 437 | Sapindaceae | *Koelreuteria paniculata* | BOP010051 | KP088643 | KP089116 | |
| 438 | Sapindaceae | *Koelreuteria paniculata* | BOP010197 | KP088644 | KP089117 | |
| 439 | Sapindaceae | *Xanthoceras sorbifolia* | BOP010107 | KP088923 | KP089375 | KP088152 |
| 440 | Sapotaceae | *Manilkara zapota* | BOP010544 | KP088693 | | KP088426 |
| 441 | Schisandraceae | *Schisandra chinensis* | BOP010144 | KP088851 | KP089310 | KP088180 |
| 442 | Scrophulariaceae | *Buddleja lindleyana* | BOP010309 | KP088498 | KP088981 | KP088312 |
| 443 | Scrophulariaceae | *Paulownia fortunei* | BOP010475 | KP088705 | | KP088403 |
| 444 | Simaroubaceae | *Ailanthus altissima* | BOP010321 | KP088466 | | KP088321 |
| 445 | Solanaceae | *Lycium barbarum* | BOP010138 | KP088673 | KP089142 | KP088177 |
| 446 | Styracaceae | *Sinojackia xylocarpa* | BOP010105 | KP088852 | | KP088151 |

| | | | | | | |
|---|---|---|---|---|---|---|
| 447 | Styracaceae | *Styrax* sp. | BOP010242 | KP088866 | | KP088261 |
| 448 | Tamaricaceae | *Tamarix chinensis* | BOP010456 | KP088874 | KP089331 | KP088392 |
| 449 | Taxaceae | *Amentotaxus sp.* | BOP010495 | KP088471 | KP088934 | |
| 450 | Taxaceae | *Taxus baccata* | BOP010402 | KP088875 | KP089332 | KP089421 |
| 451 | Taxaceae | *Taxus cuspidata* | BOP010399 | KP088876 | KP089333 | KP089420 |
| 452 | Taxaceae | *Taxus cuspidata* | BOP010401 | KP088877 | KP089334 | KP089422 |
| 453 | Taxodiaceae | *Metasequoia glyptostroboides* | BOP010414 | KP088696 | KP089163 | KP089423 |
| 454 | Thymelaeaceae | *Edgeworthia chrysantha* | BOP010487 | KP088576 | KP089051 | |
| 455 | Tiliaceae | *Tilia amurensis* | BOP010215 | KP088881 | KP089338 | KP088241 |
| 456 | Tiliaceae | *Tilia amurensis* | BOP010216 | KP088882 | KP089339 | KP088242 |
| 457 | Tiliaceae | *Tilia amurensis* | BOP010217 | KP088883 | KP089340 | KP088243 |
| 458 | Tiliaceae | *Tilia cordata* | BOP010213 | KP088885 | KP089342 | KP088239 |
| 459 | Tiliaceae | *Tilia cordata* | BOP010096 | KP088884 | KP089341 | KP088144 |
| 460 | Tiliaceae | *Tilia tuan* | BOP010095 | KP088886 | KP089343 | KP088143 |
| 461 | Tiliaceae | *Tilia tuan* | BOP010208 | KP088887 | KP089344 | KP088235 |
| 462 | Tiliaceae | *Tilla phatyphyllos* var. *cintifolia* | BOP010205 | KP088888 | KP089345 | KP088233 |
| 463 | Tiliaceae | *Tilla phatyphyllos* var. *matabilis* | BOP010206 | KP088889 | KP089346 | KP088234 |
| 464 | Ulmaceae | *Celtis bungeana* | BOP010058 | KP088521 | KP089003 | KP088122 |
| 465 | Ulmaceae | *Celtis bungeana* | BOP010162 | KP088522 | KP089004 | KP088194 |
| 466 | Ulmaceae | *Celtis koraiensis* | BOP010303 | KP088523 | KP089005 | KP088306 |
| 467 | Ulmaceae | *Celtis biondii* var. *heterophylla* | BOP010055 | KP088520 | KP089002 | KP088119 |
| 468 | Ulmaceae | *Hemiptelea davidii* | BOP010300 | KP088629 | KP089103 | KP088303 |
| 469 | Ulmaceae | *Ulmus castaneifolia* | BOP010307 | KP088893 | KP089350 | KP088310 |
| 470 | Ulmaceae | *Ulmus laciniata* | BOP010304 | KP088894 | | KP088307 |
| 471 | Ulmaceae | *Ulmus laevis* | BOP010015 | KP088895 | KP089351 | KP088085 |
| 472 | Ulmaceae | *Ulmus lamellosa* | BOP010305 | KP088896 | KP089352 | KP088308 |
| 473 | Ulmaceae | *Ulmus macrocarpa* | BOP010013 | KP088897 | KP089353 | KP088083 |
| 474 | Ulmaceae | *Ulmus parvifolia* | BOP010057 | KP088898 | | KP088121 |

| | | | | | | |
|---|---|---|---|---|---|---|
| 475 | Ulmaceae | *Ulmus parvifolia* | BOP010306 | KP088899 | KP089354 | KP088309 |
| 476 | Ulmaceae | *Ulmus parvifolia* | BOP010435 | KP088900 | | KP088378 |
| 477 | Ulmaceae | *Ulmus pumila* | BOP010308 | KP088902 | KP089356 | KP088311 |
| 478 | Ulmaceae | *Ulmus pumila* | BOP010110 | KP088901 | KP089355 | KP088155 |
| 479 | Ulmaceae | *Zelkova schneideriana* | BOP010301 | KP088926 | | KP088304 |
| 480 | Ulmaceae | *Zelkova serrata* | BOP010302 | KP088927 | KP089378 | KP088305 |
| 481 | Ulmaceae | *Zelkova sinica* | BOP010447 | KP088928 | | KP088387 |
| 482 | Verbenaceae | *Callicarpa bodinieri* | BOP010134 | KP088502 | KP088987 | |
| 483 | Verbenaceae | *Caryopteris clandonensis* | BOP010086 | KP088512 | KP088995 | KP088135 |
| 484 | Verbenaceae | *Clerodendrum trichotomum* | BOP010467 | KP088539 | KP089017 | KP088400 |
| 485 | Verbenaceae | *Vitex negundo* var. *negundo* | BOP010276 | KP088916 | | KP088291 |
| 486 | Vitaceae | *Ampelopsis aconitifolia* | BOP010145 | KP088473 | KP088956 | KP088181 |
| 487 | Vitaceae | *Parthenocissus thomsoni* | BOP010471 | KP088703 | KP089167 | KP088401 |
| 488 | Vitaceae | *Parthenocissus tricuspidata* | BOP010173 | KP088704 | | |
| 489 | Vitaceae | *Vitis* sp. | BOP010263 | KP088917 | KP089370 | KP088280 |
| 490 | Vitaceae | *Vitis vinifera* | BOP010143 | KP088918 | KP089371 | KP088179 |

**Table S5. Samples of seven groups representing seed plants for candidate barcode resolution testing. The sequences of taxa without vouchers were downloaded from GenBank.**

| Family | Species | Collection locality | Voucher | *rbcL* b | *matK* | *trnH-psbA* | *ycf1* b |
|---|---|---|---|---|---|---|---|
| Araliaceae | *Panax bipinnatifidus* | China, Yunnan, Gongshan | BOP004810 | KM210116 | KM210138 | KM210182 | KP089439 |
| Araliaceae | *Panax bipinnatifidus* | China, Hubei, Shennongjia | BOP004823 | KM210117 | KM210139 | KM210183 | KP089440 |
| Araliaceae | *Panax bipinnatifidus* | China, Sichuan, Muli | BOP004863 | KM210118 | KM210140 | KM210184 | KP089441 |
| Araliaceae | *Panax bipinnatifidus* | China, Yunnan, Deqin | BOP004883 | KM210119 | KM210141 | KM210185 | KP089442 |
| Araliaceae | *Panax bipinnatifidus* | Thailand, Chiang Mam | BOP004949 | KM210120 | KM210142 | KM210186 | KP089445 |
| Araliaceae | *Panax ginseng* | China, Jilin, Tongliao | BOP004420 | KP089454 | KP089459 | KP089428 | KP089435 |
| Araliaceae | *Panax ginseng* | | | AY582139 | AY582139 | AY582139 | AY582139 |
| Araliaceae | *Panax japonica* | Japan, Shizuoka | BOP004280 | KP089453 | KP089458 | KP089427 | KP089432 |
| Araliaceae | *Panax japonica* | Japan, Toyama | BOP004320 | KM210125 | KM210147 | KM210191 | KP089433 |
| Araliaceae | *Panax japonica* | Japan, Gifu | BOP004330 | KM210126 | KM210148 | KM210192 | KP089434 |
| Araliaceae | *Panax notoginseng* | China, Yunnan, Wenshan | A8 | KJ566590 | KJ566590 | KJ566590 | KJ566590 |
| Araliaceae | *Panax notoginseng* | China, Yunnan, Qiubei | BOP004001 | KP089451 | KP089456 | KP089425 | KP089430 |
| Araliaceae | *Panax notoginseng* | China, Yunnan, Yanshan | BOP004151 | KP089452 | KP089457 | KP089426 | KP089431 |
| Araliaceae | *Panax notoginseng* | China, Yunnan, Wenshan | BOP004470 | KM210127 | KM210149 | KM210193 | KP089437 |
| Araliaceae | *Panax notoginseng* | China, Yunnan, Wenshan | BOP004477 | KM210129 | KM210151 | KM210195 | KP089438 |
| Araliaceae | *Panax pseudoginseng* | Nepal, Gotehola | BOP004966 | KM210130 | KM210152 | KM210196 | KP089447 |
| Araliaceae | *Panax pseudoginseng* | Nepal, Gotehola | BOP004968 | KM210131 | KM210153 | KM210197 | KP089448 |
| Araliaceae | *Panax quinquefolius* | China, Jilin, Fuyuan | BOP005001 | KM210132 | KM210154 | KM210198 | KP089449 |
| Araliaceae | *Panax quinquefolius* | USA, Virginia, Giles | BOP005009 | KM210133 | KM210155 | KM210199 | KP089450 |
| Araliaceae | *Panax stipuleanatus* | China, Yunnan, Maguan | BOP004425 | KM210134 | KM210156 | KM210200 | KP089436 |
| Araliaceae | *Panax stipuleanatus* | Vietnam, Quang Nam | BOP004964 | KP089455 | KP089460 | KP089429 | KP089446 |
| Araliaceae | *Panax trifolius* | USA, Maryland, Baltimore | BOP004934 | KM210136 | KM210158 | KM210202 | KP089443 |
| Araliaceae | *Panax trifolius* | USA, Maryland, Baltimore | BOP004935 | KM210137 | KM210159 | KM210203 | KP089444 |
| Calycantha | *Calycanthus chinensis* | Linan, Zhejiang, China | 172 | KP089692 | KP089719 | KP089641 | KP089665 |
| Calycantha | *Calycanthus floridus* | North Carolina, USA | 196 | KP089696 | KP089723 | KP089645 | KP089669 |
| Calycantha | *Calycanthus floridus* | North Carolina, USA | 190 | KP089693 | KP089720 | KP089642 | KP089666 |
| Calycantha | *Calycanthus floridus* | Florida,USA | 192 | KP089694 | KP089721 | KP089643 | KP089667 |

| | | | | | | | |
|---|---|---|---|---|---|---|---|
| Calycantha | *Calycanthus floridus* | North Carolina, USA | 194 | KP089695 | KP089722 | KP089644 | KP089668 |
| Calycantha | *Calycanthus occidentalis* | California, USA | 204 | KP089697 | KP089724 | KP089646 | KP089670 |
| Calycantha | *Chimonanthus campanulatus* | Kunming Botanical Garden, CAS,Yunnan,China | 214 | KP089698 | KP089725 | KP089647 | KP089671 |
| Calycantha | *Chimonanthus gramatus* | Linan, Zhejiang, China | 216.2 | KP089700 | KP089727 | | KP089673 |
| Calycantha | *Chimonanthus gramatus* | Lushan Botanical Garden, Jiujing, Jiangxi, China | 217 | KP089701 | KP089728 | KP089648 | KP089674 |
| Calycantha | *Chimonanthus gramatus* | Linan, Zhejiang, China | 216.1 | KP089699 | KP089726 | | KP089672 |
| Calycantha | *Chimonanthus nitens* | Chaozhou, Fujian, China | 218 | KP089702 | KP089729 | KP089649 | KP089675 |
| Calycantha | *Chimonanthus nitens* | Guilin, Guangxi, China | 224 | KP089703 | KP089730 | KP089650 | KP089676 |
| Calycantha | *Chimonanthus nitens* | Guiyang, Guizhou, China | 266 | KP089704 | KP089731 | KP089651 | KP089677 |
| Calycantha | *Chimonanthus nitens* | Mt. Wuyishan, Fujian, China | 277 | KP089705 | KP089732 | KP089652 | KP089678 |
| Calycantha | *Chimonanthus praecox* | Beijing Botanical Garden, CAS, Beijing, China | 287 | KP089706 | KP089733 | KP089653 | KP089679 |
| Calycantha | *Chimonanthus praecox* | Beijing Botanical Garden, CAS, Beijing, China | 288 | KP089707 | KP089734 | KP089654 | KP089680 |
| Calycantha | *Chimonanthus praecox* | Beijing Botanical Garden, CAS, Beijing, China | 289 | KP089708 | KP089735 | KP089655 | KP089681 |
| Calycantha | *Chimonanthus praecox* | Beijing Botanical Garden, CAS, Beijing, China | 290 | KP089709 | KP089736 | KP089656 | KP089682 |
| Calycantha | *Chimonanthus salicifolius* | Hanzhou Botanical Garden, Hangzhou, Zhejiang, China | 299 | KP089710 | KP089737 | KP089657 | KP089683 |
| Calycantha | *Chimonanthus salicifolius* | Hanzhou Botanical Garden, Hangzhou, Zhejiang, China | 304 | KP089711 | KP089738 | KP089658 | KP089684 |
| Calycantha | *Chimonanthus salicifolius* | Kunming Botanical Garden, CAS,Yunnan,China | 306 | KP089712 | KP089739 | KP089659 | KP089685 |
| Calycantha | *Chimonanthus salicifolius* | Kunming Botanical Garden, CAS,Yunnan,China | 307 | KP089713 | KP089740 | KP089660 | KP089686 |
| Calycantha | *Chimonanthus salicifolius* | Kunming Botanical Garden, CAS,Yunnan,China | 308 | KP089714 | KP089741 | KP089661 | KP089687 |
| Calycantha | *Chimonanthus salicifolius* | Kunming Botanical Garden, CAS,Yunnan,China | 309 | KP089715 | KP089742 | KP089662 | KP089688 |
| Calycantha | *Chimonanthus zhejiangensis* | Hanzhou Botanical Garden, Hangzhou, Zhejiang, China | 319 | KP089716 | KP089743 | | KP089689 |
| Calycantha | *Chimonanthus zhejiangensis* | Hanzhou Botanical Garden, Hangzhou, Zhejiang, China | 324 | KP089717 | KP089744 | KP089663 | KP089690 |
| Calycantha | *Idiospermum australiense* | New York Botanical Garden, USA | 325 | KP089718 | KP089745 | KP089664 | KP089691 |
| Fagaceae | *Quercus aliena* | Yixian, Hebei, China | BOP009901 | KP089911 | KP089926 | KP089886 | KP089896 |
| Fagaceae | *Quercus aliena* | Beijing Botanical Garden, CAS, Beijing, China | BOP010439 | KP088805 | KP089264 | | KP088382 |
| Fagaceae | *Quercus aliena* | Mt.Jigongshan, Xinyang, Henan, China | BOP017224 | KP089916 | KP089931 | | KP089901 |
| Fagaceae | *Quercus aliena* | Mt.Jigongshan, Xinyang, Henan, China | BOP017484 | KP089914 | KP089929 | | KP089899 |
| Fagaceae | *Quercus aliena* | Beijing Botanical Garden, CAS, Beijing, China | BOP010423 | KP088804 | KP089263 | | KP088366 |
| Fagaceae | *Quercus aliena* var.*acuteserrata* | Beijing Botanical Garden, CAS, Beijing, China | BOP010421 | KP088801 | KP089260 | | KP088364 |

| | | | | | | | |
|---|---|---|---|---|---|---|---|
| Fagaceae | *Quercus aliena* var.*acuteserrata* | Beijing Botanical Garden, CAS, Beijing, China | BOP010422 | KP088802 | KP089261 | | KP088365 |
| Fagaceae | *Quercus aliena* var.*acuteserrata* | Beijing Botanical Garden, CAS, Beijing, China | BOP010442 | KP088803 | KP089262 | | KP088385 |
| Fagaceae | *Quercus baronii* | Beijing Botanical Garden, CAS, Beijing, China | BOP010441 | KP088806 | KP089265 | | KP088384 |
| Fagaceae | *Quercus dentata* | Beijing Botanical Garden, CAS, Beijing, China | BOP010271 | KP088807 | KP089266 | | KP088286 |
| Fagaceae | *Quercus dentata* | Mt.Jigongshan, Xinyang, Henan, China | BOP017485 | KP089915 | KP089930 | | KP089900 |
| Fagaceae | *Quercus fabri* | Beijing Botanical Garden, CAS, Beijing, China | BOP010420 | KP088808 | KP089267 | | KP088363 |
| Fagaceae | *Quercus fabri* | Beijing Botanical Garden, CAS, Beijing, China | BOP010426 | KP088809 | KP089268 | | KP088369 |
| Fagaceae | *Quercus gambelii* | Beijing Botanical Garden, CAS, Beijing, China | BOP010431 | KP088810 | KP089269 | | KP088374 |
| Fagaceae | *Quercus glandulifera* var.*brevipetiolata* | Beijing Botanical Garden, CAS, Beijing, China | BOP010427 | KP088811 | KP089270 | | KP088370 |
| Fagaceae | *Quercus macrocarpa* | Beijing Botanical Garden, CAS, Beijing, China | BOP010428 | KP088812 | KP089272 | | KP088371 |
| Fagaceae | *Quercus macrocarpa* | Beijing Botanical Garden, CAS, Beijing, China | BOP010443 | | KP089271 | | |
| Fagaceae | *Quercus mongolica* | Hulin, Heilongjiang, China | BOP009349 | KP089902 | KP089917 | KP089877 | KP089887 |
| Fagaceae | *Quercus mongolica* | Shangzhi, Heilongjiang, China | BOP009373 | KP089903 | KP089918 | KP089878 | KP089888 |
| Fagaceae | *Quercus mongolica* | Huadian, Jilin, China | BOP009445 | KP089904 | KP089919 | KP089879 | KP089889 |
| Fagaceae | *Quercus mongolica* | Fengcheng, Liaoning, China | BOP009493 | KP089905 | KP089920 | KP089880 | KP089890 |
| Fagaceae | *Quercus mongolica* | Mt.Jigongshan, Xinyang, Henan, China | BOP017344 | KP089912 | KP089927 | | KP089897 |
| Fagaceae | *Quercus palustris* | Beijing Botanical Garden, CAS, Beijing, China | BOP010429 | KP088813 | KP089273 | | KP088372 |
| Fagaceae | *Quercus robur* | Beijing Botanical Garden, CAS, Beijing, China | BOP010418 | KP088814 | KP089274 | | KP088361 |
| Fagaceae | *Quercus robur* | Beijing Botanical Garden, CAS, Beijing, China | BOP010419 | KP088815 | KP089275 | | KP088362 |
| Fagaceae | *Quercus rubra* | Beijing Botanical Garden, CAS, Beijing, China | BOP010433 | KP088816 | KP089276 | | KP088376 |
| Fagaceae | *Quercus serrata* | Beijing Botanical Garden, CAS, Beijing, China | BOP010440 | KP088817 | KP089277 | | KP088383 |
| Fagaceae | *Quercus sp1* | Beijing Botanical Garden, CAS, Beijing, China | BOP010417 | KP088818 | KP089278 | | KP088360 |
| Fagaceae | *Quercus sp2* | Beijing Botanical Garden, CAS, Beijing, China | BOP010425 | KP088819 | KP089279 | | KP088368 |
| Fagaceae | *Quercus sp3* | Beijing Botanical Garden, CAS, Beijing, China | BOP010430 | KP088820 | KP089280 | | KP088373 |
| Fagaceae | *Quercus sp4* | Mt.Jigongshan, Xinyang, Henan, China | BOP017467 | KP089913 | KP089928 | | KP089898 |
| Fagaceae | *Quercus stellata* | Beijing Botanical Garden, CAS, Beijing, China | BOP010424 | KP088821 | KP089281 | | KP088367 |
| Fagaceae | *Quercus variabilis* | Beijing Botanical Garden, CAS, Beijing, China | BOP010445 | KP088822 | KP089282 | | KP088386 |
| Fagaceae | *Quercus wutaishanica* | Shenyang, Liaoning, China | BOP009541 | KP089906 | KP089921 | KP089881 | KP089891 |

| | | | | | | | |
|---|---|---|---|---|---|---|---|
| Fagaceae | *Quercus wutaishanica* | Meixian, Shaanxi, China | BOP009733 | KP089907 | KP089922 | KP089882 | KP089892 |
| Fagaceae | *Quercus wutaishanica* | Yanan, Shaanxi, China | BOP009781 | KP089908 | KP089923 | KP089883 | KP089893 |
| Fagaceae | *Quercus wutaishanica* | Yuzhong, Gansu, China | BOP009829 | KP089909 | KP089924 | KP089884 | KP089894 |
| Fagaceae | *Quercus wutaishanica* | Qingdao, Shandong, China | BOP009877 | KP089910 | KP089925 | KP089885 | KP089895 |
| Iridaceae | *Iris anguifuga* | Harbin, Heilongjiang, China | z020 | KP089552 | KP089597 | KP089462 | KP089507 |
| Iridaceae | *Iris bloudowii* | Hami, Xinjiang, China | z092 | KP089553 | KP089598 | KP089463 | KP089508 |
| Iridaceae | *Iris bulleyana* | Chayu, Tibet, China | z222 | KP089555 | KP089600 | KP089465 | KP089510 |
| Iridaceae | *Iris bulleyana* var.*alba* | Huanglong, Yunnan, China | z213 | KP089554 | KP089599 | KP089464 | KP089509 |
| Iridaceae | *Iris chrysograghes* | Muli, Sichuan, China | z067 | KP089556 | KP089601 | KP089466 | KP089511 |
| Iridaceae | *Iris chrysographes* | Linzhi, Tibet, China | z224 | KP089557 | KP089602 | KP089467 | KP089512 |
| Iridaceae | *Iris collettii* | Muli, Sichuan, China | z068 | KP089558 | KP089603 | KP089468 | KP089513 |
| Iridaceae | *Iris collettii* | Dali, Yunnan, China | z094 | KP089559 | KP089604 | KP089469 | KP089514 |
| Iridaceae | *Iris darvarica* | Beijing Botanical Garden, CAS, Beijing, China | z022 | KP089560 | KP089605 | KP089470 | KP089515 |
| Iridaceae | *Iris decora* | Jiulong, Sichuan, China | z062 | KP089561 | KP089606 | KP089471 | KP089516 |
| Iridaceae | *Iris delavayi* | Dali, Yunnan, China | z095 | KP089562 | KP089607 | KP089472 | KP089517 |
| Iridaceae | *Iris delavayi* | Huanglong, Yunnan, China | z211 | KP089563 | KP089608 | KP089473 | KP089518 |
| Iridaceae | *Iris dichotoma* | Changli, Hebei, China | z108 | KP089564 | KP089609 | KP089474 | KP089519 |
| Iridaceae | *Iris domestica* | Huashan, Shanxi, China | z031 | KP089551 | KP089596 | KP089461 | KP089506 |
| Iridaceae | *Iris ensata* | Benxi, Liaoning, China | z110 | KP089566 | KP089611 | KP089476 | KP089521 |
| Iridaceae | *Iris ensata* | Daxinganling, Helongjiang, China | z003 | KP089565 | KP089610 | KP089475 | KP089520 |
| Iridaceae | *Iris forrestii* | Lijiang, Yunnan, China | z096 | KP089567 | KP089612 | KP089477 | KP089522 |
| Iridaceae | *Iris forrestii* | Huanglong, Yunnan, China | z217 | KP089568 | KP089613 | KP089478 | KP089523 |
| Iridaceae | *Iris germanica* | New Delhi, India | z085 | KP089569 | KP089614 | KP089479 | KP089524 |
| Iridaceae | *Iris lactea* var.*chinensis* | Zuoni, Gansu, China | z040 | KP089570 | KP089615 | KP089480 | KP089525 |
| Iridaceae | *Iris laevigata* | Hailin, Heilongjiang, China | z008 | KP089571 | KP089616 | KP089481 | KP089526 |
| Iridaceae | *Iris loczyi* | Huzu, Qinghai, China | z047 | KP089572 | KP089617 | KP089482 | KP089527 |
| Iridaceae | *Iris mandshurica* | Harbin, Heilongjiang, China | z009 | KP089573 | KP089618 | KP089483 | KP089528 |
| Iridaceae | *Iris pandurata* | Lanzhou, Gansu, China | z044 | KP089574 | KP089619 | KP089484 | KP089529 |
| Iridaceae | *Iris pseudacorus* | Shenyang, Liaoning, China | z016 | KP089576 | KP089621 | KP089486 | KP089531 |
| Iridaceae | *Iris pseudacorus* | Muli, Sichuan, China | z069 | KP089577 | KP089622 | KP089487 | KP089532 |

| | | | | | | | |
|---|---|---|---|---|---|---|---|
| Iridaceae | *Iris rossii* | Dandong, Liaoning, China | z111 | KP089578 | KP089623 | KP089488 | KP089533 |
| Iridaceae | *Iris ruthenica* var.*nana* | Minxian, Gansu, China | z041 | KP089579 | KP089624 | KP089489 | KP089534 |
| Iridaceae | *Iris sanguinea* | Yichun, Heilongjiang, China | z002 | KP089580 | KP089625 | KP089490 | KP089535 |
| Iridaceae | *Iris sanguinea* | Benxi, Liaoning, China | z109 | KP089581 | KP089626 | KP089491 | KP089536 |
| Iridaceae | *Iris scariosa* | Gongliu, Xinjiang, China | z090 | KP089582 | KP089627 | KP089492 | KP089537 |
| Iridaceae | *Iris setosa* | Harbin, Heilongjiang, China | z215 | KP089583 | KP089628 | KP089493 | KP089538 |
| Iridaceae | *Iris sibirica* | Beijing Botanical Garden, CAS, Beijing, China | z021 | KP089584 | KP089629 | KP089494 | KP089539 |
| Iridaceae | *Iris sp* | Chongqing, China | z231 | KP089587 | KP089632 | KP089497 | KP089542 |
| Iridaceae | *Iris speculatrix* | Xingan, Guangxi, China | z081 | KP089585 | KP089630 | KP089495 | KP089540 |
| Iridaceae | *Iris speculatrix* | Huanglong, Yunnan, China | z219 | KP089586 | KP089631 | KP089496 | KP089541 |
| Iridaceae | *Iris subdichotoma* | Zhongdian, Yunnan, China | z102 | KP089588 | KP089633 | KP089498 | KP089543 |
| Iridaceae | *Iris tectorum* | Shennongjia, Hubei, China | z018 | KP089589 | KP089634 | KP089499 | KP089544 |
| Iridaceae | *Iris tigridia* | Xining, Qinghai, China | z048 | KP089575 | KP089620 | KP089485 | KP089530 |
| Iridaceae | *Iris tigridia* | Bashang, Nei Monggol, China | z106 | KP089590 | KP089635 | KP089500 | KP089545 |
| Iridaceae | *Iris tigridia* | Harbin, Heilongjiang, China | z216 | KP089591 | KP089636 | KP089501 | KP089546 |
| Iridaceae | *Iris typhifolia* | Anda, Heilongjiang, China | z013 | KP089592 | KP089637 | KP089502 | KP089547 |
| Iridaceae | *Iris uniflora* | Yichun, Heilongjiang, China | z012 | KP089593 | KP089638 | KP089503 | KP089548 |
| Iridaceae | *Iris uniflora* | Harbin, Heilongjiang, China | z207 | KP089594 | KP089639 | KP089504 | KP089549 |
| Iridaceae | *Moraea iridioides* | Harbin, Heilongjiang, China | z027 | KP089595 | KP089640 | KP089505 | KP089550 |
| Paeoniacea | *Paeonia cathayana* | Songxian, Henan, China | BOP003923 | | KJ946110 | KJ946180 | KP089767 |
| Paeoniacea | *Paeonia decomposita* | Maerkang, Sichuan, China | BOP001444 | KP089758 | KP089749 | | KP089768 |
| Paeoniacea | *Paeonia decomposita* | Maerkang, Sichuan, China | BOP001538 | KJ946161 | KJ946113 | KJ946182 | KP089769 |
| Paeoniacea | *Paeonia decomposita* | Kangding, Sichuan, China | BOP003948 | KP089764 | KP089755 | | KP089770 |
| Paeoniacea | *Paeonia delavayi* | Ninglang, Yunnan, China | BOP002046 | KP089761 | KP089752 | | KP089771 |
| Paeoniacea | *Paeonia delavayi* | Bomi, Tibet, China | BOP002056 | KJ946163 | KJ946115 | KJ946184 | KP089772 |
| Paeoniacea | *Paeonia delavayi* | Kunming, Yunnan, China | BOP002139 | KJ946165 | KJ946117 | KJ946186 | KP089773 |
| Paeoniacea | *Paeonia delavayi* | Muli, Sichuan, China | BOP002185 | KJ946167 | KJ946119 | KJ946187 | KP089774 |
| Paeoniacea | *Paeonia delavayi* | Zhongdian, Yunnan, China | BOP002190 | KJ946164 | KJ946116 | KJ946185 | KP089775 |
| Paeoniacea | *Paeonia delavayi* | Zhongdian, Yunnan, China | BOP002201 | KP089763 | KP089753 | | KP089776 |
| Paeoniacea | *Paeonia jishanensis* | Huayin, Shaanxi, China | BOP001585 | KJ946168 | KJ946120 | KJ946188 | KP089777 |

| | | | | | | | |
|---|---|---|---|---|---|---|---|
| Paeoniacea | *Paeonia jishanensis* | Jisan, Shanxi, China | BOP001643 | KJ946169 | KJ946121 | KJ946189 | KP089778 |
| Paeoniacea | *Paeonia jishanensis* | Yongji, Shanxi, China | BOP001735 | KP089759 | KP089750 | | KP089779 |
| Paeoniacea | *Paeonia ludlowii* | Milin, Tibet, China | BOP001370 | KJ946170 | KJ946122 | KJ946190 | KP089780 |
| Paeoniacea | *Paeonia ludlowii* | Milin, Tibet, China | BOP002074 | KP089762 | KP089754 | | KP089781 |
| Paeoniacea | *Paeonia ostii* | Bozhou, Anhui, China | BOP001481 | KJ946171 | KJ946124 | KJ946192 | KP089782 |
| Paeoniacea | *Paeonia ostii* | Shennongjia, Hubei, China | BOP003912 | KJ946173 | KJ946126 | KJ946194 | |
| Paeoniacea | *Paeonia qiui* | Shennongjia, Hubei, China | BOP001429 | KP089757 | KP089748 | KP089746 | KP089784 |
| Paeoniacea | *Paeonia qiui* | Baokang, Hubei, China | BOP001981 | KJ946174 | KJ946127 | KJ946195 | KP089785 |
| Paeoniacea | *Paeonia qiui* | Baokang, Hubei, China | BOP001985 | | KP089751 | | KP089786 |
| Paeoniacea | *Paeonia rockii* | Tianshui, Gansu, China | BOP001888 | KJ946179 | KJ946132 | KJ946200 | KP089787 |
| Paeoniacea | *Paeonia rockii* | Luoyang, Shaanxi, China | BOP001903 | KJ946175 | KJ946128 | KJ946196 | KP089788 |
| Paeoniacea | *Paeonia rockii* | Taibai, Shaanxi, China | BOP001923 | KJ946177 | KJ946130 | KJ946198 | KP089789 |
| Paeoniacea | *Paeonia rockii* | Tongchuan, Shaanxi, China | BOP001962 | KJ946178 | KJ946131 | KJ946199 | KP089790 |
| Paeoniacea | *Paeonia rockii* | Neixiang, Henan, China | BOP003615 | KJ946176 | KJ946129 | KJ946197 | KP089791 |
| Paeoniacea | *Paeonia rockii* | Ganquan, Shaanxi, China | BOP003950 | KP089765 | KP089756 | KP089747 | KP089792 |
| Pinaceae | *Pinus albicaulis* | | | FJ899566 | FJ899566 | FJ899566 | FJ899566 |
| Pinaceae | *Pinus amamiana* | | | JN854226 | JN854226 | JN854226 | JN854226 |
| Pinaceae | *Pinus aristata* | | | FJ899567 | FJ899567 | FJ899567 | FJ899567 |
| Pinaceae | *Pinus arizonica* | | | JN854225 | JN854225 | JN854225 | JN854225 |
| Pinaceae | *Pinus arizonica* var.*cooperi* | | | JN854216 | JN854216 | JN854216 | JN854216 |
| Pinaceae | *Pinus armandii* | Beijing Botanical Garden, CAS, Beijing, China | BOP010394 | KP088729 | KP089189 | | KP089404 |
| Pinaceae | *Pinus armandii* | | | FJ899568 | FJ899568 | FJ899568 | FJ899568 |
| Pinaceae | *Pinus attenuata* | | | FJ899569 | FJ899569 | FJ899569 | FJ899569 |
| Pinaceae | *Pinus ayacahuite* | | | FJ899570 | FJ899570 | FJ899570 | FJ899570 |
| Pinaceae | *Pinus banksiana* | Beijing Botanical Garden, CAS, Beijing, China | BOP010398 | KP088730 | KP089190 | | KP089408 |
| Pinaceae | *Pinus banksiana* | | | FJ899571 | | FJ899571 | FJ899571 |
| Pinaceae | *Pinus brutia* | Adelaide Botanic Garden,Adelaide, Australia | BOP017802 | KP089942 | KP089952 | | KP089932 |
| Pinaceae | *Pinus brutia* | | | JN854224 | JN854224 | JN854224 | JN854224 |
| Pinaceae | *Pinus bungeana* | Beijing Botanical Garden, CAS, Beijing, China | BOP010376 | KP088731 | KP089191 | | KP089391 |
| Pinaceae | *Pinus bungeana* | | | JN854223 | JN854223 | JN854223 | JN854223 |

| | | | | | | | |
|---|---|---|---|---|---|---|---|
| Pinaceae | *Pinus canariensis* | Adelaide Botanic Garden,Adelaide, Australia | BOP017803 | KP089943 | KP089953 | | KP089933 |
| Pinaceae | *Pinus canariensis* | | | FJ899572 | | FJ899572 | FJ899572 |
| Pinaceae | *Pinus caribaea* | | | JN854222 | JN854222 | JN854222 | JN854222 |
| Pinaceae | *Pinus cembroides* | | | JN854220 | JN854220 | JN854220 | JN854220 |
| Pinaceae | *Pinus chiapensis* | | | JN854219 | JN854219 | JN854219 | JN854219 |
| Pinaceae | *Pinus clausa* | | | JN854217 | JN854217 | JN854217 | JN854217 |
| Pinaceae | *Pinus contorta* | | | EU998740 | | EU998740 | EU998740 |
| Pinaceae | *Pinus contorta* | | | NC011153 | | NC011153 | NC011153 |
| Pinaceae | *Pinus coulteri* | | | JN854215 | JN854215 | JN854215 | JN854215 |
| Pinaceae | *Pinus cubensis* | | | JN854214 | JN854214 | JN854214 | JN854214 |
| Pinaceae | *Pinus culminicola* | | | JN854213 | JN854213 | JN854213 | JN854213 |
| Pinaceae | *Pinus dalatensis* | | | JN854211 | JN854211 | JN854211 | JN854211 |
| Pinaceae | *Pinus densata* | | | JN854209 | JN854209 | JN854209 | JN854209 |
| Pinaceae | *Pinus densiflora* | Beijing Botanical Garden, CAS, Beijing, China | BOP010367 | KP088732 | KP089192 | | KP089385 |
| Pinaceae | *Pinus densiflora* | | | JN854210 | JN854210 | JN854210 | JN854210 |
| Pinaceae | *Pinus devoniana* | | | JN854208 | JN854208 | JN854208 | JN854208 |
| Pinaceae | *Pinus discolor* | | | JN854207 | JN854207 | JN854207 | JN854207 |
| Pinaceae | *Pinus douglasiana* | | | JN854205 | JN854205 | JN854205 | JN854205 |
| Pinaceae | *Pinus echinata* | | | JN854204 | JN854204 | JN854204 | JN854204 |
| Pinaceae | *Pinus edulis* | | | JN854203 | JN854203 | JN854203 | JN854203 |
| Pinaceae | *Pinus elliottii* | | | JN854202 | JN854202 | JN854202 | JN854202 |
| Pinaceae | *Pinus engelmannii* | | | JN854201 | JN854201 | JN854201 | JN854201 |
| Pinaceae | *Pinus fenzeliana* var.*dabeshanensis* | | | JN854212 | JN854212 | JN854212 | JN854212 |
| Pinaceae | *Pinus flexilis* | | | FJ899576 | | FJ899576 | FJ899576 |
| Pinaceae | *Pinus fragilissima* | | | JN854200 | JN854200 | JN854200 | JN854200 |
| Pinaceae | *Pinus gerardiana* | | | EU998741 | EU998741 | EU998741 | EU998741 |
| Pinaceae | *Pinus gerardiana* | | | NC011154 | NC011154 | NC011154 | - |
| Pinaceae | *Pinus glabra* | | | JN854199 | JN854199 | JN854199 | JN854199 |
| Pinaceae | *Pinus greggii* | | | JN854198 | JN854198 | JN854198 | JN854198 |
| Pinaceae | *Pinus griffithii* | Beijing Botanical Garden, CAS, Beijing, China | BOP010379 | KP088733 | KP089193 | | KP089393 |

| | | | | | | | |
|---|---|---|---|---|---|---|---|
| Pinaceae | *Pinus halepensis* | | | JN854197 | JN854197 | JN854197 | JN854197 |
| Pinaceae | *Pinus hartwegii* | | | JN854196 | JN854196 | JN854196 | JN854196 |
| Pinaceae | *Pinus hartwegii* | | | JN854206 | JN854206 | JN854206 | JN854206 |
| Pinaceae | *Pinus heldreichii* | | | JN854195 | JN854195 | JN854195 | JN854195 |
| Pinaceae | *Pinus hwangshanensis* | | | JN854194 | JN854194 | JN854194 | JN854194 |
| Pinaceae | *Pinus jeffreyi* | Melbourne Botanic Garden,Melbourne, Australia | BOP017842 | KP089945 | KP089955 | | KP089935 |
| Pinaceae | *Pinus jeffreyi* | | | JN854193 | JN854193 | JN854193 | JN854193 |
| Pinaceae | *Pinus johannis* | | | JN854192 | JN854192 | JN854192 | JN854192 |
| Pinaceae | *Pinus kesiya* | | | JN854191 | JN854191 | JN854191 | JN854191 |
| Pinaceae | *Pinus koraiensis* | Beijing Botanical Garden, CAS, Beijing, China | BOP010403 | KP088734 | KP089194 | | KP089410 |
| Pinaceae | *Pinus koraiensis* | | | AY228468 | AY228468 | AY228468 | AY228468 |
| Pinaceae | *Pinus koraiensis* | | | NC004677 | NC004677 | NC004677 | NC004677 |
| Pinaceae | *Pinus krempfii* | | | EU998742 | EU998742 | EU998742 | EU998742 |
| Pinaceae | *Pinus krempfii* | | | NC011155 | NC011155 | NC011155 | NC011155 |
| Pinaceae | *Pinus kwangtungensis* | | | JN854153 | JN854153 | JN854153 | JN854153 |
| Pinaceae | *Pinus lambertiana* | | | EU998743 | EU998743 | EU998743 | EU998743 |
| Pinaceae | *Pinus lambertiana* | | | FJ899577 | | FJ899577 | FJ899577 |
| Pinaceae | *Pinus lambertiana* | | | NC011156 | NC011156 | NC011156 | NC011156 |
| Pinaceae | *Pinus latteri* | | | JN854190 | JN854190 | JN854190 | JN854190 |
| Pinaceae | *Pinus lawsonii* | | | JN854188 | JN854188 | JN854188 | JN854188 |
| Pinaceae | *Pinus leiophylla* | | | JN854187 | JN854187 | JN854187 | JN854187 |
| Pinaceae | *Pinus leiophylla* var.*chihuahuana* | | | FJ899575 | FJ899575 | FJ899575 | FJ899575 |
| Pinaceae | *Pinus leiophylla* var.*chihuahuana* | | | JN854218 | JN854218 | JN854218 | JN854218 |
| Pinaceae | *Pinus lumholtzii* | | | JN854186 | JN854186 | JN854186 | JN854186 |
| Pinaceae | *Pinus massoniana* | Mt.Jigongshan, Xinyang, Henan, China | BOP017268 | KP089949 | KP089959 | | KP089939 |
| Pinaceae | *Pinus massoniana* | | | JN854185 | JN854185 | JN854185 | JN854185 |
| Pinaceae | *Pinus maximartinezii* | | | JN854184 | JN854184 | JN854184 | JN854184 |
| Pinaceae | *Pinus merkusii* | | | FJ899579 | FJ899579 | FJ899579 | FJ899579 |
| Pinaceae | *Pinus monophylla* | | | EU998745 | EU998745 | EU998745 | EU998745 |

| | | | | | | | |
|---|---|---|---|---|---|---|---|
| Pinaceae | *Pinus monophylla* | | | NC011158 | NC011158 | NC011158 | NC011158 |
| Pinaceae | *Pinus montezumae* | | | JN854183 | JN854183 | JN854183 | JN854183 |
| Pinaceae | *Pinus morrisonicola* | | | JN854182 | JN854182 | JN854182 | JN854182 |
| Pinaceae | *Pinus mugo* | | | JN854181 | JN854181 | JN854181 | JN854181 |
| Pinaceae | *Pinus muricata* | | | JN854180 | JN854180 | JN854180 | JN854180 |
| Pinaceae | *Pinus nelsonii* | | | EU998746 | EU998746 | EU998746 | EU998746 |
| Pinaceae | *Pinus nelsonii* | | | NC011159 | NC011159 | NC011159 | NC011159 |
| Pinaceae | *Pinus nigra* | Beijing Botanical Garden, CAS, Beijing, China | BOP010406 | KP088735 | KP089195 | | KP089411 |
| Pinaceae | *Pinus nigra* | | | JN854179 | JN854179 | JN854179 | JN854179 |
| Pinaceae | *Pinus occidentalis* | | | JN854177 | JN854177 | JN854177 | JN854177 |
| Pinaceae | *Pinus palustris* | | | JN854176 | JN854176 | JN854176 | JN854176 |
| Pinaceae | *Pinus parviflora* | Beijing Botanical Garden, CAS, Beijing, China | BOP017779 | KP089951 | KP089961 | | KP089941 |
| Pinaceae | *Pinus parviflora* var.*pentaphylla* | | | FJ899581 | FJ899581 | FJ899581 | FJ899581 |
| Pinaceae | *Pinus patula* | Melbourne Botanic Garden,Melbourne, Australia | BOP017852 | KP089946 | KP089956 | | KP089936 |
| Pinaceae | *Pinus patula* | | | JN854175 | JN854175 | JN854175 | JN854175 |
| Pinaceae | *Pinus peuce* | | | FJ899582 | | FJ899582 | FJ899582 |
| Pinaceae | *Pinus pinaster* | | | FJ899583 | FJ899583 | FJ899583 | FJ899583 |
| Pinaceae | *Pinus pinceana* | | | JN854174 | JN854174 | JN854174 | JN854174 |
| Pinaceae | *Pinus pinea* | | | JN854173 | JN854173 | JN854173 | JN854173 |
| Pinaceae | *Pinus ponderosa* | Beijing Botanical Garden, CAS, Beijing, China | BOP010377 | KP088736 | KP089196 | | KP089392 |
| Pinaceae | *Pinus ponderosa* | | | FJ899555 | FJ899555 | FJ899555 | FJ899555 |
| Pinaceae | *Pinus ponderosa* var.*benthamiana* | | | JN854172 | JN854172 | JN854172 | JN854172 |
| Pinaceae | *Pinus ponderosa* var.*scopulorum* | | | JN854171 | JN854171 | JN854171 | JN854171 |
| Pinaceae | *Pinus pringlei* | | | JN854189 | JN854189 | JN854189 | JN854189 |
| Pinaceae | *Pinus pseudostrobus* | | | JN854169 | JN854169 | JN854169 | JN854169 |
| Pinaceae | *Pinus pseudostrobus* var.*apulcensis* | | | JN854178 | JN854178 | JN854178 | JN854178 |
| Pinaceae | *Pinus pumila* | | | JN854168 | JN854168 | JN854168 | - |

| | | | | | | | |
|---|---|---|---|---|---|---|---|
| Pinaceae | *Pinus pungens* | | | JN854167 | JN854167 | JN854167 | JN854167 |
| Pinaceae | *Pinus quadrifolia* | | | JN854166 | JN854166 | JN854166 | JN854166 |
| Pinaceae | *Pinus radiata* | | | JN854165 | JN854165 | JN854165 | JN854165 |
| Pinaceae | *Pinus remota* | | | JN854164 | JN854164 | JN854164 | JN854164 |
| Pinaceae | *Pinus resinosa* | | | FJ899556 | FJ899556 | FJ899556 | FJ899556 |
| Pinaceae | *Pinus rigida* | | | JN854163 | JN854163 | JN854163 | JN854163 |
| Pinaceae | *Pinus roxburghii* | | | JN854162 | JN854162 | JN854162 | JN854162 |
| Pinaceae | *Pinus rzedowskii* | | | FJ899557 | FJ899557 | FJ899557 | FJ899557 |
| Pinaceae | *Pinus sabiniana* | | | JN854161 | JN854161 | JN854161 | JN854161 |
| Pinaceae | *Pinus serotina* | | | JN854160 | JN854160 | JN854160 | JN854160 |
| Pinaceae | *Pinus sibirica* | | | FJ899558 | FJ899558 | FJ899558 | FJ899558 |
| Pinaceae | *Pinus sp1* | Beijing Botanical Garden, CAS, Beijing, China | BOP010396 | KP088737 | KP089197 | | KP089406 |
| Pinaceae | *Pinus sp2* | Beijing Botanical Garden, CAS, Beijing, China | BOP010400 | KP088738 | KP089198 | | KP089409 |
| Pinaceae | *Pinus sp3* | Beijing Botanical Garden, CAS, Beijing, China | BOP010480 | KP088739 | KP089199 | | KP089418 |
| Pinaceae | *Pinus sp4* | Mt.Jigongshan, Xinyang, Henan, China | BOP017543 | KP089950 | KP089960 | | KP089940 |
| Pinaceae | *Pinus sp5* | Melbourne Botanic Garden,Melbourne, Australia | BOP017841 | KP089944 | KP089954 | | KP089934 |
| Pinaceae | *Pinus squamata* | | | FJ899559 | FJ899559 | FJ899559 | FJ899559 |
| Pinaceae | *Pinus strobiformis* | | | JN854159 | JN854159 | JN854159 | JN854159 |
| Pinaceae | *Pinus strobus* | Beijing Botanical Garden, CAS, Beijing, China | BOP010374 | KP088740 | KP089200 | | KP089389 |
| Pinaceae | *Pinus strobus* | | | FJ899560 | FJ899560 | FJ899560 | FJ899560 |
| Pinaceae | *Pinus sylvestris* | Melbourne Botanic Garden,Melbourne, Australia | BOP017855 | KP089947 | KP089957 | | KP089937 |
| Pinaceae | *Pinus sylvestris* | | | JN854158 | JN854158 | JN854158 | JN854158 |
| Pinaceae | *Pinus sylvestris* var.*mongolica* | Beijing Botanical Garden, CAS, Beijing, China | BOP010409 | KP088741 | KP089201 | | KP089414 |
| Pinaceae | *Pinus sylvestris* var.*sylvestriformis* | Beijing Botanical Garden, CAS, Beijing, China | BOP010397 | KP088742 | KP089202 | | KP089407 |
| Pinaceae | *Pinus tabulaeformis* | Beijing Botanical Garden, CAS, Beijing, China | BOP010362 | KP088743 | KP089203 | | KP089380 |
| Pinaceae | *Pinus tabulaeformis* | Beijing Botanical Garden, CAS, Beijing, China | BOP010363 | KP088744 | KP089204 | | KP089381 |
| Pinaceae | *Pinus tabuliformis* var.*mukdensis* | Herbin, Heilongjiang, China | BOP017895 | KP089948 | KP089958 | | KP089938 |
| Pinaceae | *Pinus taeda* | | | FJ899561 | FJ899561 | FJ899561 | FJ899561 |
| Pinaceae | *Pinus taiwanensis* | | | JN854157 | JN854157 | JN854157 | JN854157 |

| | | | | | | | |
|---|---|---|---|---|---|---|---|
| Pinaceae | *Pinus thunbergii* | Beijing Botanical Garden, CAS, Beijing, China | BOP010384 | KP088745 | KP089205 | | KP089395 |
| Pinaceae | *Pinus thunbergii* | | | FJ899562 | FJ899562 | FJ899562 | FJ899562 |
| Pinaceae | *Pinus torreyana* subsp. *insularis* | | | FJ899563 | | FJ899563 | FJ899563 |
| Pinaceae | *Pinus torreyana* subsp.*torreyana* | | | | | FJ899564 | FJ899564 |
| Pinaceae | *Pinus tropicalis* | | | JN854156 | JN854156 | JN854156 | JN854156 |
| Pinaceae | *Pinus virginiana* | | | JN854155 | JN854155 | JN854155 | JN854155 |
| Pinaceae | *Pinus wallichiana* | | | JN854154 | JN854154 | JN854154 | JN854154 |
| Pinaceae | *Pinus yecorensis* | | | JN854152 | JN854152 | JN854152 | JN854152 |
| Pinaceae | *Pinus yunnanensis* | | | JN854151 | JN854151 | JN854151 | JN854151 |
| Rosaceae | *Prunus armeniaca* | Northeast Forestry University , Haerbin, Heilongjiang, C | 090614 | KP089863 | KP089842 | KP089800 | KP089821 |
| Rosaceae | *Prunus armeniaca* | Jixian, Tianjin, China | XY1 | KP089856 | KP089835 | KP089793 | KP089814 |
| Rosaceae | *Prunus armeniaca* | Beizhen, Liaoning, China | XY2 | KP089857 | KP089836 | KP089794 | KP089815 |
| Rosaceae | *Prunus armeniaca* | Shangzhou, Shanxi, China | XY4 | KP089858 | KP089837 | KP089795 | KP089816 |
| Rosaceae | *Prunus armeniaca* | Beijing, China | XY6 | KP089859 | KP089838 | KP089796 | KP089817 |
| Rosaceae | *Prunus armeniaca* | Jilin, China | XY8 | KP089860 | KP089839 | KP089797 | KP089818 |
| Rosaceae | *Prunus armeniaca* | Jilin, China | XY9 | KP089861 | KP089840 | KP089798 | KP089819 |
| Rosaceae | *Prunus armeniaca* | Beijing Botanical Garden, CAS, Beijing, China | Z33 | KP089862 | KP089841 | KP089799 | KP089820 |
| Rosaceae | *Prunus holosericea* | Cayu, Tibet, China | Q17.1 | KP089864 | KP089843 | KP089801 | KP089822 |
| Rosaceae | *Prunus holosericea* | Cayu, Tibet, China | Q17.2 | KP089865 | KP089844 | KP089802 | KP089823 |
| Rosaceae | *Prunus mume* | Hangzhou, Zhejiang, China | 090407 | KP089866 | KP089845 | KP089803 | KP089824 |
| Rosaceae | *Prunus mume* | Mt.Xiangshan, Beijing, China | BJ05 | KP089869 | KP089848 | KP089806 | KP089827 |
| Rosaceae | *Prunus mume* | Wuhan Botanical Garden, CAS, Wuhan, China | WH02 | KP089867 | KP089846 | KP089804 | KP089825 |
| Rosaceae | *Prunus mume* | Wuhan Botanical Garden, CAS, Wuhan, China | WH03 | KP089868 | KP089847 | KP089805 | KP089826 |
| Rosaceae | *Prunus sibirica* | Beijing Botanical Garden, CAS, Beijing, China | BJ06 | KP089874 | KP089853 | KP089811 | KP089832 |
| Rosaceae | *Prunus sibirica* | Xiongyue, Liaoning, China | XY11 | KP089871 | KP089850 | KP089808 | KP089829 |
| Rosaceae | *Prunus sibirica* | Xiongyue, Liaoning, China | XY12 | KP089872 | KP089851 | KP089809 | KP089830 |
| Rosaceae | *Prunus sibirica* | Xiongyue, Liaoning, China | XY14 | KP089873 | KP089852 | KP089810 | KP089831 |
| Rosaceae | *Prunus sibirica* | Xiongyue, Liaoning, China | XY3 | KP089870 | KP089849 | KP089807 | KP089828 |
| Rosaceae | *Prunus zhengheensis* | Zhenghe,Fujian, China | ZH01 | KP089875 | KP089854 | KP089812 | KP089833 |
| Rosaceae | *Prunus zhengheensis* | Zhenghe,Fujian, China | ZH02 | KP089876 | KP089855 | KP089813 | KP089834 |

**Table S6. Nucleotide diversity of *ycf1* b together with three other markers and their species-discriminating powers in *Pinus* (Pinaceae).**

| Locus | Numbers of samples | species | Matrix size(bp) | π | S | Parsimony informative sites | DNA barcoding (%) | | |
|---|---|---|---|---|---|---|---|---|---|
| | | | | | | | Blast | Tree-building | Distance |
| *matK* | 131 | 107 | 795 | 0.0158 | 79 | 61 | 22.90 | 16.82 | 5.61 |
| *rbcL* | 139 | 112 | 785 | 0.0112 | 56 | 39 | 29.50 | 20.54 | 20.54 |
| *trnH-psbA* | 112 | 102 | 793 | 0.0105 | 77 | 43 | 44.64 | 29.41 | 29.41 |
| *ycf1b* | 138 | 112 | 1654 | 0.0886 | 498 | 435 | 64.49 | 56.25 | 51.79 |
| *matK+rbcL* | 139 | 113 | 1580 | 0.0127 | 135 | 100 | 46.76 | 33.63 | 12.39 |
| *matK+trnH-psbA* | 140 | 115 | 1588 | 0.0213 | 156 | 104 | 53.57 | 41.74 | 19.13 |
| *matK+ycf1b* | 140 | 114 | 2449 | 0.0601 | 577 | 496 | 72.14 | 56.14 | 48.25 |
| *rbcL+trnH-psbA* | 140 | 115 | 1578 | 0.0139 | 133 | 82 | 50.00 | 43.48 | 24.35 |
| *rbcL+ycf1b* | 140 | 114 | 2439 | 0.0470 | 554 | 474 | 71.43 | 58.77 | 54.39 |
| *ycf1b+trnH-psbA* | 140 | 115 | 2447 | 0.0879 | 575 | 478 | 81.43 | 60.87 | 50.43 |

π: Nucleotide diversity

S: Number of polymorphic site

**Table S7. Nucleotide diversity of *ycf1* b together with three other markers and their species-discriminating powers in Calycanthaceae.**

| Locus | Numbers of samples | species | Matrix size(bp) | π | S | Parsimony informative sites | DNA barcoding (%) | | |
|---|---|---|---|---|---|---|---|---|---|
| | | | | | | | Blast | Tree-building | Distance |
| *matK* | 27 | 11 | 770 | 0.0069 | 33 | 9 | 81.49 | 63.64 | 54.55 |
| *rbcL* | 27 | 11 | 474 | 0.0179 | 63 | 36 | 44.44 | 36.36 | 36.36 |
| *trnH-psbA* | 24 | 11 | 476 | 0.0064 | 26 | 1 | 33.33 | 9.09 | 18.18 |
| *ycf1b* | 27 | 11 | 868 | 0.0081 | 62 | 10 | 66.67 | 63.64 | 63.64 |
| *matK+rbcL* | 27 | 11 | 1244 | 0.0111 | 96 | 45 | 100.00 | 63.64 | 63.64 |
| *matK+trnH-psbA* | 27 | 11 | 1246 | 0.0069 | 59 | 10 | 100.00 | 81.82 | 63.64 |
| *matK+ycf1b* | 27 | 11 | 1638 | 0.0075 | 95 | 19 | 100.00 | 81.82 | 72.73 |
| *rbcL+trnH-psbA* | 27 | 11 | 950 | 0.0179 | 89 | 37 | 55.56 | 18.18 | 18.18 |
| *rbcL+ycf1b* | 27 | 11 | 1342 | 0.0115 | 125 | 46 | 81.48 | 63.64 | 63.64 |
| *ycf1b+trnH-psbA* | 27 | 11 | 1344 | 0.0081 | 88 | 11 | 74.07 | 18.18 | 45.45 |

Pi: Nucleotide diversity

S: Number of polymorphic site

**Table S8. Nucleotide diversity of *ycf1* b together with three other markers and their species-discriminating powers in *Iris* (Iridaceae).**

| Locus | Numbers of samples | species | Matrix size(bp) | π | S | Parsimony informative sites | DNA barcoding (%) | | |
|---|---|---|---|---|---|---|---|---|---|
| | | | | | | | Blast | Tree-building | Distance |
| *matK* | 44 | 33 | 799 | 0.0199 | 101 | 75 | 62.22 | 54.55 | 54.55 |
| *rbcL* | 44 | 33 | 638 | 0.0109 | 49 | 30 | 57.78 | 54.55 | 51.52 |
| *trnH-psbA* | 44 | 33 | 657 | 0.0164 | 61 | 42 | 66.67 | 54.55 | 54.55 |
| *ycf1b* | 44 | 33 | 882 | 0.0350 | 156 | 110 | 71.11 | 66.67 | 60.61 |
| *matK+rbcL* | 44 | 33 | 1437 | 0.0158 | 150 | 105 | 73.33 | 66.67 | 57.58 |
| *matK+trnH-psbA* | 44 | 33 | 1456 | 0.0189 | 162 | 117 | 73.33 | 72.73 | 66.67 |
| *matK+ycf1b* | 44 | 33 | 1681 | 0.0265 | 257 | 185 | 86.67 | 75.76 | 75.76 |
| *rbcL+trnH-psbA* | 44 | 33 | 1295 | 0.0135 | 110 | 72 | 75.56 | 66.67 | 66.67 |
| *rbcL+ycf1b* | 44 | 33 | 1520 | 0.0225 | 205 | 140 | 86.67 | 87.88 | 81.82 |
| *ycf1b+trnH-psbA* | 44 | 33 | 1539 | 0.0266 | 217 | 152 | 91.11 | 78.79 | 66.67 |

Pi: Nucleotide diversity

S: Number of polymorphic site

**Table S9. Nucleotide diversity of *ycf1* b together with three other markers and their species-discriminating powers in *Paeonia* sect. *Moutan* (Paeoniaceae).**

| Locus | Numbers of samples | species | Matrix size(bp) | π | S | Parsimony informative sites | DNA barcoding (%) | | |
|---|---|---|---|---|---|---|---|---|---|
| | | | | | | | Blast | Tree-building | Distance |
| *matK* | 25 | 8 | 585 | 0.0051 | 15 | 9 | 38.46 | 25.00 | 12.50 |
| *rbcL* | 25 | 8 | 641 | 0.0023 | 9 | 2 | 11.54 | 12.50 | 12.50 |
| *trnH-psbA* | 19 | 8 | 405 | 0.0041 | 13 | 6 | 89.47 | 25.00 | 12.50 |
| *ycf1b* | 25 | 8 | 836 | 0.0055 | 20 | 14 | 88.46 | 37.50 | 50.00 |
| *matK+rbcL* | 25 | 8 | 1226 | 0.0051 | 24 | 11 | 46.15 | 12.50 | 12.50 |
| *matK+trnH-psbA* | 25 | 8 | 990 | 0.0051 | 28 | 15 | 80.77 | 25.00 | 12.50 |
| *matK+ycf1b* | 25 | 8 | 1421 | 0.0053 | 35 | 23 | 88.46 | 37.50 | 50.00 |
| *rbcL+trnH-psbA* | 25 | 8 | 1046 | 0.0023 | 22 | 8 | 65.38 | 12.50 | 12.50 |
| *rbcL+ycf1b* | 25 | 8 | 1477 | 0.0041 | 29 | 16 | 88.46 | 25.00 | 37.50 |
| *ycf1b+trnH-psbA* | 25 | 8 | 1241 | 0.0055 | 33 | 20 | 100.00 | 50.00 | 37.50 |

Pi: Nucleotide diversity

S: Number of polymorphic site

**Table S10. Nucleotide diversity of *ycf1* b together with three other markers and their species-discriminating powers in *Prunus* sect. *Armeniaca* (Rosaceae).**

| Locus | Numbers of samples | species | Matrix size(bp) | π | S | Parsimony informative sites | DNA barcoding (%) | | |
|---|---|---|---|---|---|---|---|---|---|
| | | | | | | | Blast | Tree-building | Distance |
| *matK* | 21 | 5 | 883 | 0.0014 | 5 | 4 | 52.38 | 60.00 | 20.00 |
| *rbcL* | 21 | 5 | 616 | 0.0007 | 1 | 1 | 0.00 | 0.00 | 0.00 |
| *trnH-psbA* | 21 | 5 | 400 | 0.0013 | 2 | 2 | 33.33 | 0.00 | 0.00 |
| *ycf1b* | 21 | 5 | 871 | 0.0063 | 16 | 14 | 100.00 | 100.00 | 100.00 |
| *matK+rbcL* | 21 | 5 | 1499 | 0.0011 | 6 | 5 | 57.14 | 60.00 | 20.00 |
| *matK+trnH-psbA* | 21 | 5 | 1283 | 0.0014 | 6 | 6 | 57.14 | 40.00 | 20.00 |
| *matK+ycf1b* | 21 | 5 | 1754 | 0.0040 | 21 | 18 | 100.00 | 100.00 | 100.00 |
| *rbcL+trnH-psbA* | 21 | 5 | 1016 | 0.0009 | 3 | 3 | 33.33 | 0.00 | 0.00 |
| *rbcL+ycf1b* | 21 | 5 | 1487 | 0.0041 | 17 | 15 | 100.00 | 100.00 | 100.00 |
| *ycf1b+trnH-psbA* | 21 | 5 | 1271 | 0.0049 | 18 | 16 | 100.00 | 100.00 | 100.00 |

Pi: Nucleotide diversity

S: Number of polymorphic site

**Table S11. Nucleotide diversity of *ycf1* b together with three other markers and their species-discriminating powers in *Quercus* (Fagaceae).**

| Locus | Numbers of samples | species | Matrix size(bp) | π | S | Parsimony informative sites | DNA barcoding (%) | | |
|---|---|---|---|---|---|---|---|---|---|
| | | | | | | | Blast | Tree-building | Distance |
| *matK* | 38 | 20 | 739 | 0.0027 | 11 | 10 | 5.26 | 10.00 | 5.00 |
| *rbcL* | 38 | 20 | 785 | 0.0046 | 19 | 13 | 21.05 | 25.00 | 25.00 |
| *trnH-psbA* | 30 | 16 | 479 | 0.0081 | 18 | 8 | 43.33 | 25.00 | 12.50 |
| *ycf1b* | 38 | 20 | 865 | 0.0075 | 41 | 31 | 36.84 | 45.00 | 40.00 |
| *matK+rbcL* | 38 | 20 | 1424 | 0.0037 | 30 | 23 | 26.32 | 30.00 | 25.00 |
| *matK+trnH-psbA* | 38 | 20 | 1218 | 0.0038 | 29 | 18 | 42.11 | 40.00 | 25.00 |
| *matK+ycf1b* | 38 | 20 | 1604 | 0.0052 | 52 | 41 | 39.47 | 45.00 | 40.00 |
| *rbcL+trnH-psbA* | 38 | 20 | 1264 | 0.0054 | 37 | 21 | 52.63 | 40.00 | 35.00 |
| *rbcL+ycf1b* | 38 | 20 | 1650 | 0.0061 | 60 | 44 | 42.11 | 45.00 | 45.00 |
| *ycf1b+trnH-psbA* | 38 | 20 | 1344 | 0.0074 | 59 | 39 | 52.63 | 45.00 | 40.00 |

Pi: Nucleotide diversity

S: Number of polymorphic site

**Table S12. Nucleotide diversity of *ycf1* b together with three other markers and their species-discriminating powers in *Panax* (Araliaceae).**

| Locus | Numbers of samples | species | Matrix size(bp) | π | S | Parsimony informative sites | DNA barcoding (%) | | |
|---|---|---|---|---|---|---|---|---|---|
| | | | | | | | Blast | Tree-building | Distance |
| *matK* | 23 | 8 | 818 | 0.0086 | 30 | 30 | 69.57 | 62.50 | 62.50 |
| *rbcL* | 23 | 8 | 637 | 0.0066 | 15 | 15 | 82.61 | 75.00 | 62.50 |
| *trnH-psbA* | 23 | 8 | 477 | 0.0143 | 31 | 23 | 73.91 | 50.00 | 50.00 |
| *ycf1b* | 23 | 8 | 876 | 0.0294 | 87 | 84 | 86.96 | 75.00 | 62.50 |
| *matK+rbcL* | 23 | 8 | 1455 | 0.0077 | 45 | 45 | 82.61 | 75.00 | 75.00 |
| *matK+trnH-psbA* | 23 | 8 | 1295 | 0.0106 | 61 | 53 | 73.91 | 62.50 | 50.00 |
| *matK+ycf1b* | 23 | 8 | 1694 | 0.0191 | 117 | 114 | 86.96 | 75.00 | 75.00 |
| *rbcL+trnH-psbA* | 23 | 8 | 1114 | 0.0097 | 46 | 38 | 82.61 | 62.50 | 62.50 |
| *rbcL+ycf1b* | 23 | 8 | 1513 | 0.0196 | 102 | 99 | 100.00 | 75.00 | 87.50 |
| *ycf1b+trnH-psbA* | 23 | 8 | 1353 | 0.0243 | 118 | 107 | 86.96 | 75.00 | 62.50 |

Pi: Nucleotide diversity

S: Number of polymorphic site
